# Plants That Evolved Under High Phylogenetic Diversity Have Higher Invasion Success, Particularly in Undisturbed Communities

**DOI:** 10.1111/ele.70417

**Published:** 2026-06-25

**Authors:** Joshua I. Brian, Mark van Kleunen, Wayne Dawson, Anne Kempel, Weihan Zhao, Jane A. Catford

**Affiliations:** ^1^ Department of Geography King's College London London UK; ^2^ Ecology, Department of Biology University of Konstanz Konstanz Germany; ^3^ Zhejiang Key Laboratory for Restoration of Damaged Coastal Ecosystems & Zhejiang Provincial Key Laboratory of Plant Evolutionary Ecology and Conservation Taizhou University Taizhou China; ^4^ Department of Evolution, Ecology and Behaviour, Institute of Infection, Veterinary and Ecological Sciences University of Liverpool Liverpool UK; ^5^ WSL Institute for Snow and Avalanche Research SLF Davos Switzerland; ^6^ Climate Change, Extremes and Natural Hazards in Alpine Regions Research Center CERC Davos Switzerland; ^7^ International Max Planck Research School for Quantitative Behaviour, Ecology and Evolution (IMPRS‐QBEE), Max Planck Institute of Animal Behaviour Konstanz Germany; ^8^ Fenner School of Environment & Society The Australian National University Canberra ACT Australia

**Keywords:** climate dissimilarity, community diversity, community invasibility, disturbance, establishment, evolutionary imbalance, non‐native plant invasion, phylogenetic relatedness, precipitation, seed addition

## Abstract

The evolutionary imbalance hypothesis predicts that species from ecologically stable regions of high genetic potential and intense competition are more likely to be invasive, while regions with the opposite characteristics are more likely to be invasible. Relative phylogenetic diversity (PD) of species' indigenous ranges could indicate evolutionary imbalance and help identify high‐risk invaders and vulnerable communities. We tested this with three seed addition experiments where 166 species of varying origins were sown into disturbed and undisturbed grassland plots. Species with high relative indigenous‐PD had high colonisation and first‐year survival regardless of disturbance, whereas species with low relative indigenous‐PD only colonised disturbed communities and at a lower rate. Species' indigenous‐PD did not appear to affect second‐year survival. Although long‐term outcomes are unknown, evidence suggests that species with high relative indigenous‐PD pose a high invasion risk, even to intact communities. Regional PD could help indicate species invasiveness and community invasibility, informing biosecurity.

## Graphical abstract

We show that species from phylogenetically diverse home ranges can colonise even undisturbed sites, highlighting evolutionary imbalance as a potentially influential driver of asymmetric invasion patterns worldwide. Our results have implications for the prediction and management of invasive species.

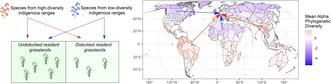

## 1 Introduction

Invasive species threaten biodiversity (Pyšek et al. 2020; Roy et al. 2024). Dozens of hypotheses have been proposed to explain why some species successfully invade while others do not. Although many hypotheses invoke changes that non‐native species experience when introduced to a new range, others suggest that species' inherent characteristics can make them more likely to successfully invade (Catford et al. 2009). One hypothesis that relies on species' inherent characteristics is the evolutionary imbalance hypothesis (EIH) (Fridley and Sax 2014). Invoking the importance of evolutionary legacies in shaping species' ecologies (Ricklefs et al. 1999; Cavender‐Bares et al. 2016), this hypothesis states that the degree to which species become ‘ecologically optimised’ to a set of environmental conditions increases with (i) genetic variation within populations, (ii) competition intensity and (iii) environmental stability and number of generations populations have experienced under those conditions (Fridley and Sax 2014). Regional phylogenetic diversity (PD) has been suggested as a proxy for these factors, as it represents the overall competitiveness of a region (Fridley and Sax 2014; Fristoe et al. 2023). Therefore, while not directly causal, ‘indigenous‐PD’ (overall phylogenetic diversity in the native, or indigenous, distribution of a species) may relate to the competitive environment that a species evolved in and—correspondingly—the competitive ability of that species. By extension, if introduced beyond their native range, species with high indigenous‐PD may be able to outcompete and displace species from less competitive regions, facilitating invasion (Fridley and Sax 2014; Enders et al. 2020; Fan et al. 2025). If the EIH is supported, indigenous‐PD could be used to identify source pools of high‐risk invaders and communities with high potential invasibility.

The EIH posits that species with high indigenous‐PD should be competitively superior for the environmental conditions they have evolved under, rather than for every possible set of environmental conditions (Fridley and Sax 2014). Therefore, it is important to consider environmental suitability when evaluating the EIH. Invading species typically have higher phylogenetic similarities to the native community than expected by chance, suggesting similar ecological requirements between natives and invaders (Qian and Sandel 2022; Dong et al. 2025). Under this scenario, invaders with high indigenous‐PD should be able to outcompete ecologically similar native species that originate from regions of low PD. However, invading species with high indigenous‐PD may be able to fill areas of niche space that native species find unsuitable (Fridley and Sax 2014); this would reduce competition and decrease phylogenetic similarity between natives and invaders (Cadotte et al. 2018). Species with high indigenous‐PD could potentially invade environmentally suitable regions, but also regions where they are phylogenetically dissimilar to natives.

Other drivers of invasion that affect competition and environmental suitability could moderate the importance of indigenous‐PD for invasion success. Three such drivers are disturbance, herbivory and climate change. Disturbance can facilitate invasion by reducing resource competition (van Kleunen et al. 2018), but the EIH implies that species from high‐PD regions should be able to invade even undisturbed environments through their superior competitive ability (Fridley and Sax 2014; Fridley et al. 2021). Therefore, disturbance may mainly benefit species from low‐PD regions. Herbivory can lower competition by increasing light availability (van der Wal et al. 2000; Borer et al. 2014). Species from high‐PD regions may therefore have a particular advantage over species from low‐PD regions in areas where herbivores have been lost and thus resource competition is more intense. Climate warming can alter the strength of competition depending on how it affects resources and resource uptake (White et al. 2001; Harrison 2020; Liu et al. 2022). More generally, climate shapes the pool of successful invaders, with native and non‐native species observed to be more phylogenetically similar in stressful climates (Park et al. 2020; Fan et al. 2023). As disturbance regimes, herbivore distributions and climate are all being affected by anthropogenic global change and can affect invasion through their effect on competition (Bradley et al. 2010; Price et al. 2022; Gioria et al. 2023), it is important to test how they interact with the mechanisms of the EIH.

To date, tests of the EIH have only occurred at the scales of countries or continents, using numbers of observed naturalised species (e.g., Fridley and Sax 2014; Fridley et al. 2021; Fristoe et al. 2023; Fan et al. 2025). Experimental tests at a community scale may provide important additional information. First, experimental seed addition can disentangle factors that help species successfully pass through ecological filters (*sensu* Pearson et al. 2018) at different invasion stages (Blackburn et al. 2011; van Kleunen et al. 2018). Observations of established non‐native species cannot distinguish between species that were introduced but failed to establish, and those that were not introduced (Bartlett et al. 2024), making it difficult to separate evolutionary imbalance from sampling or introduction bias in global‐scale studies (Moyano 2023; Fan et al. 2025). Second, experimental community‐level studies allow for fine‐scale characterisation of the PD of the resident community, capturing the immediate competitive environment experienced by an invader. By comparing indigenous‐PD of invading species to that of the resident community, the effect of relative PD on invasion success can be investigated, independent of factors like disturbance that may influence invasion over broader scales (Fridley et al. 2021; Moyano 2023). To our knowledge, there has never been a community‐scale experimental investigation of the EIH.

Here, we integrate data from three seed addition experiments to test the EIH at the community‐scale. We include both natives and non‐natives in our species pool to test the assumption that high indigenous‐PD should enhance successful invasion into a community (where a species is currently absent) regardless of whether that community is in the native or non‐native range of a species; this recognises that the indigenous‐PD of native species can also vary (i.e., they can each have different native ranges). We ask three questions:

1. Does high indigenous‐PD increase the likelihood of successful colonisation and survival of sown species? We answer this question using absolute indigenous‐PD, matching the metrics used by previous large‐scale studies of the EIH.
2. Does high *relative* indigenous‐PD increase the likelihood of successful colonisation and survival of sown species? We answer this question using a subset of the experiments for which the resident community was surveyed in detail, comparing the indigenous‐PD of the sown species to the average indigenous‐PD of the resident species in the plot. This explicitly tests whether asymmetry in the PD of recipient communities vs the indigenous‐PD of invaders predicts invasion success, as posited by the EIH.
3. Is high indigenous‐PD correlated with traits that could explain increased survival of sown species? A correlation between high indigenous‐PD and traits related to competitive ability could indicate that species from high‐PD regions develop certain trait combinations, providing a potential mechanism for the relationship between indigenous‐PD and invasion success.

We address these questions whilst accounting for the role of environmental suitability (phylogenetic similarity to resident community, climate similarity) and other invasion drivers (disturbance, herbivory, warming).

## 2 Methods

### 2.1 Experimental Design

**FIGURE 1:**
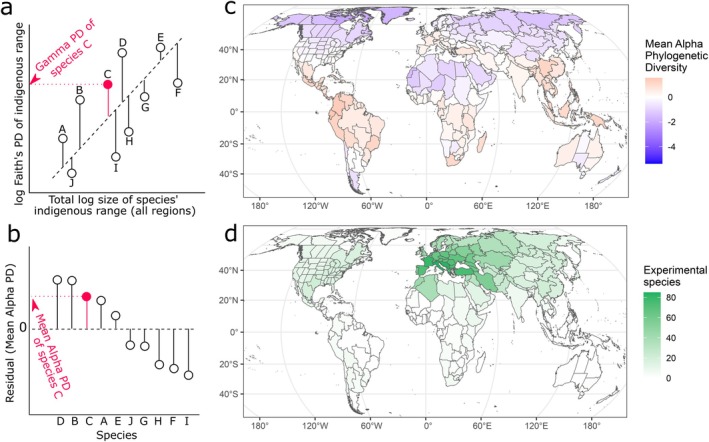
Calculation of indigenous Phylogenetic Diversitry (PD) for the 166 focal species in the experiments, and trends in global indigenous‐PD. (a) Overall Faith's phylogenetic diversity is calculated for each species, using the diversity from all TDWG3 regions in which a species is indigenous. Ten species are plotted as an example (species A–species J), with the total phylogenetic diversity in the indigenous range of species C highlighted (the gamma PD of species C). (b) Mean alpha PD (the focal metric in the study, hereon indigenous‐PD) is defined as the mean area‐corrected PD across the indigenous range of a species. It is calculated as the residual of the log–log relationship between native range size and gamma PD (species C highlighted as an illustration). (c) Area‐corrected mean alpha PD for each TDWG3 biogeographic region (*N* = 369 regions), illustrating global trends in indigenous‐PD. Red regions (mean alpha PD > 0) have a higher phylogenetic diversity than would be expected given their area, while blue regions (mean alpha PD < 0) have a lower phylogenetic diversity than would be expected given their area. (d) The number of focal species (planted in experiments in the present study) native to each TDWG3 region.

We leverage data from three seed addition experiments in Central Europe (Kempel et al. 2013; Müller et al. 2016; Haeuser et al. 2017), henceforth ‘Kempel,’ ‘Müller’ and ‘Haeuser’. All experiments added seeds of both native and non‐native species into natural or semi‐natural existing vegetation. In total, there were 166 unique species including 64 native and 102 non‐native species (Figure 1d; Table S1). For each of the Kempel and Haeuser experiments, seeds were sown into plots (with discrete subplots for different species) and then individual plants were tracked across three and two growing seasons respectively. Müller constituted two sub‐experiments: in the first, seeds were sown and colonisation success was recorded approximately 6 weeks later; in the second, seedlings were planted and survival was tracked over two growing seasons, with different subplots for each species in each case.

All studies involved a disturbance treatment, where half the plots in each study were hand‐tilled to a depth of 5–15 cm prior to seed sowing. Haeuser also included a heating treatment using suspended infra‐red heating lamps, and the second Müller sub‐experiment included a herbivory treatment, where half the plots were protected from herbivory using 1.8 m high cages covered in insect netting. Complete experimental designs, species per experiment and replication are summarised in Figures S1–S3.

### 2.2 Indigenous Phylogenetic Diversity of Sown Species

To quantify the phylogenetic diversity of the study species' indigenous ranges, we used the 369 regions of the TDWG Level 3 World Geographical Scheme for Recording Plant Distributions (Brummitt 2001). For each region, we calculated Faith's Phylogenetic Diversity (PD) based on the native plants in that region according to the Plants of the World Online database (POWO 2019) (Figure 1c). For each of the 166 sown species, we calculated mean alpha PD, a measure of indigenous‐PD representing the mean PD across all regions in a species' native range (following Fristoe et al. 2023; Figure 1b, Figure S4). This metric is area‐adjusted to correct for inequality in region size, considering larger regions may contain more species without necessarily being more competitive environments (Figure 1a). It therefore takes negative values in cases where indigenous‐PD is lower than expected based on indigenous range size. We also calculated indigenous‐PD for each species in the recipient community in Haeuser (an additional 63 species), as the recipient community was quantitatively sampled in that study. Although we focus on grasslands, we include woody plants in our calculations of indigenous‐PD because woody and non‐woody species could still interact over evolutionary time (Strömberg 2011). However, we also confirmed that the proportion of tree species in species' native ranges does not bias indigenous‐PD (see Supporting Information: Methods; Figure S5).

We also calculated three other metrics of indigenous‐PD: maximum alpha, median alpha, and gamma PD (Figure S4). All metrics gave consistent results and were generally well correlated (Figure S6). We expect that mean alpha PD best captures our desired ecological signal, as it reflects the average diversity that a given plant experiences. In addition, mean alpha PD had the highest average correlations with other metrics (Figure S6). We present mean alpha PD as our measure of indigenous‐PD in the main results, with the other three metrics in the Supporting Information: Results.

We recognise that using the geopolitical TDWG3 system to define regions could lead to bias (Murphy 2021); however, our metrics account for uneven size between regions so this bias should be limited. Further studies of the EIH may benefit from the development of datasets that better capture the evolutionary context of a species.

### 2.3 Climate Dissimilarity

For each sown species, we calculated the climate dissimilarity between its indigenous range and the experimental locations, as the benefits of high indigenous‐PD may be conditional on a sufficiently similar climate. We downloaded a subset of global bioclimatic variables from WorldClim at 10‐min resolution (bio1, bio4–bio6, bio10–bio17, following Pérez‐Navarro et al. 2022) and reduced dimensionality using PCA. We extracted the first two axes (79.1% of total variation explained), which correlated to temperature and its seasonality (PC1) and precipitation and its seasonality (PC2). For each species, we calculated average PC1 and PC2 values from raster cells where it had a native occurrence (GBIF 2025) and then calculated the difference between those indigenous climates and values for experimental locations. We used the absolute values of these PC differences to produce two explanatory variables: ‘Temperature dissimilarity’ and ‘Precipitation dissimilarity’. Higher values indicate more dissimilar indigenous climates and experimental climates (Figure S7).

### 2.4 Response Variables

We included measurements of our study species at four life stages: colonisation (approximately 6 weeks after seeds were planted), first year (end of the first growing season), overwinter (start of the second growing season) and second year (end of the second growing season). At each life stage, we looked at both a binary measure of whether *any* plants of each species survived in a given subplot, and the number of surviving plants per species for subplots that had at least one surviving individual. There were therefore eight response variables in total (four time periods and two measures per period). The response variables were contingent on having successfully survived the previous life stage (e.g., first year contingent on colonisation, etc.). The Müller sub‐experiments were not surveyed at the end of the second year, and so the fourth life stage only included data from Kempel and Haeuser.

### 2.5 Statistical Analysis

We structure our analyses by our three research questions. For all analyses, we used generalised linear mixed‐effects models (‘glmmTMB’ package, Brooks et al. 2017) in R v.4.1.1 (R Core Team 2021). Model assumptions were tested using the ‘DHARMa’ (Hartig 2022) and ‘performance’ (Lüdecke et al. 2021) packages, and family and link functions were chosen based on diagnostic results. Results were visualised using ‘ggplot2’ (Wickham 2016) and ‘interactions’ (Long 2024).

#### 2.5.1 Question 1

To test whether high indigenous‐PD increases the likelihood of survival and the number of plants surviving across life stages, we used the full Kempel, Müller and Haeuser dataset. Indigenous‐PD was the focal explanatory variable. Other explanatory variables included as potential confounders were disturbance (undisturbed/disturbed), temperature and precipitation dissimilarity, and herbivory (yes/no). Herbivory was only included for the first year and overwinter, as these are the two periods covered in the second Müller sub‐experiment which included a herbivory treatment (Figure S3). We assumed all Kempel and Haeuser subplots were subject to herbivory. At each life stage, we modelled binary success using the binomial family (logit link), and number of successes using the negative binomial family (log link). We standardised continuous variables by mean‐centering and dividing by two standard deviations, as this enables more direct comparisons of coefficients between continuous and binary variables (Gelman 2008). For each model, we included all explanatory variables as fixed effects and kept them in the model regardless of significance (e.g., to account for any variation caused by climate dissimilarity, even if climate dissimilarity is not significant). We also tested the interaction of indigenous‐PD with other explanatory variables; we only maintained significant interactions as including non‐significant interactions substantially reduced model fits. We also included the total number of seeds added and seed addition rate (seeds cm^−2^) as fixed effects, to account for differences in both seed rate and subplot size between studies. This was necessary as exploratory models using proportion of plants surviving as the response failed to meet model assumptions. Random effects were plot nested within study, and species nested within family.

#### 2.5.2 Question 2

To test whether higher relative PD increases the likelihood of colonisation and survival across life stages, we used the Haeuser experiment, because this study recorded the cover of every species in the resident community. We calculated the difference between the indigenous‐PD of each sown species and the average abundance‐weighted indigenous‐PD of residents in each subplot. Therefore, the focal explanatory variable was ‘relative indigenous‐PD’, reflecting the extent to which sown species have more phylogenetically diverse indigenous ranges than the species in the resident community. These models included disturbance and climate dissimilarity as well as whether the subplots were heated (heating vs. no heating); and abundance‐weighted mean phylogenetic distance (‘phylogenetic distance’) between the sown species and the recipient community, with higher phylogenetic distances representing lower phylogenetic similarity. Haeuser et al. (2017) also recorded the number of flowers produced by sown species, so we included both binary (flowering yes/no) and count (number of flowers) measures per subplot as an additional response variable. As no plants flowered in undisturbed subplots, these models included data from disturbed subplots only. We modelled binary measures of success at each time point using the binomial family (logit link), and number of successes using the Poisson family (log link), treating interactions as described above. Random effects were species nested within family.

To test the robustness of our results, we ran additional models for all Q1 and Q2 analyses testing different data subsets and verified that our trends held for species from different global biogeographic regions, and for species that were native to the experimental region as well as for species that were non‐native to the experimental region (see Supporting Information: Methods). We only report trends which were supported by robustness tests and by multiple PD metrics. Details are provided in Supporting Information: Results (Figures S9–S16, Tables S2–S10).

#### 2.5.3 Question 3

To explore the positive effect of sown species indigenous‐PD, we tested correlations between indigenous‐PD and plant traits where data was available (Kempel et al. 2013; Haeuser et al. 2017; Kattge et al. 2020; Falster et al. 2021), averaging individual plant measurements to the species level. We tested relationships with seed mass, vegetative and generative height, leaf N, SLA, LDMC, life history (annual vs. perennial), winter hardiness index, optimal germination rate, resistance to herbivory and competition index, with the latter three determined in separate greenhouse experiments (Kempel et al. 2013; Haeuser et al. 2017). All models used general linear models (Gaussian family) except optimal germination rate (binomial family, logit link), with response variables logged where appropriate to meet assumptions.

To test whether species traits can fully explain the positive effect of indigenous‐PD, or whether indigenous‐PD provides a more holistic proxy for species success, we ran structural equation models (SEMs) using piecewiseSEM (Lefcheck 2016). We either included indirect effects of indigenous‐PD only (i.e., effect of indigenous‐PD mediated entirely through plant traits) or included both direct and indirect effects and compared the competing models using AIC (Figure S8). We used SLA, vegetative height and seed mass for SEMs as they all load strongly on to the aboveground spectrum of plant form and function (Díaz et al. 2016), are among the most measured traits for naturalised species (Grenié et al. 2025) and had the greatest coverage in our dataset. For full details see Supporting Information: Methods.

## 3 Results

### 3.1 Q1: Do Sown Species With Higher Indigenous‐PD Have Higher Rates of Colonisation and Survival Than Species With Low Indigenous‐PD?

**FIGURE 2:**
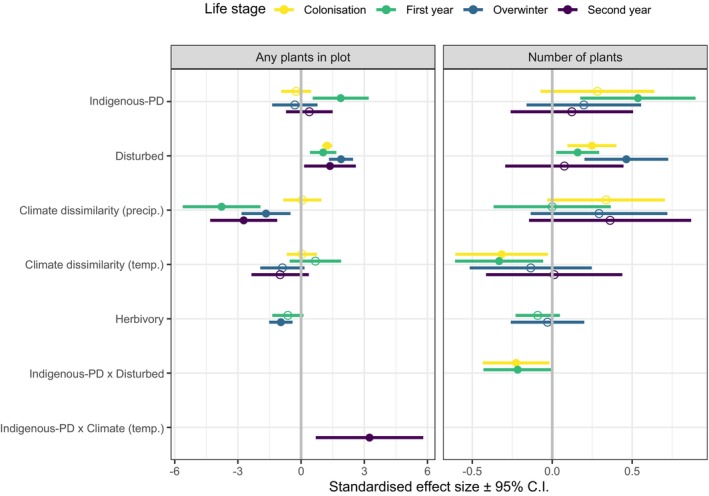
Summary of standardised effect sizes (estimate ±95% Confidence Interval) for models testing the effect of absolute indigenous‐PD (mean alpha PD) on species success, either binary success (left panel) or number of successes (right panel) in a plot, for each of four life stages. Open circles indicate variables that were not statistically significant; closed circles indicate statistically significant variables. Effect sizes less than zero indicate the variable reduced the likelihood of plant survival (left) or number of survivors (right) at each time point, while effect sizes greater than zero increased survival likelihood or number of survivors. Herbivory treatment was only available for the first year and overwinter life stages. Only statistically significant interactions were maintained in the model. Effect sizes were standardised by mean‐centering and dividing continuous variables by 2 standard deviations to facilitate direct comparison among variables, following Gelman (2008). The statistical significance of each variable was assessed individually using a *t*‐test, so in total 56 tests were done (tests for seven variables at each of four life stages for the two panels).

**FIGURE 3:**
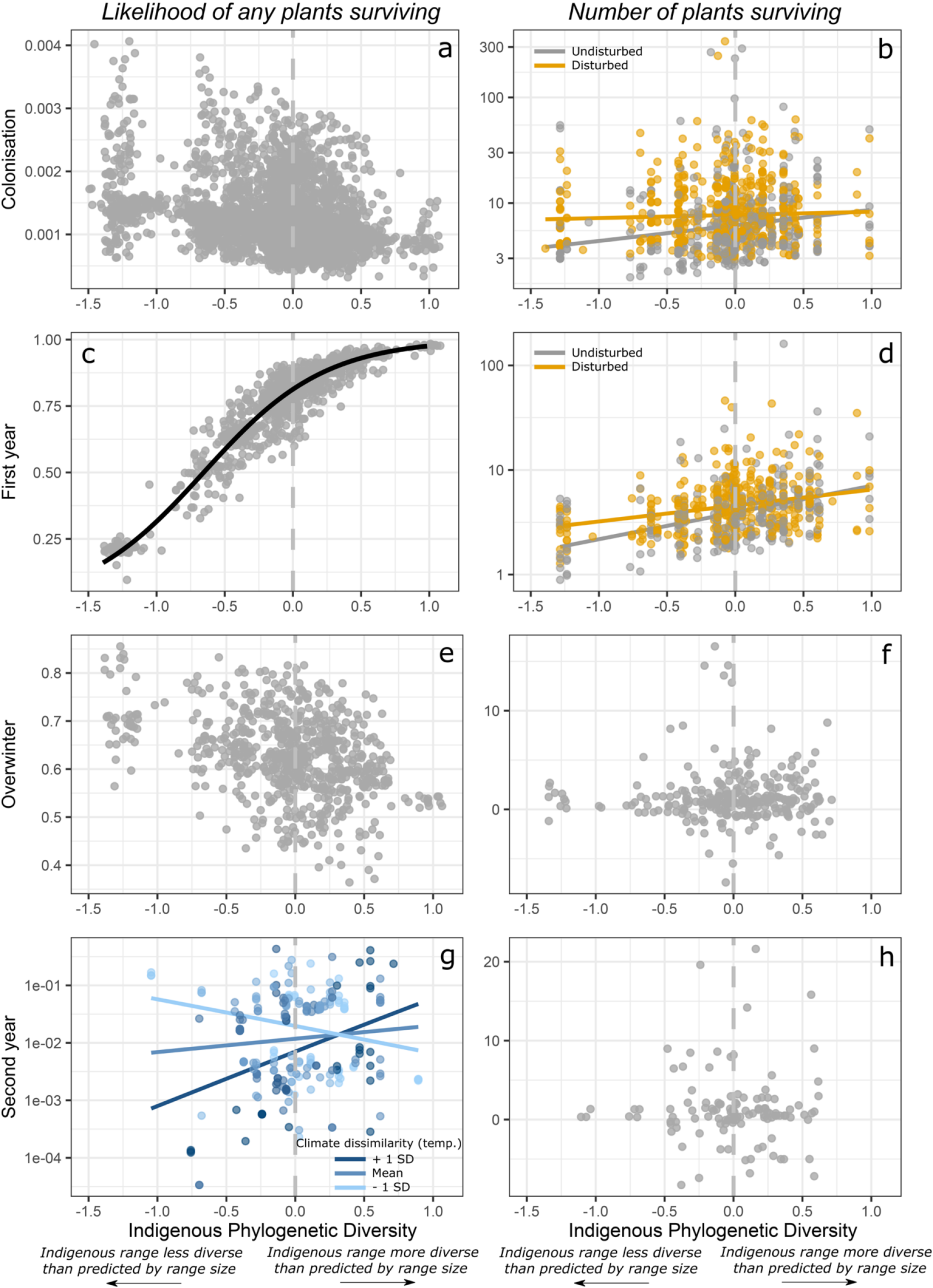
Marginal effects plot showing relationships between indigenous‐PD (mean alpha PD) and likelihood of any plants surviving (left column) and number of plants surviving (right column) for each of four life stages—colonisation, first year, overwinter and second year (rows, top to bottom). Plotted points represent residuals of observed data with all other fixed and random effects accounted for, including the different numbers of seeds sown in the different experiments. For climate dissimilarity (panel g), darker points indicate a more dissimilar climate; regression lines are shown for mean climate dissimilarity and dissimilarity values one standard deviation above (darker) and below (lighter) the mean. Each point represents one species in a given subplot. Solid lines indicate a statistically significant relationship (see Figure 2), while no lines indicate no statistical significance.

Species with high indigenous‐PD were more likely to have individuals survive to the end of the first year than species with low indigenous‐PD (Figures 2 and 3c). However, indigenous‐PD did not appear to affect the likelihood of colonisation, overwinter survival or second year survival (Figure 3a,e,f,h). High indigenous‐PD interacted with disturbance to increase the number of colonising individuals and individuals surviving the first year (Figures 2 and 3b,d). For species with low indigenous‐PD, the number of plants surviving was higher in disturbed subplots than in undisturbed subplots. However, for species with high indigenous‐PD, there was no difference between disturbed and undisturbed subplots (Figure 3b,d). In other words, species from phylogenetically diverse indigenous ranges did not need disturbance to achieve higher rates of colonisation and first year survival. However, there was no apparent relationship between PD and overwinter survival (Figure 3e,f).

Species with greater climate mismatches had lower chances of survival, particularly for precipitation dissimilarity when considering the likelihood of any plants surviving, and for temperature dissimilarity when considering the number of plants surviving (Figure 2). However, if a species had survived to the end of the first year in a subplot then the likelihood of any individuals of that species surviving overwinter to the second year also depended on a positive interaction between mean alpha PD and temperature dissimilarity: species with high indigenous‐PD were more likely to survive if they came from a more *dissimilar* climate (Figure 3g).

### 3.2 Q2: Do Sown Species With Higher Relative Indigenous‐PD Have Higher Rates of Colonisation and Survival Than Species With Lower Relative Indigenous‐PD?

**FIGURE 4:**
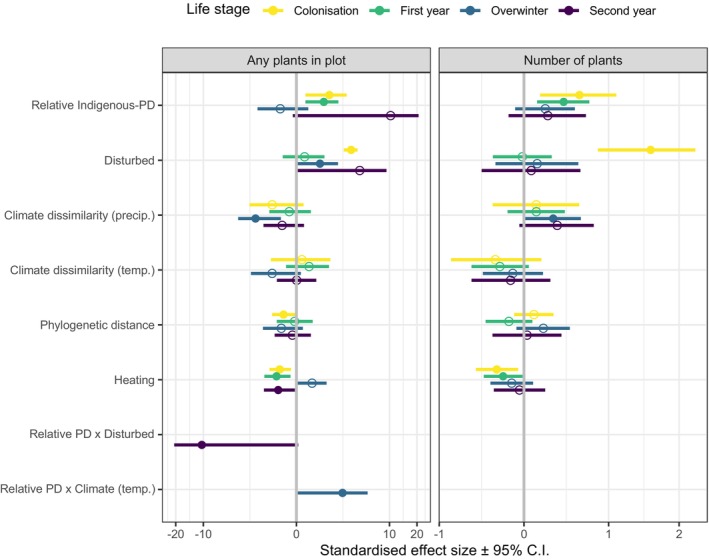
Summary of standardised effect sizes (estimate ±95% Confidence Interval) for models testing the effect of relative indigenous‐PD (difference between indigenous‐PD of sown species and abundance‐weighted mean of recipient community) on species success, either binary success (left panel) or number of successes (right panel) in a plot, for each of four life stages. Open circles indicate variables that were not statistically significant; closed circles indicate statistically significant variables. Effect sizes less than zero indicate the variable reduced the likelihood of plant survival (left) or number of survivors (right) at each time point, while effect sizes greater than zero increased survival likelihood or number of survivors. Note that both interactions were marginal (temperature dissimilarity interaction, *p* = 0.053; disturbance interaction, *p* = 0.052), but in both cases their inclusion improved the model fit and so were maintained. Effect sizes were standardised by mean‐centering and dividing continuous variables by 2 standard deviations to facilitate direct comparison among variables, following Gelman (2008). The statistical significance of each variable was assessed individually using a *t*‐test, so in total 64 tests were done (tests for eight variables at each of four life stages for the two panels).

**FIGURE 5:**
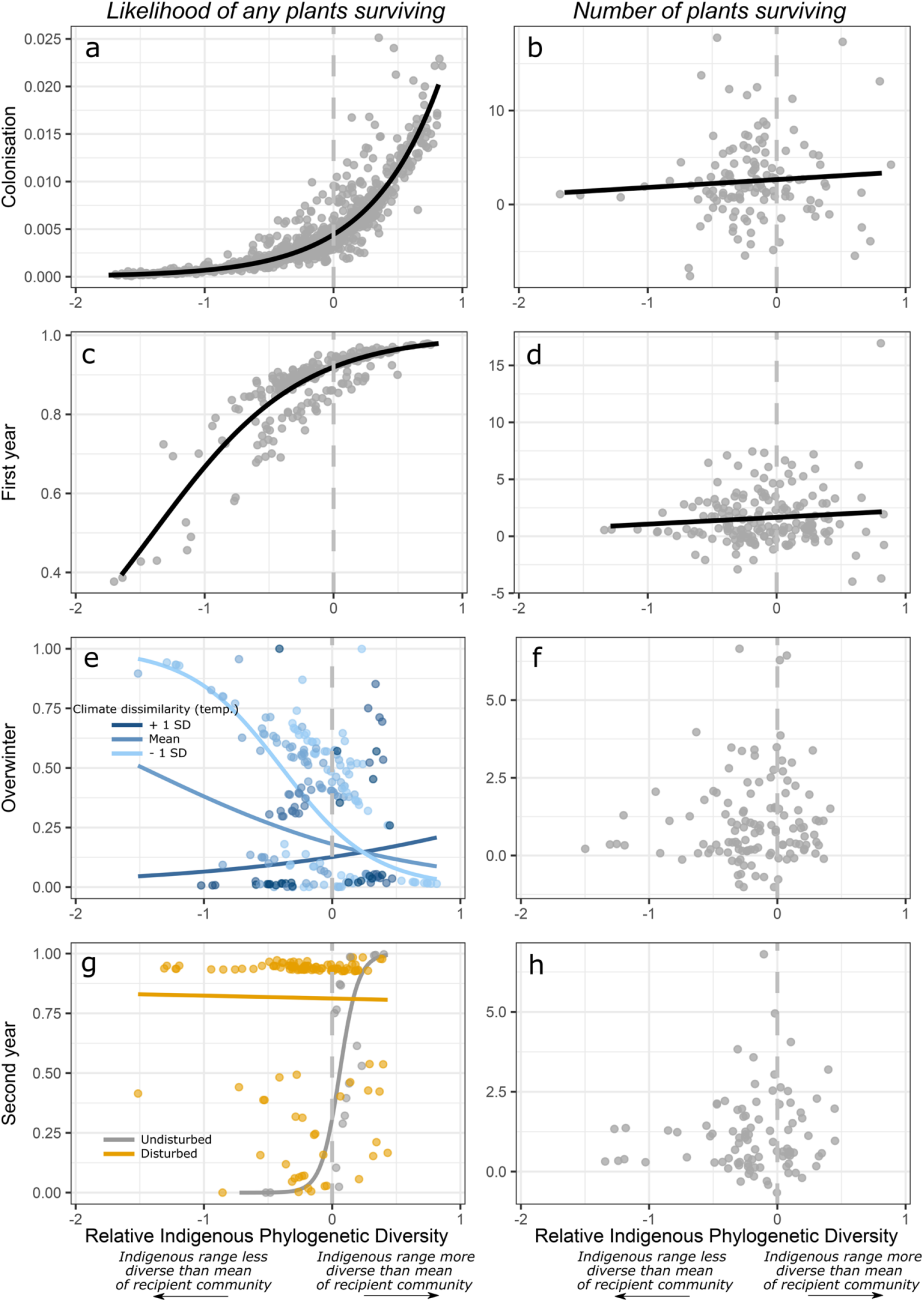
Marginal effects plot showing the difference between the indigenous‐PD of the sown species and the abundance‐weighted mean indigenous‐PD of the species in the recipient community (relative indigenous‐PD), and how this is related to the likelihood of any plants surviving (left column) and number of plants surviving (right column) for each of four life stages—colonisation, first year, overwinter and second year (rows, top to bottom). Plotted points represent residuals of observed data with all other fixed and random effects accounted for. For climate dissimilarity (panel e), darker points indicate a more dissimilar climate; regression lines are shown for mean climate dissimilarity and dissimilarity values one standard deviation above (darker) and below (lighter) the mean. Each point represents one species in a given subplot. Solid lines indicate a statistically significant relationship (see Figure 4), while no lines indicate no statistical significance.

When sown species came from more phylogenetically diverse indigenous ranges than species in the resident community, they were more likely to colonise and survive through the first year, and in greater numbers (Figures 4 and 5a–d). In general, disturbance increased the likelihood of plant colonisation (Figure 4), but species with high relative indigenous‐PD had a marginally higher likelihood of survival in the second year regardless of disturbance (Figure 5g). While sample size was small by the end of the second year of the Haeuser experiment, it is striking that sown species could only survive in undisturbed subplots when their indigenous range was more phylogenetically diverse than the average of the subplots they were invading (relative PD > zero; Figure 5g). However, we did not detect a benefit of high relative indigenous‐PD on the number of plants surviving in the second year (Figure 5f,h).

Species from more dissimilar precipitation regimes had a lower overall likelihood of survival overwinter, but those that did survive had a higher number of plants surviving if they were from more dissimilar precipitation regimes (Figure 4). The negative relationship with precipitation dissimilarity was evident for species coming from both wetter and drier climates than the experimental climate (Figure S17). Temperature dissimilarity also interacted with relative PD for overwinter survival (Figure 5e) as per Q1. Species from more phylogenetically diverse indigenous ranges than the recipient community only had higher likelihoods of survival if their indigenous climate was dissimilar in temperature to the experimental climate (Figure 5e).

There was no statistically significant relationship between relative indigenous‐PD and flowering, either on flowering likelihood or the number of flowers produced (Tables S6–S9). However, if species did flower, they were more likely to produce more flowers if they came from more dissimilar precipitation regimes (Tables S6, S7 and S9).

For both Q1 and Q2, trends were consistent for native and non‐native sown species as well as for species from different biogeographic regions: adding species native status or origin into models did not change the significance or interpretation of indigenous‐PD in any model (Table S10). Indeed, origin added little information, with models including native/non‐native status or biogeographic origin falling within 2 AIC of the main models in 28 of 32 cases (Table S10).

### 3.3 Q3: Is High Indigenous‐PD Correlated With Traits That Could Explain Increased Survival of Sown Species?

**FIGURE 6:**
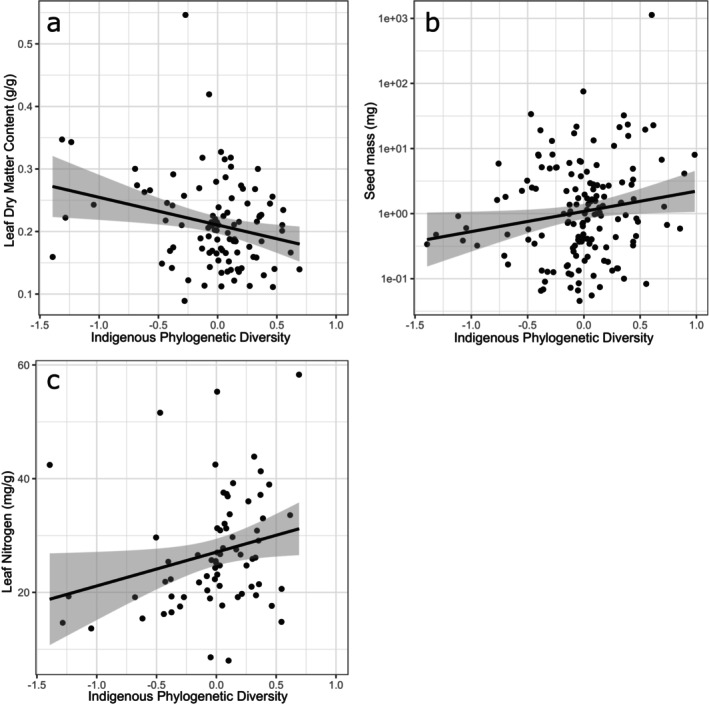
Relationship between indigenous‐PD (mean alpha PD) and (a) leaf dry matter content (*N* = 100); (b) seed mass (*N* = 153); (c) leaf nitrogen concentration (*N* = 70). Different sample sizes are due to the differing availability of trait data for each species. For full statistical results and results for other PD metrics see Table S12. Solid lines indicate a statistically significant relationship.

Species with high indigenous‐PD tended to have heavier seeds, higher leaf N and lower LDMC (Figure 6; all *p* < 0.05), a trend that was maintained when only including species from temperate indigenous ranges (Table S12). No other traits that we examined had statistically significant relationships with indigenous‐PD (Table S12). However, there was still a direct positive relationship between indigenous‐PD and invasion success even after inclusion of key traits in structural equation models (Table S11).

## 4 Discussion

In our grassland experiments, sown species that evolved in regions with high phylogenetic diversity (high indigenous‐PD) experienced greater colonisation and first year success, especially when sown into undisturbed communities, than species from less diverse regions. These findings lend support to the evolutionary imbalance hypothesis (EIH, Fridley and Sax 2014). The beneficial effects of high indigenous‐PD were mainly seen in the first year (Figures 2, 3, 4, 5), though interactions with disturbance and temperature dissimilarity persisted in the second year (Figures 3g and 5e,g, Figures S11b,c, S12 and S14d). These results were consistent even when accounting for climate dissimilarity, phylogenetic relatedness to resident species, plot warming and herbivory, and were robust to different analytical approaches (Tables S2–S11). The benefit of higher indigenous‐PD did not differ between sown species that were native or non‐native, or between species from different biogeographic ranges (Table S10). This suggests that high indigenous‐PD provides a general competitive benefit regardless of the specific location of a species' range. In line with the EIH, our findings suggest that communities composed of species with low indigenous‐PD may be particularly vulnerable to invasion by species originating from regions with high PD.

Disturbance can alleviate competition by increasing microsite and soil resource availability (Catford et al. 2023), which helps explain why disturbed communities are especially susceptible to invasion (van Kleunen et al. 2018). However, our results show that species with high indigenous‐PD attain the same numbers of colonists and first‐year recruits in both disturbed and undisturbed subplots (Figure 3b,d). While invaders with low indigenous‐PD are heavily reliant on disturbance for colonisation and first‐year survival, invaders from high‐PD regions pose a risk even to intact communities. The relative PD analysis adds nuance by highlighting that sown species can only survive through the second year in intact communities if they have a higher indigenous‐PD than species in the recipient community (Figure 5g). This strongly suggests that higher relative indigenous‐PD conveys a tangible competitive advantage. These trends align with those of Fridley et al. (2021), who found that high indigenous‐PD was linked to invasiveness in mainly undisturbed habitats in New Zealand, though their results were observational. Our results thus provide fine‐scale experimental support for the EIH, complementing country‐level support previously found (Fridley et al. 2021; Fristoe et al. 2023; Fan et al. 2025).

We also found that sown species from more similar precipitation regimes had higher likelihoods of success in colonisation and first‐year survival (Figures 2, 4), aligning with previous research showing that climate suitability is a key driver of invasion success on global scales (Liu et al. 2020; Fristoe et al. 2023; Fan et al. 2025). Temperature also interacted with indigenous‐PD: high indigenous‐PD only increased likelihood of survival into the second year if sown species came from climates with *less* similar temperatures (Figures 3g and 5e). We cautiously suggest that this may be because precipitation enforces a stronger filter than temperature (Figures 2 and 4): precipitation needs to be similar enough for species to be able to survive, but temperature is less limiting, and so higher indigenous‐PD can help compensate for any mismatches.

Our results also suggest that invading species need to be similar enough to natives to suit the local environment but different enough to exploit unoccupied niche space (Divíšek et al. 2018). For example, precipitation dissimilarity reduced the likelihood of any plants in a plot surviving (Figures 2, 4) and occasionally limited the benefit of higher indigenous‐PD (Figures S9d, S15b, S16a,b). However, if plants were able to survive, precipitation dissimilarity *increased* the number of plants surviving in the plot overwinter (Figure 4) and increased the benefit of higher indigenous‐PD (Figures S10d, S13b, S13d, S14e). Higher precipitation dissimilarity also increased the number of flowers on plants that successfully flowered (Tables S6, S7, S9). Higher phylogenetic distance between planted species and the resident community also reduced the likelihood of any plants colonising but was unrelated to the number of plants in plots where individuals did survive (Figure 4). These results support previous research that potential invaders need to be similar to native species to have a chance of surviving (Pinto‐Ledezma et al. 2020; Qian and Sandel 2022) but are more likely to become abundant and spread if they are different (Divíšek et al. 2018; Omer et al. 2022).

Plants with higher indigenous‐PD tended to have higher seed mass, higher leaf N content and lower LDMC (Figure 6). These relationships could potentially be confounded: for example, tropical regions have both higher seed mass (Moles et al. 2007) and higher indigenous‐PD (Figure 1c) due to favourable climates. However, the PD‐trait correlations were maintained when accounting for biogeography (Table S12). High indigenous‐PD could plausibly lead to certain trait values that increase species performance. Species evolving in high‐PD regions may produce seeds with larger storage (Figure 6b), which could reduce dependence on local resources that may be scarce. This allows successful germination and seedling establishment even under highly competitive conditions (Kidson and Westoby 2000) and could explain the strong observed relationship between high indigenous‐PD and colonisation success in undisturbed communities. High leaf N and low LDMC are both associated with rapid resource acquisition and growth (Pyšek and Richardson 2007; Díaz et al. 2016), suggesting that species with high indigenous‐PD evolve to be strong resource competitors (Figure 6a,c) and can tolerate competition in intact communities (Golivets and Wallin 2018). We hypothesise that high indigenous‐PD species lie on a superior trade‐off surface to low indigenous‐PD species (Catford et al. 2018) and can produce high‐value seeds while still investing in rapid growth and resource acquisition. However, the benefits of high indigenous‐PD cannot be explained purely by species traits (Table S11), at least not the three traits that had sufficient data coverage to be included in our structural equation models. Indigenous‐PD potentially provides a broad proxy for evolutionary advancement (Fridley and Sax 2014) that captures trait suites and other evolutionarily derived fitness benefits.

While our colonisation and first year results support the EIH, there was less evidence for sustained benefits in the second year. We did not see any relationship between high indigenous‐PD and the number of surviving plants in the second year (Figures 3f,h, 5f,h), nor could we show any impacts of sown species, as a single survey of the resident vegetation was conducted in the Haeuser study only. Therefore, we did not find evidence to suggest that high indigenous‐PD invaders displace other species or reduce their fitness, which is required to show competitive superiority (Begon et al. 1986; Tilman 1987). However, our inferences in the second year are limited by sample size. For example, the likelihood of any plants flowering was 2.7 times higher for each unit increase in mean alpha relative indigenous‐PD (Table S6), but the low numbers of plants surviving to flowering may have limited statistical power. Nevertheless, even if evolutionary imbalance is not directly related to species' spread or impact, it may still be important for invaders to gain a foothold in their new ranges. Even small effect sizes at our subplot level could have large impacts at the population level over larger scales (Orr et al. 2021).

## 5 Conclusion

To our knowledge, this is the first study to experimentally test the evolutionary imbalance hypothesis, and to test it at the community‐scale. Species with higher relative indigenous phylogenetic diversity (PD) were better at colonising undisturbed communities—potentially through superior trait combinations that increased their likelihood of post‐germination survival and ability to obtain resources in competitive environments. However, we found less evidence for population spread. Long‐term experiments will be critical for clarifying the role of evolutionary imbalance in the ultimate success and impact of invaders. Our findings nevertheless suggest that regions with high phylogenetic diversity may be sources of particularly insidious invaders and regions with low phylogenetic diversity may be particularly vulnerable to such invaders (Figure 1c). This may help explain why oceanic islands are so vulnerable to invasion (van Kleunen et al. 2015). Our results could inform biosecurity and invasive species management. For example, the phylogenetic diversity of donating regions could be integrated into risk assessments (Roy et al. 2015). Our results suggest that specific origin‐recipient region combinations hold importance for the prediction and management of invasive species, whereby invaders with relatively high indigenous‐PD would pose a greater risk.

## Author Contributions

Jane A. Catford conceived the idea, and Joshua I. Brian and Mark van Kleunen further developed ideas. Mark van Kleunen, Wayne Dawson and Anne Kempel designed experiments and collected data. Weihan Zhao created the complete phylogenetic tree. Joshua I. Brian analysed data and drafted the manuscript. All authors interpreted the results and contributed to paper writing.

## Funding

This work was supported by H2020 European Research Council, 101002987.

## Supporting information


**Figure S1:** Experimental design of Haeuser et al. 2017.
**Figure S2:** Experimental design of Kempel et al. 2013.
**Figure S3:** Experimental design of Müller et al. 2016.
**Figure S4:** Calculating metrics of phylogenetic diversity (PD).
**Figure S5:** The indigenous‐PD for a given species is not related to the proportion of tree species in its indigenous range (*p* = 0.429).
**Figure S6:** Correlations between the phylogenetic diversity metrics used in the study.
**Figure S7:** Histograms showing the climate dissimilarity (experimental climate minus native range climate) for all species in the experiments, where ‘count’ refers to the number of species in each climate bin.
**Figure S8:** Structure of the two competing structural equation models (SEMs) tested for each model.
**Figure S9:** Marginal effects plot showing interactions between maximum alpha PD and disturbance on metrics of seeded species success.
**Figure S10:** Marginal effects plot showing interaction between median alpha PD, disturbance and climate dissimilarity on metrics of seeded species success.
**Figure S11:** Marginal effects plot showing interaction between gamma PD, disturbance and temperature dissimilarity on metrics of seeded species success.
**Figure S12:** Marginal effects plot showing interaction between disturbance and (a) mean, (b) maximum and (c) gamma PD on the likelihood of species surviving to the end of the second growing season.
**Figure S13:** Marginal effects plot showing interaction between maximum alpha PD difference and climate dissimilarity or disturbance on plant responses at various time points (Haeuser data).
**Figure S14:** Marginal effects plot showing interaction between median alpha PD and climate dissimilarity or disturbance on plant responses at various time points (Haeuser data).
**Figure S15:** Marginal effects plot showing interaction between gamma alpha PD and climate dissimilarity or disturbance on plant responses at various time points (Haeuser data).
**Figure S16:** Marginal effects plot showing interaction between climate dissimilarity and (a) mean, (b) maximum and (c) median PD on the likelihood of overwinter plant survival, when using the simpler K+H difference in PD metric.
**Figure S17:** The negative relationship between precipitation dissimilarity or temperature dissimilarity and likelihood of overwinter and second‐year survival is quadratic.
**Table S1:** Complete list of all species across all three experiments.
**Table S2:** Summary of results, using *mean alpha phylogenetic diversity* as the response variable.
**Table S3:** Summary of results, using *maximum alpha phylogenetic diversity* as the response variable.
**Table S4:** Summary of results, using *median alpha phylogenetic diversity* as the response variable.
**Table S5:** Summary of results, using *gamma phylogenetic diversity* as the response variable.
**Table S6:** Summary of results, using *difference in mean alpha phylogenetic diversity* as the response variable.
**Table S7:** Summary of results, using *difference in maximum alpha phylogenetic diversity* as the response variable.
**Table S8:** Summary of results, using *difference in median alpha phylogenetic diversity* as the response variable.
**Table S9:** Summary of results, using *difference in gamma phylogenetic diversity* as the response variable.
**Table S10:** Difference in model performance when adding species status (native/non‐native).
**Table S11:** Difference in structural equation model performance when adding the traits.
**Table S12:** Relationship between traits and indigenous‐PD for all four PD metrics.

## Data Availability

All data and code supporting the manuscript are available in Zenodo. DOI: https://doi.org/10.5281/zenodo.20287667 (Brian 2026).
