# Supplementary material for: Plants That Evolved Under High Phylogenetic Diversity Have Higher Invasion Success, Particularly in Undisturbed Communities
Source: Ecol Lett. 2026 Jun 25;29(6):e70417. doi: 10.1111/ele.70417 (PMC13297893; doi:10.1111/ele.70417)
Supplement: Supplementary file 1 — Figure S1: Experimental design of Haeuser et al. 2017. Figure S2: Experimental design of Kempel et al. 2013. Figure S3: Experimental design of Müller et al. 2016. Figure S4: Calculating metrics of phylogenetic diversity (PD). Figure S5: The indigenous‐PD for a given species is not related to the proportion of tree species in its indigenous range (p = 0.429). Figure S6: Correlations between the phylogenetic diversity metrics used in the study. Figure S7: Histograms showing the climate dissimilarity (experimental climate minus native range climate) for all species in the experiments, where ‘count’ refers to the number of species in each climate bin. Figure S8: Structure of the two competing structural equation models (SEMs) tested for each model. Figure S9: Marginal effects plot showing interactions between maximum alpha PD and disturbance on metrics of seeded species success. Figure S10: Marginal effects plot showing interaction between median alpha PD, disturbance and climate dissimilarity on metrics of seeded species success. Figure S11: Marginal effects plot showing interaction between gamma PD, disturbance and temperature dissimilarity on metrics of seeded species success. Figure S12: Marginal effects plot showing interaction between disturbance and (a) mean, (b) maximum and (c) gamma PD on the likelihood of species surviving to the end of the second growing season. Figure S13: Marginal effects plot showing interaction between maximum alpha PD difference and climate dissimilarity or disturbance on plant responses at various time points (Haeuser data). Figure S14: Marginal effects plot showing interaction between median alpha PD and climate dissimilarity or disturbance on plant responses at various time points (Haeuser data). Figure S15: Marginal effects plot showing interaction between gamma alpha PD and climate dissimilarity or disturbance on plant responses at various time points (Haeuser data). Figure S16: Marginal effects plot showing interaction between clim [file ELE-29-0-s001.pdf]

# Plants that evolved under high phylogenetic diversity have higher invasion success, particularly in undisturbed communities: Supporting Information

**Joshua I. Brian, Mark van Kleunen, Wayne Dawson, Anne Kempel, Weihan Zhao, Jane A. Catford**


| | |
|---|---|
| Supplementary Methods | 2 |
| Phylogenetic tree construction | 2 |
| Climate data | 3 |
| Robustness tests | 3 |
| Species origin | 5 |
| Structural equation models for trait inclusion | 5 |
| Supplementary methods figures (Figs. S1 – S8) | 7 |
| Supplementary methods tables (Table S1) | 15 |
| Supplementary Results | 19 |
| Effect of absolute indigenous-PD (summary and Figs. S9 – S12) | 19 |
| Effect of relative indigenous-PD (summary and Figs. S13 – S16) | 24 |
| Effect of precipitation dissimilarity (Fig. S17) | 30 |
| Species origin and trait inclusion (summary) | 31 |
| Supplementary tables for effect of absolute indigenous-PD (Tables S2 – S5) | 32 |
| Supplementary tables for effect of relative indigenous-PD (Tables S6 – S9) | 40 |
| Supplementary tables for species origin and traits (Tables S10 – S12) | 50 |
| Supplementary References | 54 |

Supplementary Methods

*Phylogenetic tree construction*

We constructed a phylogenetic tree for the global extant flora following the process used in van Kleunen et al. (2020) and Yang et al. (2021). The basis for this tree was the mega phylogeny for seed plant species built by Smith and Brown (Smith & Brown 2018). We first harmonized the tip names of the mega phylogeny according to the World Checklist of Vascular Plants (WCVP) v.11 and pruned the tree to only include species with accepted names in the WCVP v.11. Tip names matched to synonyms were consolidated with corresponding accepted names. Because 26.7% of the accepted species names in WCVP v.11 (n = 105,749) were missing from the phylogenetic tree, we added them manually using the R package "phytools" (Revell 2024). Specifically, for infraspecific names with their conspecific names in the tree, we added them to the root of their species (n = 42,255, 10.65%). The remaining ones were added to the root of their genus (n = 62,020, 15.63%) or, if the whole genus was missing, to the family root (n = 1,474, 0.37%). We used this phylogenetic tree for the global species pool (all 396,709 seed plant species with accepted names in the WCVP v.11) for the main analysis. This phylogeny is available in Part 1 of the accompanying Zenodo repository ('wcvp.v11.tree.wcvp.id.sub.RData'). The Zenodo repository can be found at: https://doi.org/10.5281/zenodo.20287667

This phylogeny contains both woody and non-woody plants. The presence of woody plants (i.e. trees and large shrubs) could potentially bias our interpretation of indigenous-PD by including species that our focal grassland species do not interact with. We do not believe that this is concerning, as the aim of indigenous-PD is to capture the evolutionary conditions of our focal species. In evolutionary time, there is strong potential for woody plants to have affected grassland plants, even if there was not direct species-species interaction. For example, higher woody species diversification narrows the range of resources available in a given area for non-woody species, encouraging specialisation and competitive superiority in remaining niche space among those non-woody species (Stachowicz & Tilman 2005). Further, evidence from the geological record (e.g. in both Europe and Madagascar) suggests tree and grassland cover oscillated through time, implying interactions and shifts in competitive dominance between functional groups at habitat edges (Bond & Midgley 2012; Silander Jr. et al. 2024; Czyżewski et al. 2026). The evolution of grasses potentially occurred

in mosaic landscapes such as in forest gaps, and C3 grasses continue to be more commonly found in forest understory than in open grassland (Scholes & Archer 1997; Strömberg 2011). Finally, there is contemporary evidence from competition experiments that trees and grassland species continue to compete (e.g. Belsky 1994; Ludwig et al. 2004; Riginos 2009; Dohn et al. 2013). Therefore, we believe woody species are important to maintain in our analyses of indigenous-PD as they likely played a role in the evolution of our grassland species, whether through direct interactions (competition, facilitation) or indirectly through the constriction of available niche space for non-woody species.

Nevertheless, to test whether woody species affect overall indigenous-PD, we calculated what proportion of species in each species' native range were tree species. We did this by using the list of tree species and distributions from the GlobalTreeSearch database from Botanic Gardens Conservation International (BGCI 2026). We tested whether there was a significant relationship between the proportion of tree species in a species' native (i.e. home) range and mean alpha PD for each species. If there was a significant relationship, this would imply that species indigenous-PD is influenced by the diversity of tree species in species' native (home) ranges. However, there was no significant relationship ($F_{1, 161} = 0.629$, $p = 0.429$; Fig. S6). Therefore, calculations of indigenous-PD were not sensitive to tree species richness.

*Climate data*

As an alternative to the PCA of all bioclimatic variables, we also calculated temperature and precipitation dissimilarity using two bioclimatic variables directly: minimum temperature of the coldest month (bio6) and precipitation of the driest quarter (bio17). These two variables loaded most strongly on to PC1 and PC2, respectively, and are both considered key variables determining colonisation likelihood in central European plant communities (Dullinger et al. 2017). We used these two dissimilarities in alternate models to check robustness of key trends (see *Supplementary analyses: Robustness tests*).

*Robustness tests*

To test the robustness of our analyses, we tested sets of alternate models. We had three sets of alternate models for testing the influence of absolute indigenous-PD. First, as several trends were consistent across models testing both absolute and relative indigenous-PD, we repeated

analyses only using Kempel and Müller data (‘K+M’ models), to confirm that significant results in the absolute models were not being driven purely by Haeuser data. Second, we calculated a coarse version of ‘difference in PD’ (not abundance-weighted) between planted species and the resident community, using Haeuser and Kempel data, and used this metric as the key response variable (‘K+H’ models). This was possible as Kempel had semi-quantitative resident vegetation data at the site level and was used to check whether the absolute results were consistent for difference in PD rather than raw PD metrics. Third, we reran the main models using the single climate variables (‘SC’ models) rather than the dissimilarities from the principal components analysis.

We had two sets of alternate models for the testing the influence of relative indigenous-PD. First, we ran SC models as described above. Second, we ran all models using raw indigenous-PD metrics rather than difference in PD (‘Hnd’ models). Results for all alternate models generally supported the main results; all models are compared in Tables S2 – S9. For all alternate models, we ran them for all four indigenous-PD metrics (mean alpha PD, maximum alpha PD, median alpha PD, gamma PD).

To enable comparison of effect size coefficients, we carried out all analyses twice; first with unstandardised continuous variables, and second with standardised continuous variables (centred on the mean and divided by two standard deviations; Gelman 2008). We report both for transparency in the supplementary tables S2 – S9, with the standardised effect size in brackets. To fairly compare the effect of different variables (i.e. Figs. 2, 4 in main text), we use the standardised variables, but we maintain the unstandardised variables when interpreting the effect of indigenous-PD alone (i.e. Figs. 3, 5, 6 in main text) as the values have specific ecological meanings. Standardising the continuous variables did not alter p-values, except in very rare cases where two standardised variables were in an interaction. The p-value of the interaction did not change, only the p-values of the main effect coefficients for those variables. In these cases, we also report the standardised p-values in brackets in tables S2 – S9. As the main effect terms are not interpreted when the variables are in interactions, overall interpretations are identical between the standardised and non-standardised versions.

*Species origin*

To test whether our results held for both native and non-native sown species, we added species origin (native/non-native) to all our final selected models as a fixed effect and compared models with and without species origin using difference in AIC. Models within 2 AIC were considered equivalent. See Table S10 for results.

To test whether our results held for species from different biogeographic regions, we assigned species to their native 'biogeographic syndrome' following Fristoe et al. (2023). Briefly, biogeographic syndromes provide information on the combination of continents to which a species is native. For example, a species that is only native somewhere in Europe would have a different biogeographic syndrome to a species that is native to both Europe and Asia, etc. Overall, our 166 species showed 24 different biogeographic syndromes. We added species biogeographic syndrome to all our final selected models as a random effect and compared models with and without biogeographic origin using difference in AIC. Models within 2 AIC were considered equivalent. See Table S10 for results.

We also tested whether the PD-trait correlations were affected by biogeographic syndrome. We carried out two additional tests: first, we limited data to those species that were native to North America or Europe, to avoid potential biases introduced by tropical species that can have systematically different trait values (Moles et al. 2007) but are also systematically different in regional indigenous-PD (Fig. 1c, main text). Second, we used the full dataset but included biogeographic syndrome as a random variable. See Table S12 for results.

*Structural equation models for trait inclusion*

To investigate whether the effect of indigenous-PD was mediated entirely through plant traits, we ran two sets of structural equation models for the main models where indigenous-PD was found to be significant. In the first set, we limited indigenous-PD to have only indirect effects on the response variable (i.e. the effect of indigenous-PD is mediated entirely through plant traits), while in the second set we allowed both direct and indirect effects of indigenous-PD (Fig. S8). Models were identical to the main text models aside from the addition of traits (i.e. all other covariates always remained in the models), as the goal was to verify whether the

main text model results were robust to the inclusion of traits. The model sets were compared using AIC, with a lower AIC indicating a superior model.

We chose three traits for inclusion: specific leaf area (SLA), vegetative height, and seed mass. These three traits all load strongly on to the aboveground spectrum of plant form and function (Díaz et al. 2016; Carmona et al. 2021) and are the three traits most measured for naturalised plant species (Grenié et al. 2025). They were also the three traits for which we had the most data for our 166 species (Table S11). After exploring all data sources, we had trait data for 153 of our 166 species (seed mass), 149 species (vegetative height) and 133 species (SLA). We chose to limit our analyses to species that had trait data, rather than imputing missing values, for two reasons. First, while imputation is useful for community-level trends and multivariate analyses, it is less appropriate when specific data points that were created by imputation would be used in downstream analysis, as imputation methods are not accurate enough to produce reliable species-level data (Gorné et al. 2025). Second, imputation requires the incorporation of phylogenetic information. However, indigenous-PD has a phylogenetic signal - in other words, more closely related species are more likely to have more similar indigenous-PD values ($C_{mean} = 0.157$, $p = 0.002$; Moran's $I = 0.063$, $p = 0.006$). Therefore, imputation will generate circularity: if we use phylogenetic information to help impute missing trait values, and then ask if the effect of indigenous-PD on success is mediated through traits, we are more likely to find a positive result, because we are contributing to a phylogenetic signal in the traits that is independently seen in indigenous-PD.

For each set of structural equation models, we tested two trait combinations. First, we tested the effect of SLA and height together (N = 131 species for both traits), as representatives of the two axes of aboveground form and function (Carmona et al. 2021). Second, we tested the effect of SLA, height and seed mass together (N = 121 species for all three traits), to incorporate reproductive investment.

Models were fit using the piecewiseSEM R package (Lefcheck 2016). See Table S11 for results.

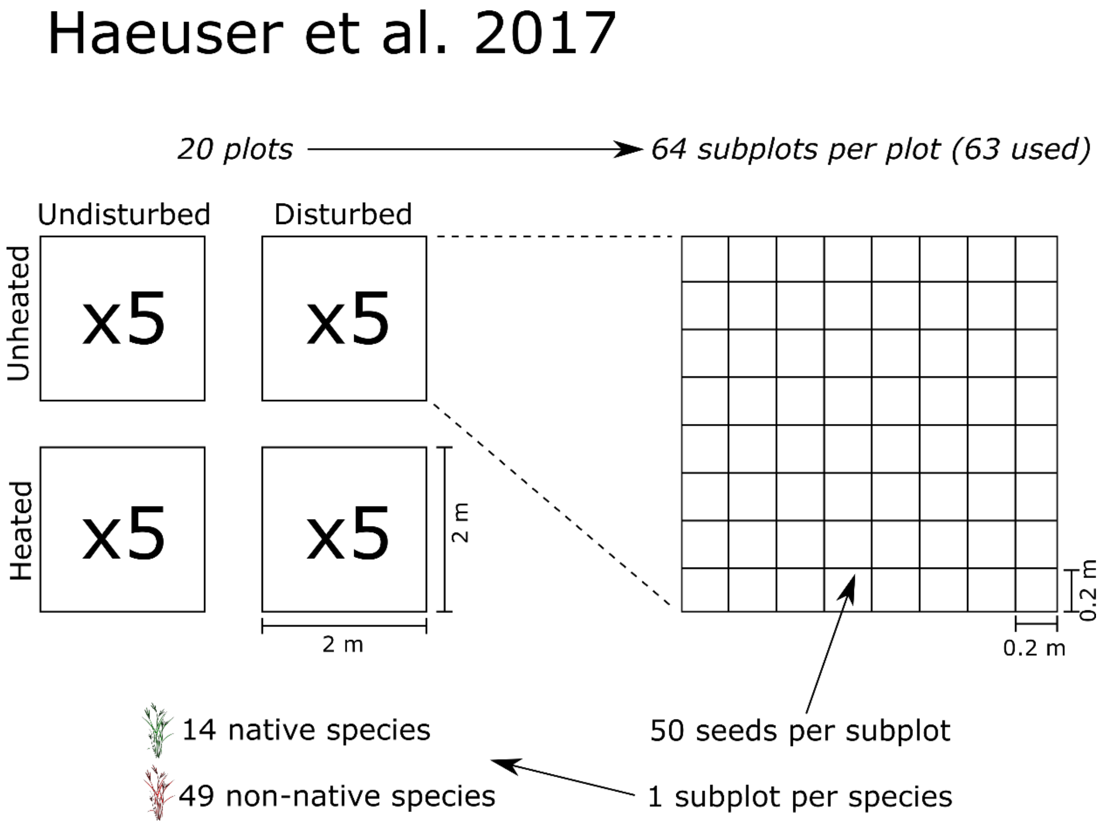


**Figure S1**: Experimental design of Haeuser et al. 2017. Replicates of each treatment combination were arranged within a single site at the University of Konstanz, Germany.

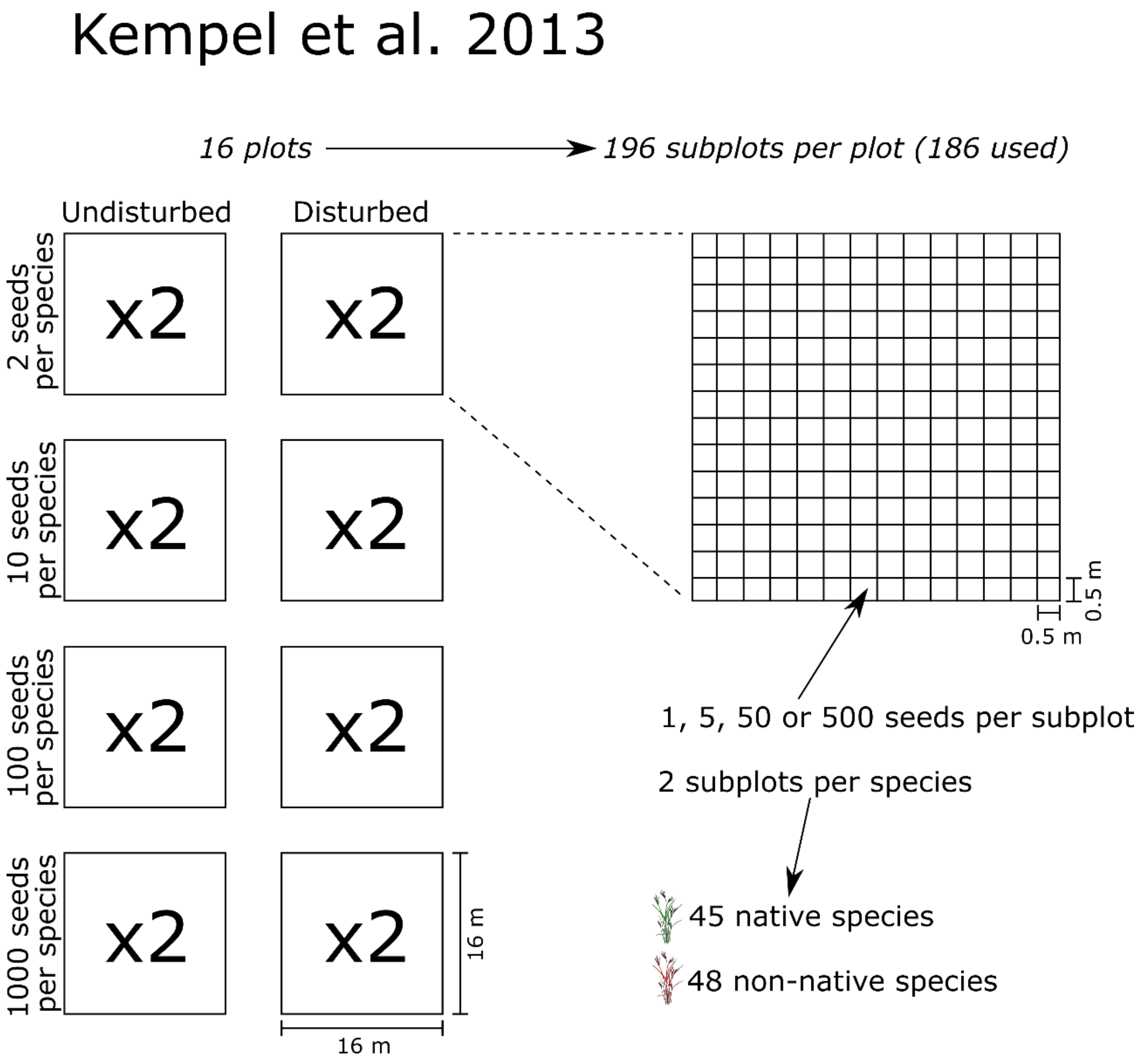


**Figure S2**: Experimental design of Kempel et al. 2013. Replicates of each treatment combination were arranged across 16 different sites in the northern half of the canton of Bern (Switzerland). Sites were a maximum of 50 km from each other.

# Müller et al. 2016

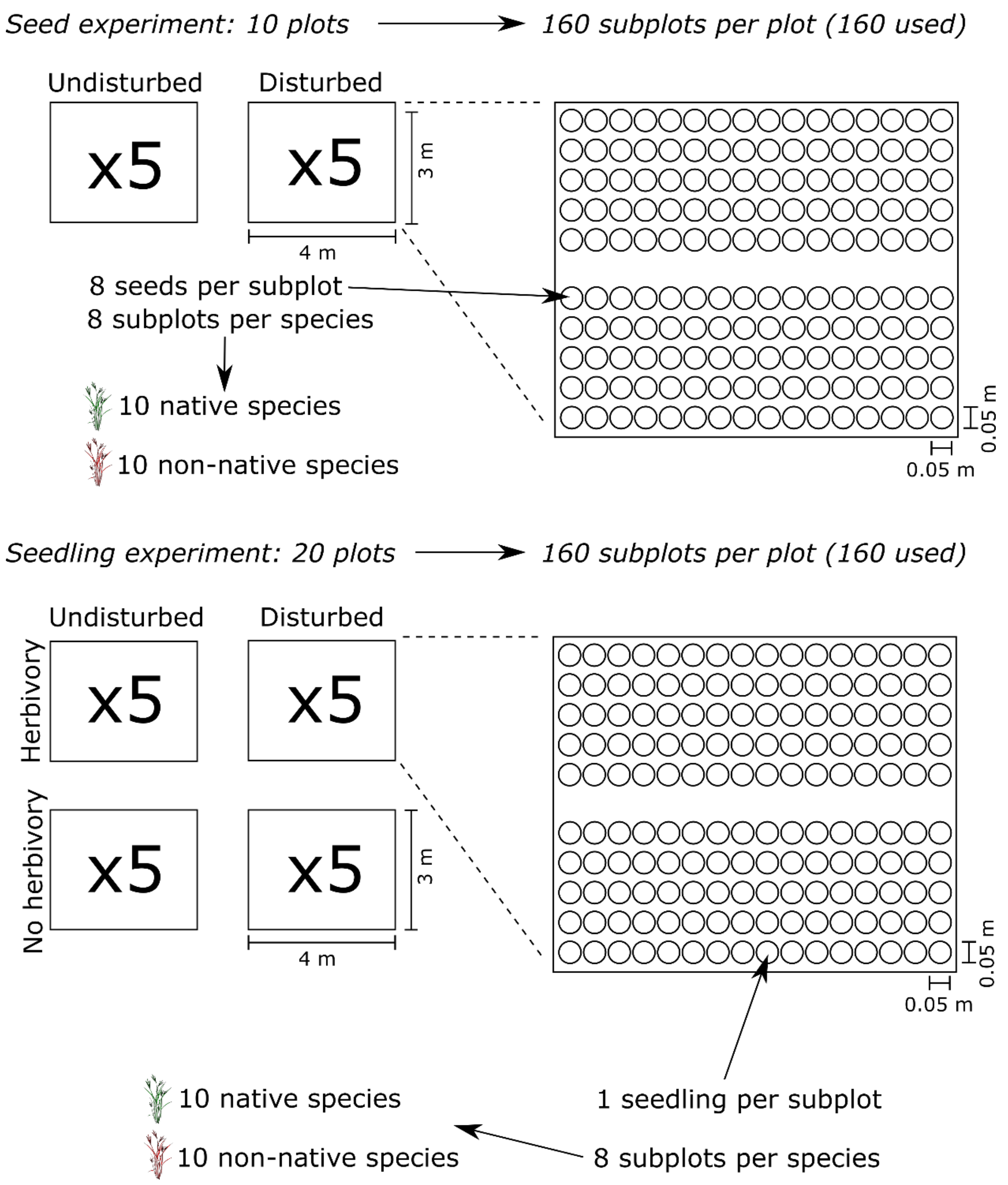


**Figure S3**: Experimental design of Müller et al. 2016. Replicates of each treatment combination were arranged across five sites adjacent to the University of Konstanz, Germany. Note that both Müller experiments also featured a belowground biocide treatment; only control plots with respect to this variable are used in the present study. The experimental species were identical between the seed and seedling experiments, with one exception in the native species. Therefore, while each experiment used 20 species, in total 21 species were used (11 native, 10 non-native).

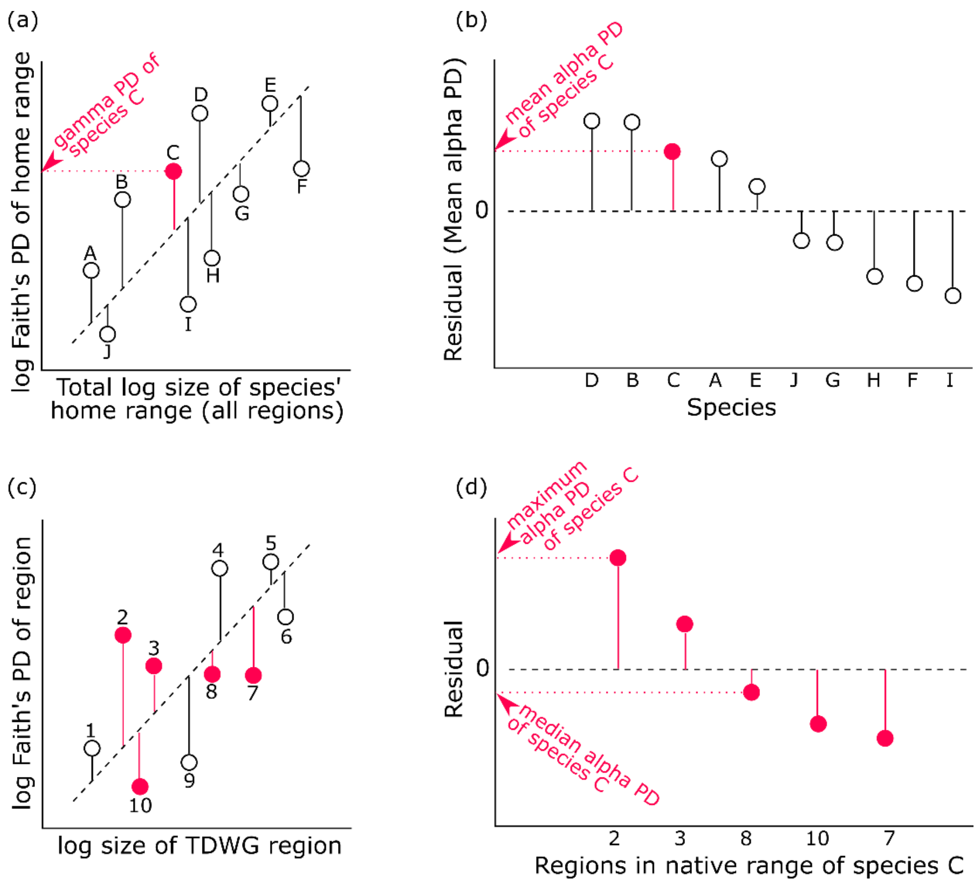


**Figure S4**: Calculating metrics of phylogenetic diversity (PD). (a) Gamma PD is defined as the total PD in the indigenous range of a species, across all TDWG3 regions. Here, ten species are plotted (species A – species J), with the total phylogenetic diversity in the native range of species C highlighted (the gamma PD of species C). (b) Mean alpha PD (the focal metric in the study) is defined as the mean PD across the indigenous range of a species. It is calculated as the residual of the log-log relationship between native range size and gamma PD (species C highlighted as an illustration). (c) Individual TDWG3 regions are plotted and regressed against the PD within each region, with ten regions shown as an example. Species C is native to five of those regions (highlighted). (d) Maximum alpha PD is defined as the maximum area-corrected PD of a region in which a species is indigenous (the highest residual of all the regions where it is native), while median alpha PD is defined as the median area-corrected PD of a region in which a species is indigenous. Calculation of mean alpha, maximum alpha and gamma PD were as described in Fristoe et al. (2023).

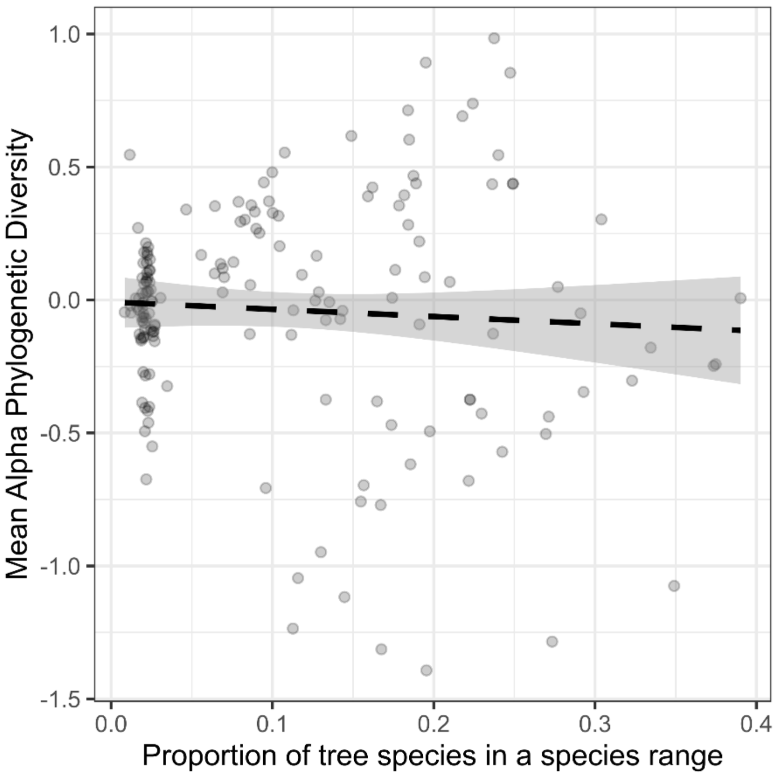


**Figure S5**: The indigenous-PD for a given species is not related to the proportion of tree species in its indigenous range (p = 0.429). Each dot is a species. Note that the 95% confidence interval (grey shaded area) overlaps zero for the whole range of the x-axis suggesting that, for any given tree proportion, all species with that proportion in their indigenous range still have a collective mean indigenous-PD of 0, as predicted by the null hypothesis. Collectively, this strongly suggests that tree species do not bias calculations of indigenous-PD. The large collection of points at approximately 0.025 on the x-axis is species of European origin.

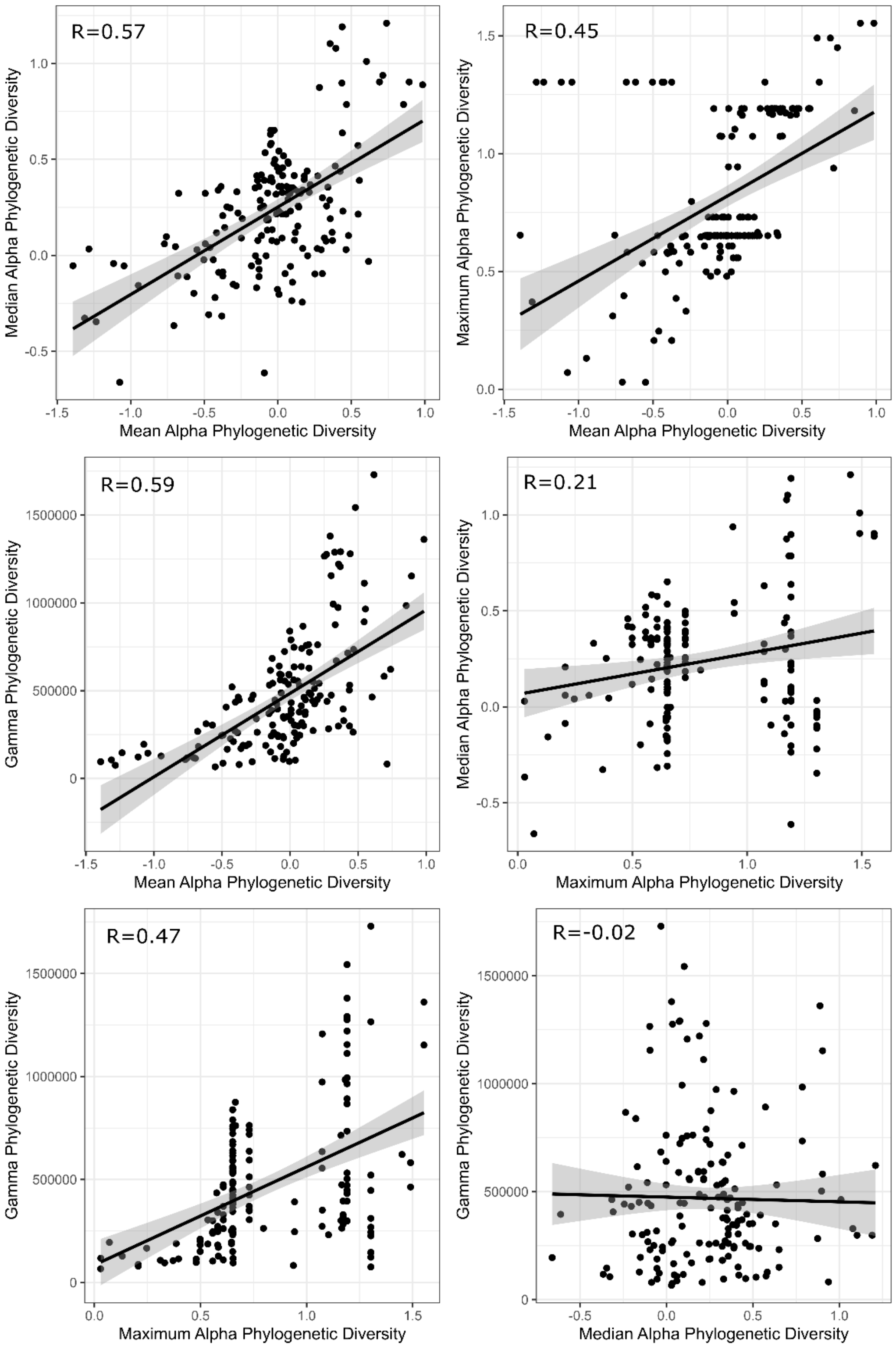


**Figure S6**: Correlations between the phylogenetic diversity metrics used in the study. Inset values indicate Pearson's R. Each point is a species.

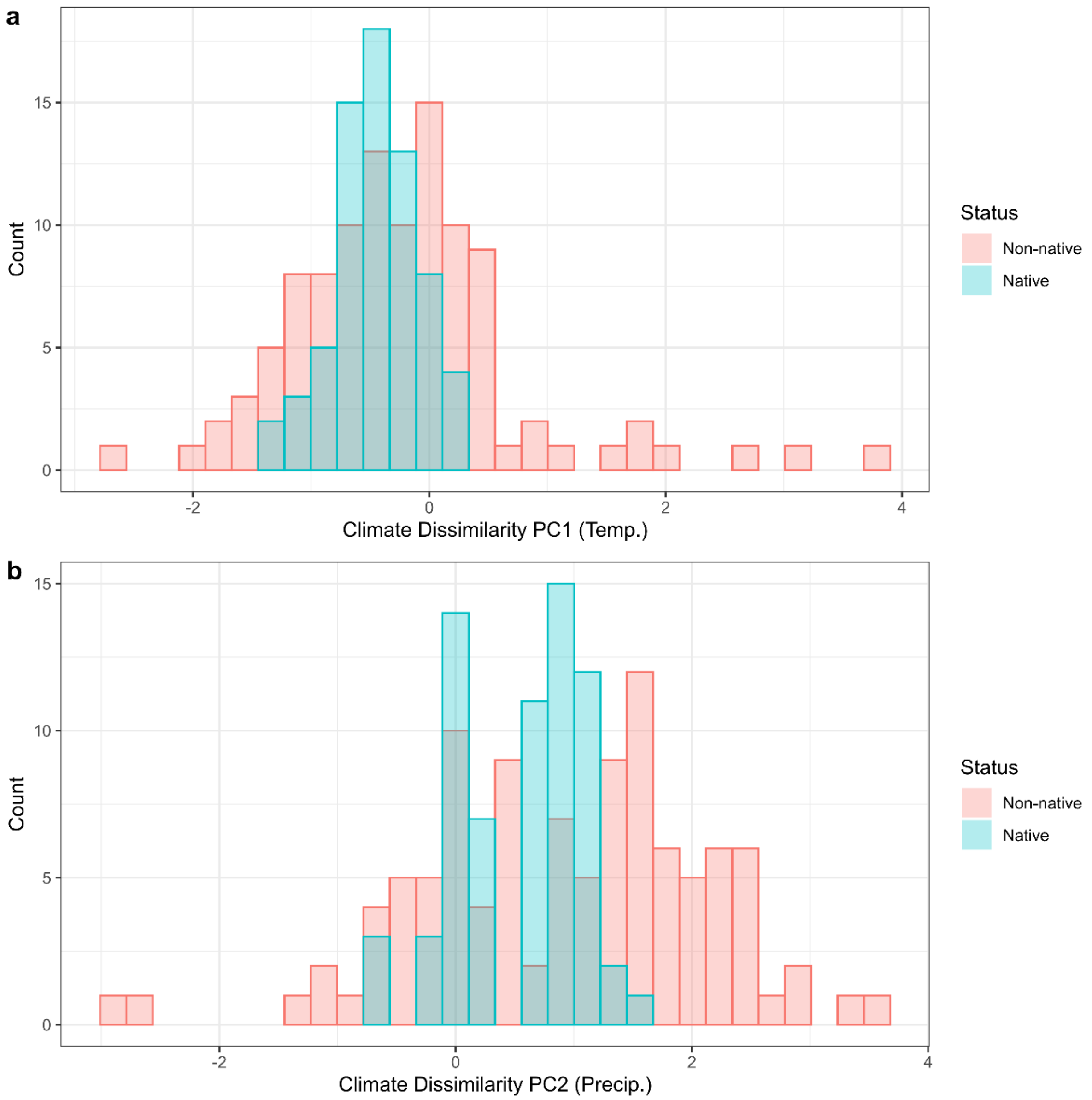


**Figure S7**: Histograms showing the climate dissimilarity (experimental climate minus native range climate) for all species in the experiments, where 'count' refers to the number of species in each climate bin. Species are divided by native status (native or non-native). Non-native sown species had higher dissimilarities than native sown species (temperature dissimilarity, $\chi^2_1 = 7.296$, p=0.007; precipitation dissimilarity, $\chi^2_1 = 21.945$, p<0.001), and native species had dissimilarities more closely centred on zero.

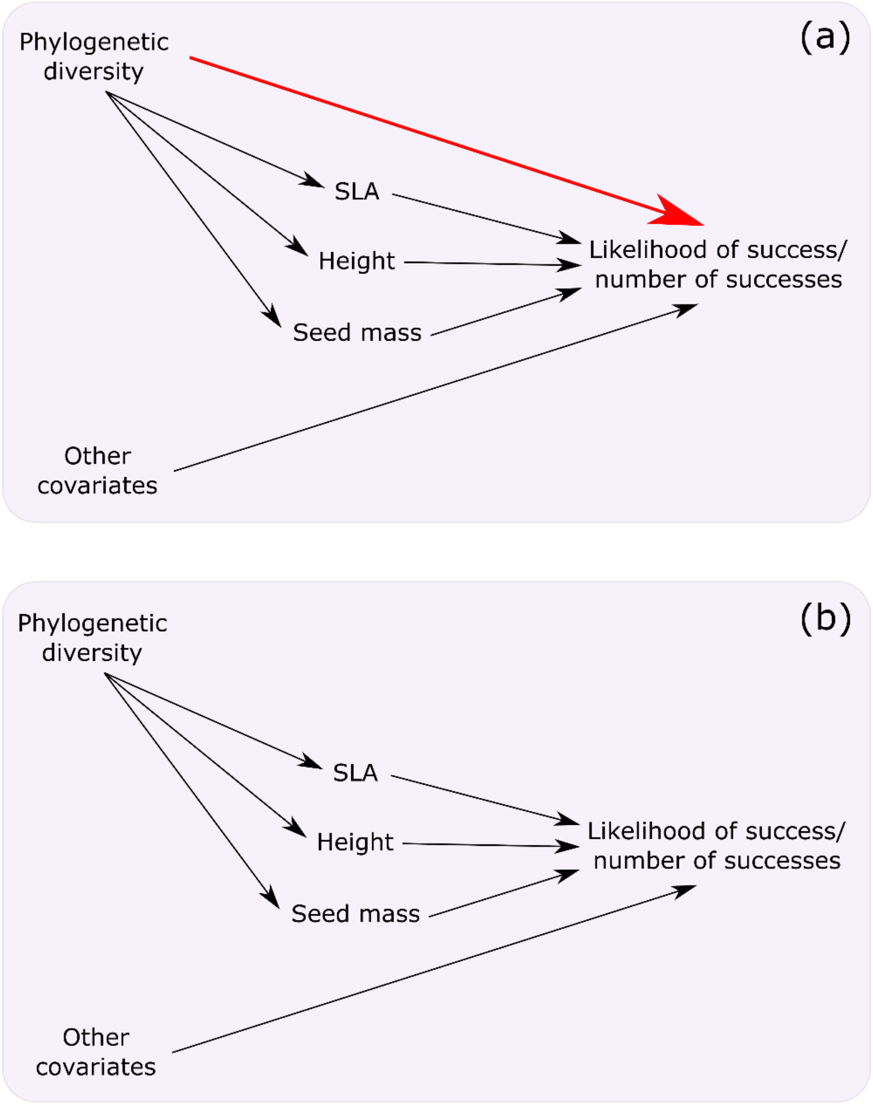


**Figure S8**: Structure of the two competing structural equation models (SEMs) tested for each model where indigenous phylogenetic diversity (indigenous-PD) was found to be significant. (a) SEM testing both direct and indirect effects of indigenous-PD on the likelihood of planted species success and the number of successes. (b) SEM testing indirect effects only of indigenous-PD on species success, whereby the effect of indigenous-PD is mediated entirely through species traits. Models (a) and (b) were compared with AIC with the lower AIC being preferred. The link being tested by this approach is highlighted in red; if model (a) has a lower AIC this indicates that the PD has a direct effect on species success in the experiment, independent of the tested traits. 'Other covariates' includes all other variables included in the main text models to ensure consistency in interpretation.

*Supplementary methods tables*

Table S1: Complete list of all species across all three experiments. 'Species' uses the Plants of the World (POWO) accepted name. 'ID' is the identification number in the World Checklist of Vascular Plants (WCVP) database. The three study columns indicate whether a species was sown (1) or not sown (0) in that study. 'Status' indicates whether the species was native or non-native to the sown location.

| **Species** | **ID** | **Müller** | **Haeuser** | **Kempel** | **Status** |
|---|---|---|---|---|---|
| *Achillea filipendulina* | 3109923 | 0 | 1 | 1 | Exotic |
| *Achillea millefolium* | 2908230 | 1 | 1 | 0 | Native |
| *Achillea nobilis* | 3110044 | 1 | 0 | 0 | Native |
| *Aconitum carmichaelii* | 2618501 | 0 | 1 | 0 | Exotic |
| *Ajuga reptans* | 5385 | 0 | 1 | 1 | Native |
| *Alcea rosea* | 2627710 | 0 | 0 | 1 | Exotic |
| *Allium schoenoprasum* | 296525 | 0 | 1 | 0 | Native |
| *Alyssum alyssoides* | 2631899 | 0 | 0 | 1 | Native |
| *Amaranthus tricolor* | 2633132 | 0 | 1 | 0 | Exotic |
| *Anchusa arvensis* | 2636464 | 0 | 0 | 1 | Native |
| *Anchusa capensis* | 2636491 | 0 | 0 | 1 | Exotic |
| *Anchusa officinalis* | 2636606 | 0 | 0 | 1 | Native |
| *Anoda cristata* | 2641086 | 0 | 0 | 1 | Exotic |
| *Antirrhinum majus* | 2642724 | 0 | 1 | 0 | Exotic |
| *Aquilegia viridiflora* | 2645036 | 0 | 0 | 1 | Exotic |
| *Aquilegia vulgaris* | 2645045 | 0 | 1 | 1 | Native |
| *Arabis hirsuta* | 2645681 | 0 | 0 | 1 | Native |
| *Arabis caucasica* | 2645447 | 0 | 0 | 1 | Exotic |
| *Aster amellus* | 3099441 | 1 | 0 | 0 | Native |
| *Aster bellidiastrum* | 3099482 | 0 | 0 | 1 | Native |
| *Atropa bella-donna* | 2665943 | 0 | 1 | 0 | Native |
| *Aurinia saxatilis* | 2666583 | 0 | 0 | 1 | Exotic |
| *Borago officinalis* | 2679695 | 0 | 0 | 1 | Native |
| *Bunias orientalis* | 2686094 | 0 | 0 | 1 | Exotic |
| *Calendula officinalis* | 2910167 | 0 | 0 | 1 | Exotic |
| *Calystegia sepium* | 483559 | 0 | 0 | 1 | Native |
| *Campanula armena* | 363990 | 0 | 0 | 1 | Exotic |
| *Campanula barbata* | 364044 | 0 | 0 | 1 | Native |
| *Campanula pyramidalis* | 365752 | 0 | 0 | 1 | Exotic |
| *Campanula rapunculus* | 365813 | 0 | 0 | 1 | Native |
| *Campanula rotundifolia* | 365918 | 0 | 0 | 1 | Native |
| *Cardamine pratensis* | 2700274 | 1 | 0 | 1 | Native |
| *Cenchrus caudatus* | 487074 | 0 | 1 | 0 | Exotic |
| *Centaurea macrocephala* | 3050068 | 0 | 1 | 0 | Exotic |
| *Cerastium tomentosum* | 2710954 | 1 | 1 | 0 | Exotic |
| *Chelidonium majus* | 2716702 | 0 | 0 | 1 | Native |
| *Cichorium intybus* | 2901597 | 0 | 0 | 1 | Native |
| *Circaea lutetiana* | 2721837 | 0 | 0 | 1 | Native |

| | | | | | |
|---|---|---|---|---|---|
| *Clarkia amoena* | 2724644 | 0 | 0 | 1 | Exotic |
| *Clematis terniflora* | 2726804 | 0 | 0 | 1 | Exotic |
| *Clematis vitalba* | 2726964 | 0 | 0 | 1 | Native |
| *Convolvulus arvensis* | 483761 | 0 | 0 | 1 | Native |
| *Convolvulus tricolor* | 484499 | 0 | 0 | 1 | Exotic |
| *Cuminum cyminum* | 2747364 | 0 | 1 | 0 | Exotic |
| *Cynoglossum amabile* | 2751851 | 0 | 0 | 1 | Exotic |
| *Cynoglossum officinale* | 2752089 | 0 | 0 | 1 | Native |
| *Datura stramonium* | 2757848 | 0 | 0 | 1 | Exotic |
| *Delphinium ajacis* | 2759384 | 0 | 1 | 0 | Exotic |
| *Dianthus armeria* | 2763820 | 0 | 0 | 1 | Native |
| *Dianthus caryophyllus* | 2764005 | 0 | 0 | 1 | Exotic |
| *Digitalis lanata subsp. trojana* | 3008772 | 0 | 1 | 0 | Exotic |
| *Diplotaxis tenuifolia* | 2771347 | 1 | 0 | 0 | Exotic |
| *Echium vulgare* | 2784423 | 0 | 0 | 1 | Native |
| *Epilobium dodonaei* | 2790362 | 1 | 0 | 1 | Native |
| *Epilobium tetragonum* | 2791119 | 1 | 0 | 0 | Native |
| *Eragrostis trichodes* | 413314 | 0 | 1 | 0 | Exotic |
| *Eranthis hyemalis* | 2791911 | 0 | 1 | 0 | Exotic |
| *Erigeron acris* | 3104709 | 0 | 0 | 1 | Native |
| *Eritrichium canum* | 2797689 | 0 | 1 | 0 | Exotic |
| *Eschscholzia californica* | 2801972 | 0 | 0 | 1 | Exotic |
| *Eudianthe coeli-rosa* | 2802809 | 0 | 0 | 1 | Exotic |
| *Eupatorium cannabinum* | 2926599 | 1 | 0 | 0 | Native |
| *Euthamia graminifolia* | 3102167 | 1 | 0 | 0 | Exotic |
| *Galeopsis angustifolia* | 85331 | 0 | 0 | 1 | Native |
| *Gilia tricolor* | 2825947 | 0 | 1 | 0 | Exotic |
| *Gypsophila elegans* | 2836649 | 0 | 0 | 1 | Exotic |
| *Gypsophila paniculata* | 2836785 | 1 | 0 | 0 | Exotic |
| *Helenium bigelovii* | 3115764 | 0 | 1 | 0 | Exotic |
| *Helianthus annuus* | 2910966 | 0 | 0 | 1 | Exotic |
| *Helianthus debilis* | 3120900 | 0 | 1 | 0 | Exotic |
| *Heliotropium arborescens* | 2843787 | 0 | 1 | 0 | Exotic |
| *Hesperis matronalis* | 2847567 | 0 | 1 | 0 | Exotic |
| *Hibiscus trionum* | 2850654 | 0 | 0 | 1 | Exotic |
| *Hordeum jubatum* | 419358 | 0 | 1 | 0 | Exotic |
| *Iberis amara* | 2859697 | 0 | 0 | 1 | Native |
| *Iberis sempervirens* | 2859919 | 0 | 0 | 1 | Exotic |
| *Ipomoea tricolor* | 482807 | 0 | 0 | 1 | Exotic |
| *Iris domestica* | 334601 | 0 | 1 | 0 | Exotic |
| *Iris sibirica* | 322377 | 0 | 1 | 0 | Native |
| *Ismelia carinata* | 3112289 | 0 | 0 | 1 | Exotic |
| *Jacobaea maritima subsp. bicolor* | 3082659 | 0 | 0 | 1 | Exotic |
| *Jacobaea vulgaris* | 3082777 | 1 | 0 | 0 | Native |
| *Knautia arvensis* | 2334543 | 0 | 0 | 1 | Native |
| *Lathyrus odoratus* | 2351328 | 0 | 0 | 1 | Exotic |

| | | | | | |
|---|---|---|---|---|---|
| *Legousia speculum-veneris* | 358587 | 0 | 0 | 1 | Native |
| *Lepidium graminifolium* | 2337784 | 1 | 0 | 0 | Native |
| *Lepidium heterophyllum* | 2337794 | 1 | 0 | 0 | Exotic |
| *Leucanthemum vulgare* | 3112766 | 0 | 0 | 1 | Native |
| *Lilium formosanum* | 293214 | 0 | 1 | 0 | Exotic |
| *Lilium regale* | 279843 | 0 | 1 | 0 | Exotic |
| *Lithotoma axillaris* | 494315 | 0 | 1 | 0 | Exotic |
| *Lobelia erinus* | 353498 | 0 | 0 | 1 | Exotic |
| *Lobelia inflata* | 353345 | 0 | 1 | 0 | Exotic |
| *Lobularia maritima* | 2338457 | 0 | 0 | 1 | Exotic |
| *Lotus corniculatus* | 2345329 | 0 | 1 | 0 | Exotic |
| *Lupinus ehrenbergii var. ehrenbergii* | 3017330 | 0 | 0 | 1 | Exotic |
| *Lysimachia clethroides* | 2493128 | 0 | 1 | 0 | Exotic |
| *Lysimachia punctata* | 2492220 | 0 | 1 | 0 | Exotic |
| *Lysimachia vulgaris* | 2492070 | 0 | 1 | 0 | Native |
| *Malva alcea* | 2503573 | 0 | 0 | 1 | Native |
| *Malva moschata* | 2503483 | 0 | 0 | 1 | Native |
| *Malva neglecta* | 2503504 | 0 | 0 | 1 | Native |
| *Malva trimestris* | 2504047 | 0 | 0 | 1 | Exotic |
| *Medicago lupulina* | 2368448 | 0 | 0 | 1 | Native |
| *Mentha spicata* | 125402 | 0 | 1 | 0 | Exotic |
| *Monarda fistulosa* | 129185 | 0 | 1 | 0 | Exotic |
| *Monarda punctata* | 129232 | 0 | 1 | 0 | Exotic |
| *Nemophila maculata* | 2379420 | 0 | 1 | 0 | Exotic |
| *Nepeta racemosa* | 134920 | 0 | 1 | 0 | Exotic |
| *Nicotiana mutabilis* | 2382251 | 0 | 1 | 0 | Exotic |
| *Nicotiana sylvestris* | 2385612 | 0 | 1 | 1 | Exotic |
| *Nigella arvensis* | 2381639 | 0 | 0 | 1 | Native |
| *Oenothera biennis* | 2399189 | 1 | 0 | 0 | Exotic |
| *Oenothera glazioviana* | 2398876 | 1 | 0 | 1 | Exotic |
| *Oenothera macrocarpa* | 2398993 | 0 | 0 | 1 | Exotic |
| *Origanum vulgare* | 143954 | 0 | 1 | 0 | Native |
| *Papaver betonicifolium* | 2502572 | 0 | 0 | 1 | Exotic |
| *Papaver cambricum* | 2560601 | 0 | 0 | 1 | Exotic |
| *Papaver dubium* | 2560793 | 0 | 0 | 1 | Native |
| *Papaver orientale* | 2561370 | 0 | 0 | 1 | Exotic |
| *Papaver rhoeas* | 2561554 | 0 | 0 | 1 | Native |
| *Papaver commutatum* | 2560637 | 0 | 0 | 1 | Exotic |
| *Persicaria capitata* | 2576526 | 0 | 1 | 0 | Exotic |
| *Persicaria virginiana* | 2572324 | 0 | 1 | 0 | Exotic |
| *Petunia integrifolia* | 2549707 | 0 | 1 | 0 | Exotic |
| *Phaseolus coccineus* | 2537776 | 0 | 0 | 1 | Exotic |
| *Phleum pratense* | 433846 | 0 | 1 | 0 | Native |
| *Phlox drummondii* | 2567292 | 0 | 0 | 1 | Exotic |
| *Physalis peruviana* | 2549655 | 0 | 0 | 1 | Exotic |
| *Phyteuma orbiculare* | 355252 | 0 | 0 | 1 | Native |

| | | | | | |
|---|---|---|---|---|---|
| *Plantago media* | 2569797 | 0 | 1 | 0 | Native |
| *Platycodon grandiflorus* | 354641 | 0 | 1 | 1 | Exotic |
| *Plectocephalus americanus* | 3046533 | 0 | 1 | 0 | Exotic |
| *Polemonium caeruleum* | 2566071 | 0 | 0 | 1 | Native |
| *Potentilla atrosanguinea* | 2953596 | 0 | 1 | 0 | Exotic |
| *Pseudo-fumaria lutea* | 2561323 | 0 | 1 | 0 | Exotic |
| *Rudbeckia fulgida* | 2928986 | 0 | 1 | 0 | Exotic |
| *Rudbeckia triloba* | 3123672 | 0 | 1 | 0 | Exotic |
| *Rumex crispus* | 2424294 | 0 | 1 | 0 | Native |
| *Salpiglossis sinuata* | 2579347 | 0 | 1 | 0 | Exotic |
| *Salvia argentea* | 182005 | 0 | 0 | 1 | Exotic |
| *Salvia farinacea* | 182630 | 0 | 0 | 1 | Exotic |
| *Salvia glutinosa* | 182731 | 0 | 0 | 1 | Native |
| *Salvia lyrata* | 183102 | 0 | 0 | 1 | Exotic |
| *Salvia verticillata* | 184133 | 0 | 1 | 0 | Exotic |
| *Saponaria officinalis* | 2484914 | 0 | 0 | 1 | Native |
| *Scabiosa columbaria* | 2593679 | 0 | 0 | 1 | Native |
| *Senecio ovatus* | 3082007 | 0 | 0 | 1 | Native |
| *Silene chalcedonica* | 2485408 | 0 | 0 | 1 | Exotic |
| *Silene flos-cuculi* | 2485564 | 0 | 0 | 1 | Native |
| *Silene latifolia* | 2488689 | 1 | 1 | 0 | Native |
| *Silene viscosa* | 2487795 | 1 | 0 | 0 | Native |
| *Solanum dulcamara* | 2932815 | 0 | 0 | 1 | Native |
| *Solanum nigrum* | 2915955 | 0 | 0 | 1 | Native |
| *Solidago canadensis* | 2911023 | 1 | 1 | 0 | Exotic |
| *Solidago ptarmicoides* | 3101706 | 0 | 1 | 0 | Exotic |
| *Symphyotrichum lanceolatum* | 3103441 | 1 | 0 | 0 | Exotic |
| *Symphyotrichum novi-belgii* | 3103597 | 1 | 0 | 0 | Exotic |
| *Thymus × citriodorus* | 204618 | 0 | 0 | 1 | Exotic |
| *Thymus pulegioides* | 205310 | 0 | 0 | 1 | Native |
| *Verbena rigida* | 212652 | 0 | 1 | 0 | Exotic |
| *Vicia sepium* | 2451888 | 0 | 1 | 0 | Native |
| *Viola odorata* | 2459312 | 0 | 1 | 0 | Exotic |
| *Viola tricolor* | 2461201 | 0 | 1 | 0 | Native |
| *Zinnia angustifolia* | 3124687 | 0 | 0 | 1 | Exotic |
| *Zinnia peruviana* | 3124740 | 0 | 1 | 0 | Exotic |

Supplementary Results

In all cases, models are discussed with reference to the models presented in the main text (i.e. do the additional models support or refute the models using mean alpha PD).

*Effect of absolute indigenous-PD*

The PD*disturbance interaction was consistent across colonisation and first year survival when using both maximum and median alpha PD (Figs. S9a-c; S10a-c); and was also seen in the second growing season for gamma PD (Fig. S11b). The PD*disturbance interaction was maintained regardless of whether the 'K+M', 'K+H' or 'SC' models were considered (Tables S2-S4), with occasional exceptions in the gamma PD models (Table S5). The PD*disturbance interaction in the second growing season was also seen in the 'SC' models for mean, maximum and gamma PD (Fig. S12).

The positive interaction between mean alpha PD and temperature dissimilarity in predicting second year survival was also seen when using median alpha PD and gamma PD (Figs. S10f, g; S11c). Interestingly, the trend was reversed for first year survival (Figs. S10e; S11a), suggesting that species initially require a level of ecological similarity to the resident community to survive and benefit from higher indigenous-PD in the first growing season, but for species that reach this 'minimum criteria', they are more likely to then succeed and benefit from high PD in the second growing season if they are ecologically different. However, note these effects were generally not seen in the 'SC' models (Tables S2, S4, S5). There were also occasional interactions with precipitation dissimilarity for both maximum alpha PD (Fig. S9d) and median alpha PD (Fig. S10d) in the second growing season. Having a higher indigenous-PD increased the *likelihood of any plants surviving* if precipitation dissimilarity was *low* (Fig. S9d), but having a higher indigenous-PD increased the *number of plants surviving* if precipitation dissimilarity was *high* (Fig. S10d). Though these interactions were only occasionally observed, this provides further evidence for the same interpretation: for a species to be able to survive and benefit from high indigenous-PD (increased likelihood) it needs to be ecologically similar, but for those species that can survive, they derive the greatest benefit (increased numbers) from being ecologically different.

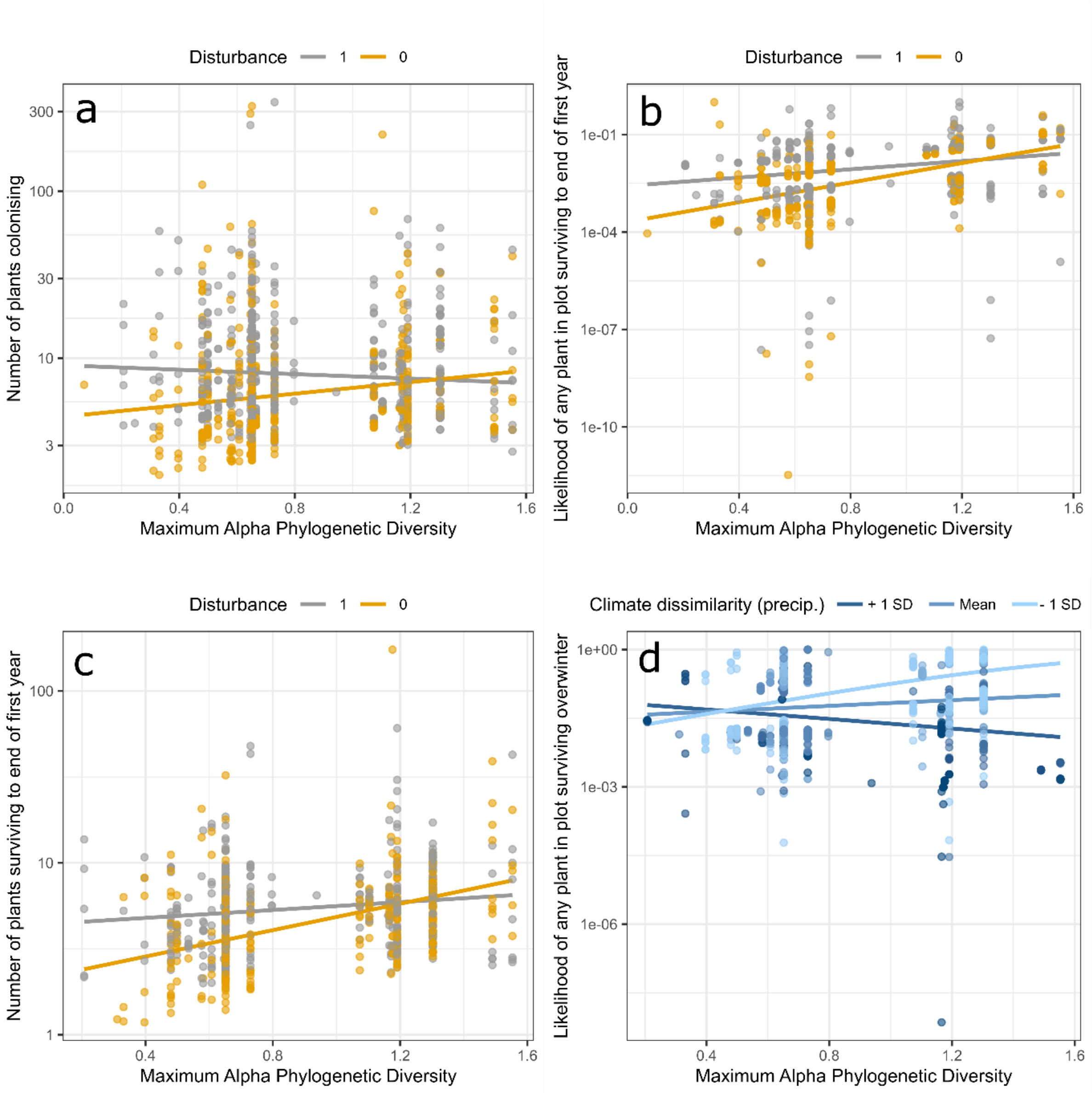


**Figure S9**: Marginal effects plot showing interactions between maximum alpha PD and disturbance on metrics of seeded species success. Plotted points represent observed data with all other fixed and random effects accounted for.

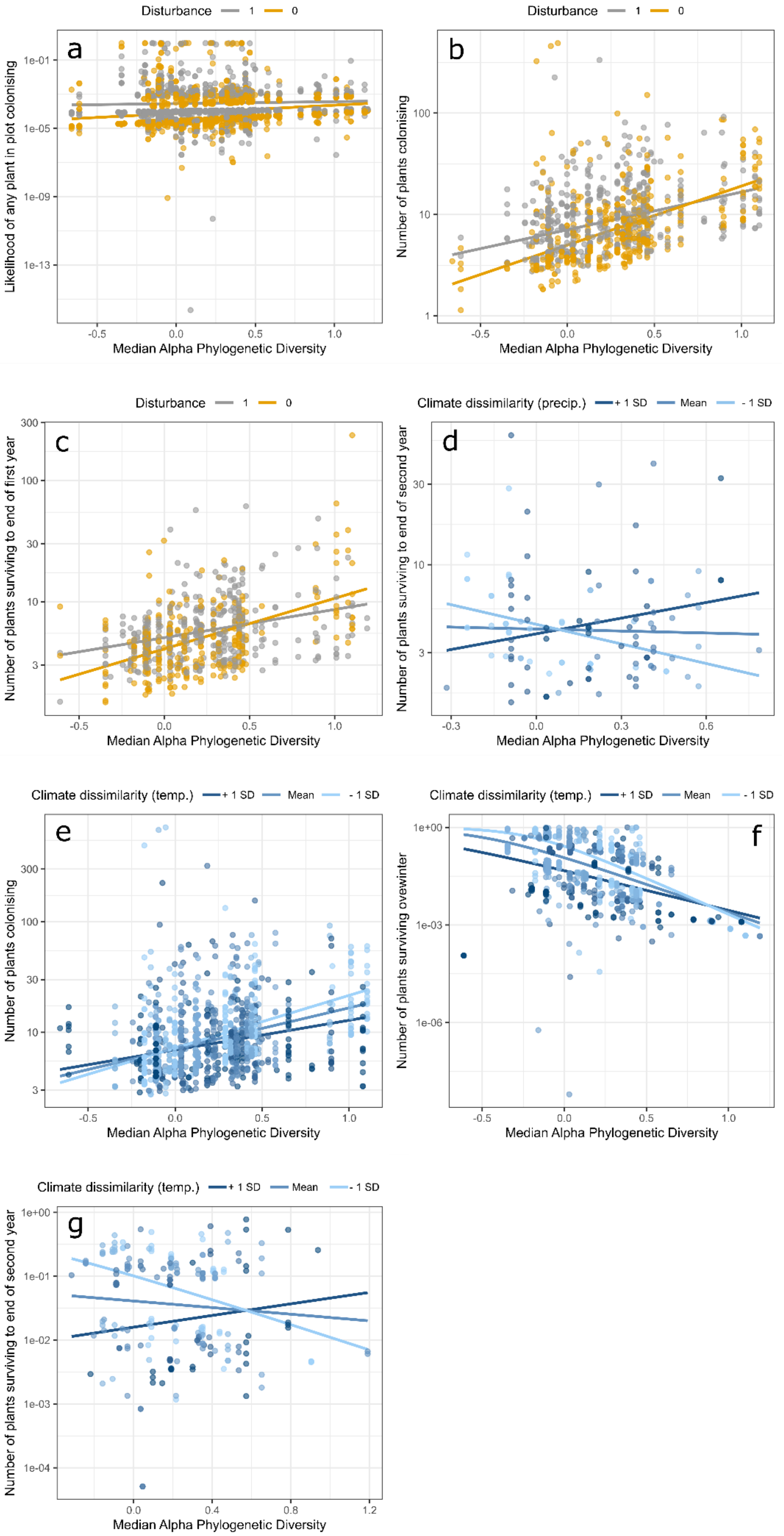

Disturbance 1 0
a
Likelihood of any plant in plot colonising
1e-01
1e-05
1e-09
1e-13
-0.5 0.0 0.5 1.0
Median Alpha Phylogenetic Diversity
Disturbance 1 0
b
Number of plants colonising
100
10
1
-0.5 0.0 0.5 1.0
Median Alpha Phylogenetic Diversity
Disturbance 1 0
c
Number of plants surviving to end of first year
300
100
30
10
3
-0.5 0.0 0.5 1.0
Median Alpha Phylogenetic Diversity
Climate dissimilarity (precip.) + 1 SD Mean - 1 SD
d
Number of plants surviving to end of second year
30
10
3
-0.3 0.0 0.3 0.6
Median Alpha Phylogenetic Diversity
Climate dissimilarity (temp.) + 1 SD Mean - 1 SD
e
Number of plants colonising
300
100
30
10
3
-0.5 0.0 0.5 1.0
Median Alpha Phylogenetic Diversity
Climate dissimilarity (temp.) + 1 SD Mean - 1 SD
f
Number of plants surviving overwinter
1e+00
1e-03
1e-06
-0.5 0.0 0.5 1.0
Median Alpha Phylogenetic Diversity
Climate dissimilarity (temp.) + 1 SD Mean - 1 SD
g
Number of plants surviving to end of second year
1e+00
1e-01
1e-02
1e-03
1e-04
0.0 0.4 0.8 1.2
Median Alpha Phylogenetic Diversity

**Figure S10 (previous page)**: Marginal effects plot showing interaction between median alpha PD, disturbance and climate dissimilarity on metrics of seeded species success. Plotted points represent observed data with all other fixed and random effects accounted for.

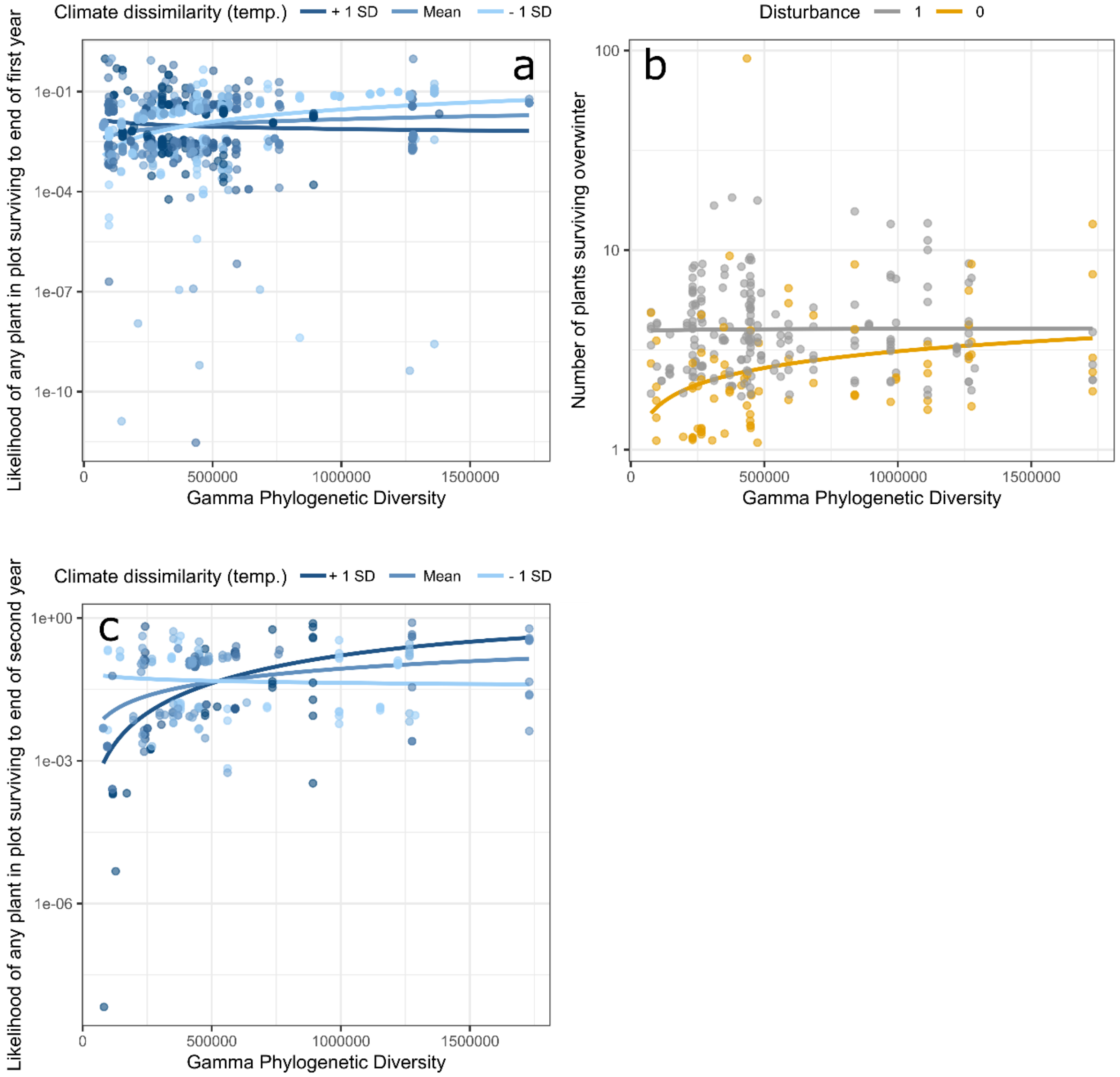


**Figure S11**: Marginal effects plot showing interaction between gamma PD, disturbance and temperature dissimilarity on metrics of seeded species success. Plotted points represent observed data with all other fixed and random effects accounted for.

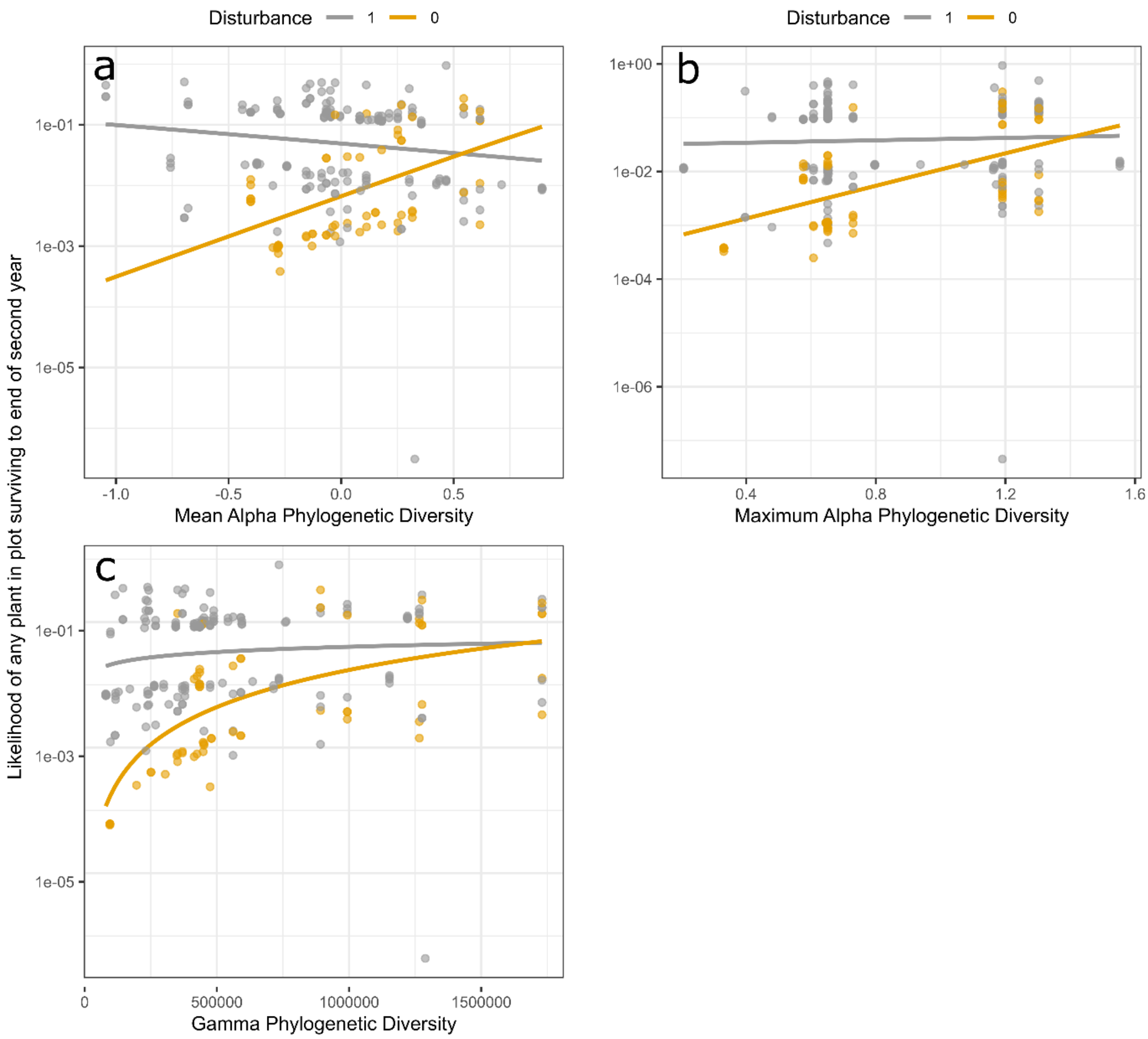


**Figure S12**: Marginal effects plot showing interaction between disturbance and (a) mean, (b) maximum and (c) gamma PD on the likelihood of species surviving to the end of the second growing season. These results are from the 'SC' models. Plotted points represent observed data with all other fixed and random effects accounted for.

*Effect of relative indigenous-PD*

Interactions between relative indigenous-PD and disturbance were also seen when using maximum alpha PD difference (Fig. S13a, c), median PD difference (Fig. S14d) or gamma PD difference (Fig. S15a), and all these interactions were consistent when using the 'SC' and 'Hnd' models (Tables S6-S9). Note that the figures make this trend difficult to see due to needing to correctly scale the y-axis, but in all cases the rate of increase of survival with higher relative indigenous-PD is higher in undisturbed rather than disturbed plots, supporting the trend. This interaction was also seen with median PD difference in the first year, but in the opposite direction (i.e. higher median PD difference only increases survival likelihood in undisturbed plots), potentially because surviving plants had skewed median alpha PD distributions (Fig. S14a, b).

The positive interaction between mean alpha PD difference and temperature dissimilarity was also seen in median PD for both the Haeuser-only and 'K+H' datasets (Figs. S14c, S16c); in other words, high relative indigenous-PD increased survival in the second year when the temperature dissimilarity was higher. The additional models also found interactions with precipitation dissimilarity: higher relative indigenous-PD only helped the *likelihood* of survival when precipitation dissimilarity was *low* (Figs. S15b; S16a, b) but only helped the *number* of plants surviving when precipitation dissimilarity was *high* (Figs. S13b, d; S14e). These effects were largely maintained in the 'SC' models (Tables S7 – S9). In addition, the trend of plants producing a higher number of flowers in more dissimilar precipitation regimes was consistent in the SC models (Tables S6, S7, S9). Cumulatively, these results suggest the same interpretation as in Question 1. Plants need to be similar enough ecologically for high relative indigenous-PD to be beneficial, but for those that can survive, they are more likely to take advantage of high relative indigenous-PD if they are ecologically different.

The above conclusions rely on a slight difference in interpretation of the effects of precipitation dissimilarity and temperature dissimilarity. Specifically, the PD*precipitation dissimilarity interaction is always *negative* for *likelihood* of survival (regardless of growing season) and *positive* for *number of plants* surviving (regardless of growing season). As described above, we have interpreted this as evidence that plants must be similar enough to the local community to have a chance of surviving (and therefore benefit from high indigenous-PD) but are more likely to become abundant if they are slightly different. In

contrast, the PD*temperature dissimilarity interaction is *negative* for likelihood of survival in the *first year*, but *positive* for likelihood of survival in the *second year*. We have also interpreted this as evidence that plants must be similar enough to the local community to have a chance of surviving (and therefore benefit from high indigenous-PD) but are more likely to become abundant if they are slightly different. We suggest that this is because precipitation dissimilarity overall provides a stronger selective pressure than temperature dissimilarity, especially into the second growing season (Figs. 2 and 4, main text) and so imposes a more sustained effect on likelihood of any plants in a plot surviving, as opposed to temperature dissimilarity whose significant negative effects are limited to the first growing season (Fig. 2 main text).

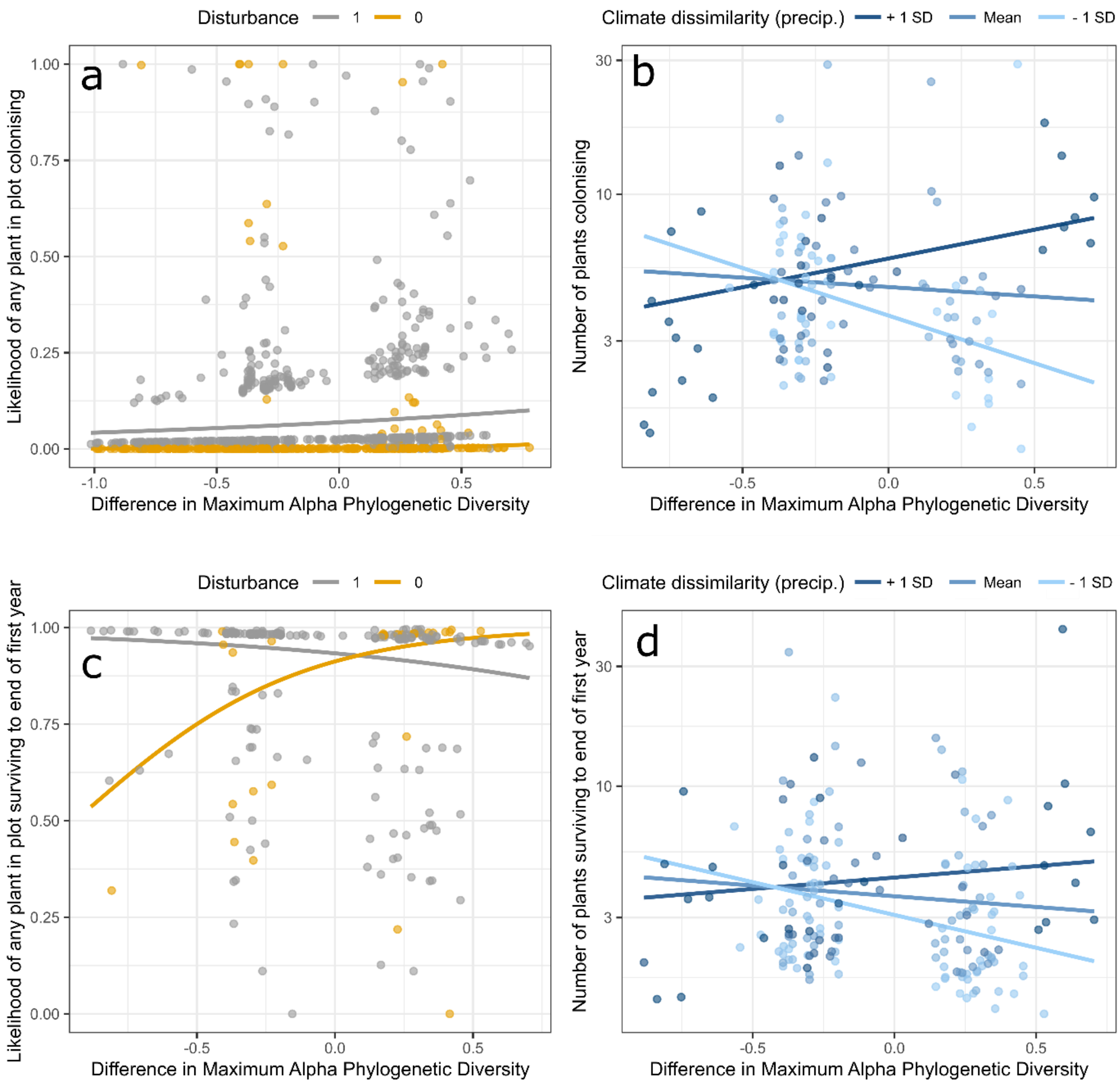


**Figure S13**: Marginal effects plot showing interaction between maximum alpha PD difference and climate dissimilarity or disturbance on plant responses at various time points (Haeuser data). Plotted points represent observed data with all other fixed and random effects accounted for. Note in (a) that the PD*disturbance interaction direction is not obvious, however the rate of increase with higher PD is faster in undisturbed than disturbed plots, so the trend is consistent with that of Figs. 3 and 5 (main text).

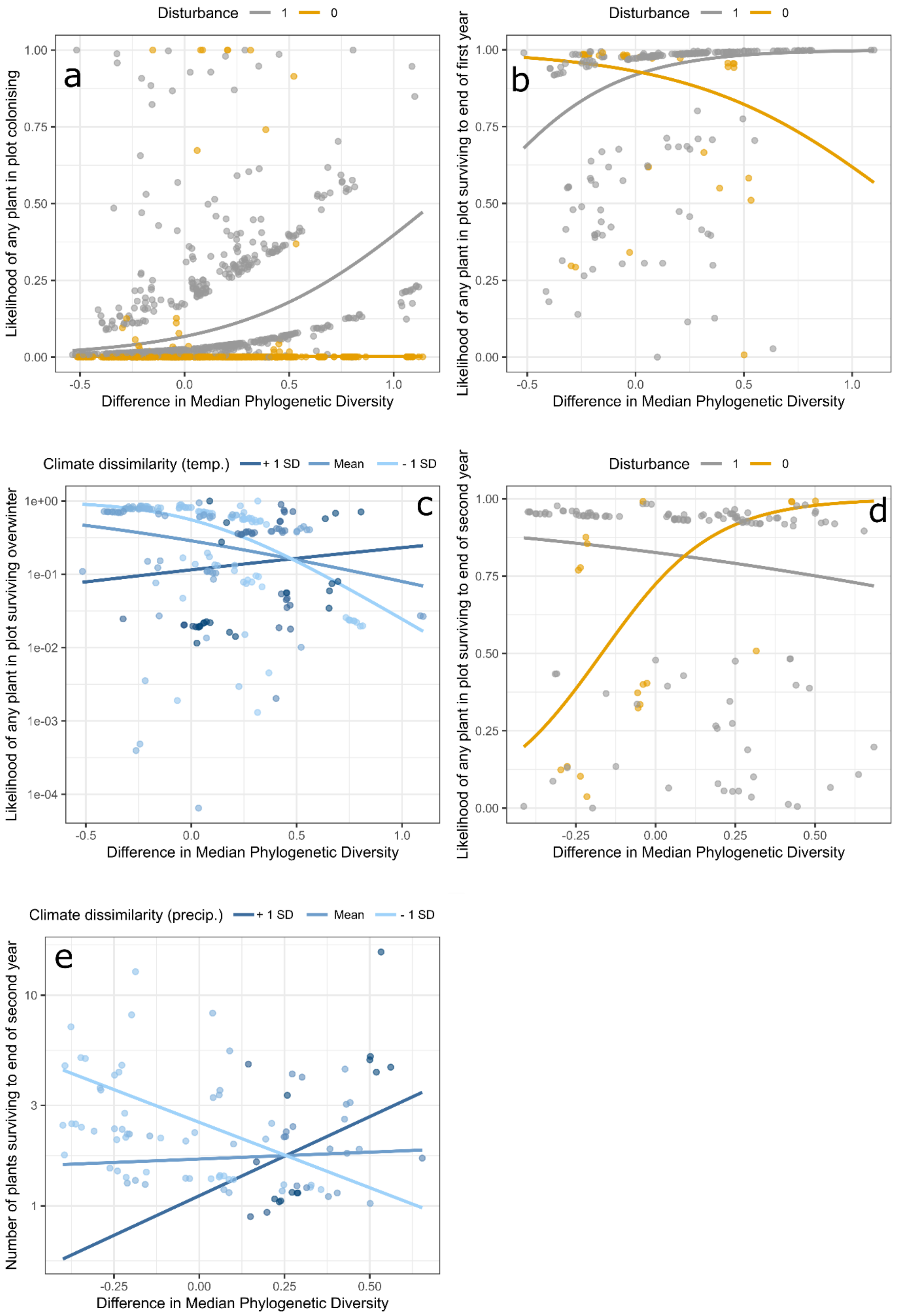


**Figure S14**: Marginal effects plot showing interaction between median alpha PD and climate dissimilarity or disturbance on plant responses at various time points (Haeuser data). Plotted points represent observed data with all other fixed and random effects accounted for.

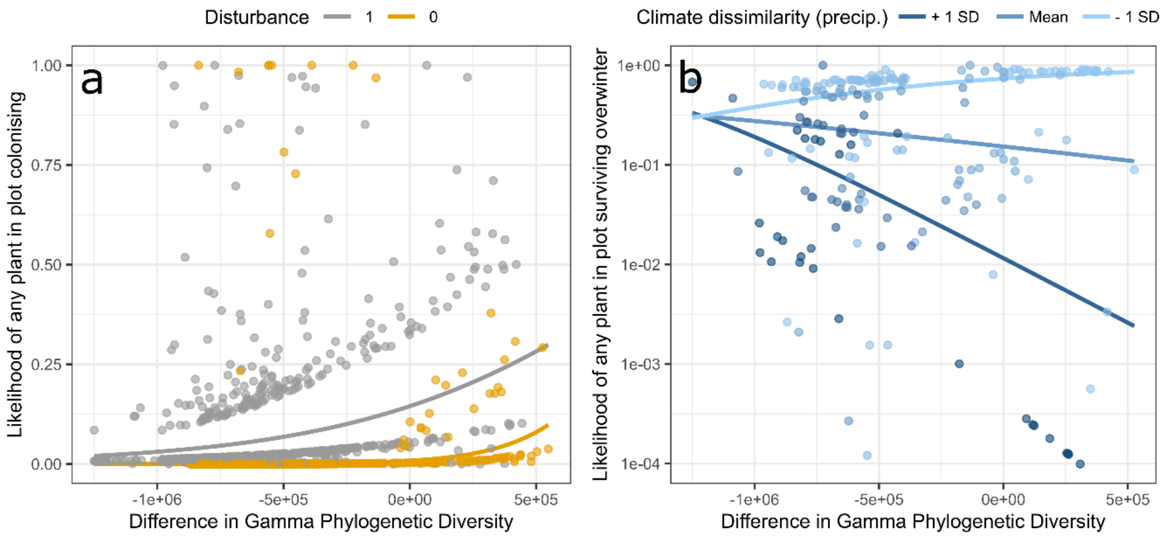


**Figure S15**: Marginal effects plot showing interaction between gamma alpha PD and climate dissimilarity or disturbance on plant responses at various time points (Haeuser data). Plotted points represent observed data with all other fixed and random effects accounted for. Note in (a) that the PD*disturbance interaction direction is not obvious, however the rate of increase with higher PD is faster in undisturbed than disturbed plots, so the trend is consistent with that of Figs. 2 and 4 (main text).

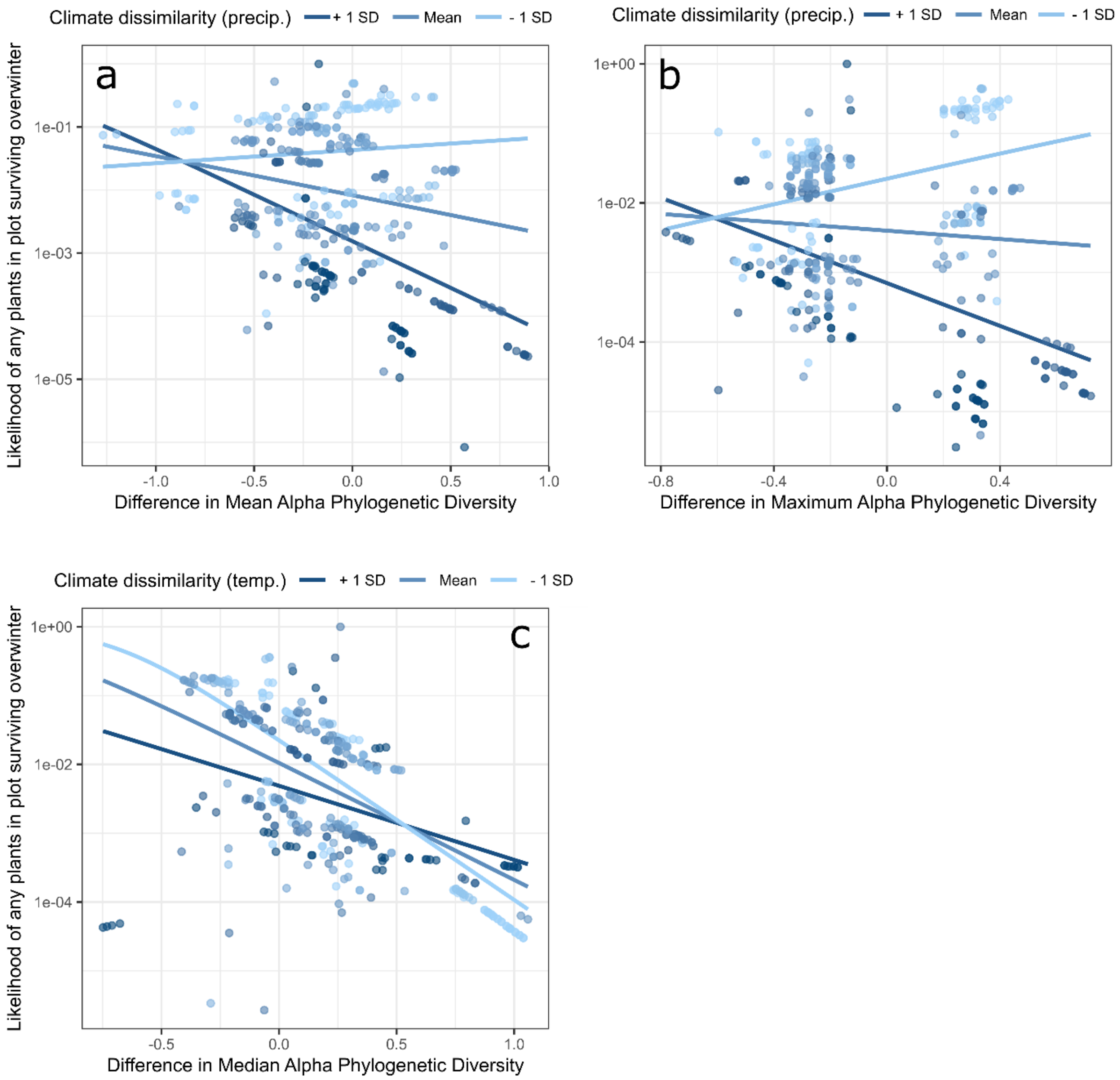


**Figure S16**: Marginal effects plot showing interaction between climate dissimilarity and (a) mean, (b) maximum and (c) median PD on the likelihood of overwinter plant survival, when using the simpler K+H difference in PD metric (see *Supplementary analyses: Robustness tests*). Plotted points represent observed data with all other fixed and random effects accounted for.

*Effect of precipitation dissimilarity*

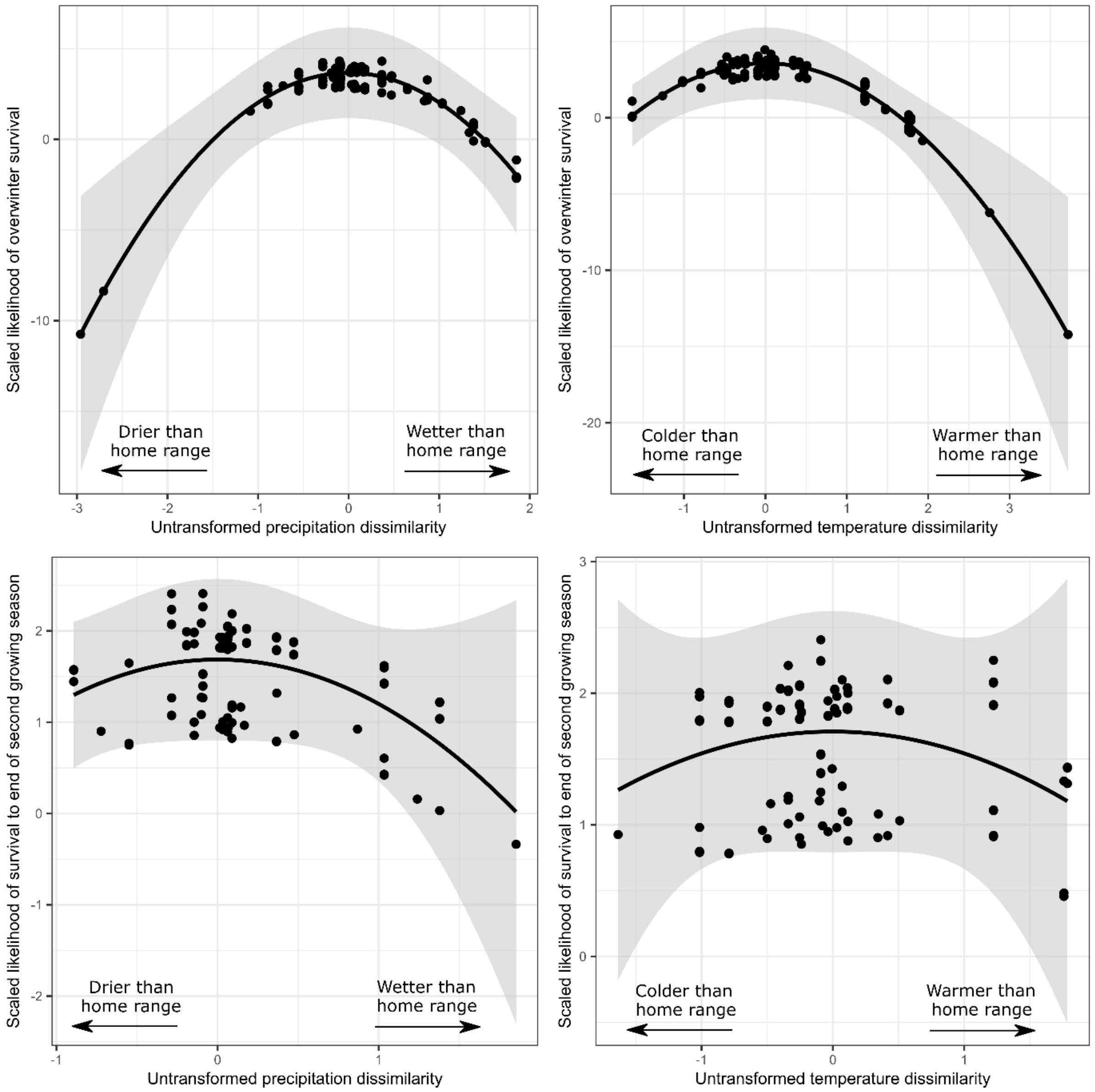


**Figure S17**: The negative relationship between precipitation dissimilarity or temperature dissimilarity and likelihood of overwinter and second-year survival is quadratic: species that came from increasingly wetter or drier climates than the experimental climate experienced lower success, and species that came from increasingly colder or warmer climates experienced lower success. Note that the absolute value of dissimilarity is used in the main model; this plot verifies that higher untransformed dissimilarity in both directions leads to lower survival.

*Species origin and trait inclusion*

In all cases, results held for both native and non-native species, and for species from different biogeographic regions (Table S10). Adding either of these variables did not add species origin added no information for 28 of 32 models (AIC within 2 of main models; species origin p-value > 0.05; Table S10); in the four cases where species origin was significant, the effect and significance of indigenous-PD was unchanged, suggesting that high indigenous-PD provides a benefit for establishment of both native and non-native species, and for non-native species regardless of specific continental origin and is therefore a general force contributing to community assembly and structure. There was one additional case where biogeographic origin was not significant, but its inclusion removed a significant PD*disturbance interaction (Table S10); in this case, the main effect of indigenous-PD remained positive and significant.

Models where the effect of indigenous-PD was mediated entirely by species traits were consistently inferior to those where indigenous-PD was allowed to have a direct effect (Table S11). Further, when including traits, indigenous-PD was still a significant positive predictor (where sample size allowed). While the included traits were also related to species success in the models, particularly seed mass, these results suggest that the positive effect of indigenous-PD detected in our study cannot be explained purely by SLA, plant height and seed mass. This indicates that indigenous-PD provides a holistic measure of fitness with value in its own right. We do note that in the Question 2 models, the significant effect of indigenous-PD was sometimes lost (Table S11). However, this was due to the significantly lower sample size, necessitated by only including species for which there was appropriate trait data. We found that the significance of indigenous-PD was lost even when not including trait data (i.e. an identical model to the main model but only using the species which had trait data), confirming that the lost significance was due to sample size and not trait inclusion. However, even in these cases, the models including a direct indigenous-PD effect were still substantially preferred to models not including this effect (over 2 AIC lower), strongly suggesting that having a direct effect of indigenous-PD is still substantially preferred to modelling indigenous-PD only though traits, even if indigenous-PD does not reach the $p<0.05$ threshold.

*Supplementary tables for effect of absolute indigenous-PD*

**Table S2**: Summary of results, using *mean alpha phylogenetic diversity* as response variable. Bold indicates significant p-value. In the p-value column, the superscript K+M indicates the significance (either significant or not significant) was the same in the reduced model just using the Kempel and Muller datasets; the superscript K+H indicates the significance was the same in the reduced model using the Kempel and Haeuser datasets using the (modified) difference in phylogenetic diversity; the superscript SC indicates the significance was the same in the models using single climate variables instead of principal components. Note that the K+H models did not include herbivory (indicated by NA), as only the Müller dataset had a closed herbivory treatment. In the effect size column, the effect size in brackets is the scaled effect size (centred divided by 2 standard deviations). Categorical variables were not scaled.

| Response | Stage | Explanatory variable | Effect size | p-value |
|---|---|---|---|---|
| **Any plants in plot** | Colonisation | Mean alpha PD | -0.281 (-0.231) | 0.522 $^{K+M; K+H; SC}$ |
| | | Disturbed | 1.239 | **<0.001** $^{K+M; K+H; SC}$ |
| | | Climate diss. (precip.) | 0.038 (0.060) | 0.897 $^{K+M; K+H; SC}$ |
| | | Climate diss. (temp.) | 0.037 (0.038) | 0.916 $^{K+M; K+H; SC}$ |
| | First growing season | Mean alpha PD | 2.244 (1.881) | **0.006** $^{K+M; K+H; SC}$ |
| | | Disturbed | 1.047 | **0.001** $^{K+M; K+H; SC}$ |
| | | Climate diss. (precip.) | -2.502 (-3.774) | **<0.001** $^{K+M; K+H}$ |
| | | Climate diss. (temp.) | 0.712 (0.679) | 0.275 $^{K+M; K+H; SC}$ |
| | | Herbivory | -0.628 | 0.096 $^{K+M; NA; SC}$ |
| | Overwinter | Mean alpha PD | -0.332 (-0.299) | 0.586 $^{K+M; K+H; SC}$ |
| | | Disturbed | 1.895 | **<0.001** $^{K+M; SC}$ |
| | | Climate diss. (precip.) | -1.184 (-1.665) | **0.005** $^{K+M; K+H; SC}$ |
| | | Climate diss. (temp.) | -0.978 (-0.888) | 0.098 $^{K+M; K+H; SC}$ |
| | | Herbivory | -0.960 | **0.001** $^{K+M; NA; SC}$ |
| | Second growing season | Mean alpha PD | -1.431 (0.391) | 0.186 $^{K+M; K+H; SC}$ (0.489) |
| | | Disturbed | 1.372 | **0.028** $^{K+H; SC}$ |
| | | Climate diss. (precip.) | -2.313 (-2.724) | **0.001** $^{K+M; K+H; SC}$ |
| | | Climate diss. (temp.) | -1.026 (-0.995) | 0.138 $^{K+M; K+H; SC}$ (0.152) |
| | | Mean alpha PD*Climate diss. (temp.) | 3.249 (2.385) | **0.013** $^{K+H}$ |
| **Number of plants in plot** | Colonisation | Mean alpha PD | 0.339 (0.283) | 0.120 $^{K+M; K+H; SC}$ |
| | | Disturbed | 0.228 (0.248) | **0.004** $^{K+M; K+H; SC}$ (**0.001**) |

| | | | | |
|---|---|---|---|---|
| | | Climate diss. (precip.) | 0.226 (0.337) | 0.072 K+M; SC |
| | | Climate diss. (temp.) | -0.355 (-0.317) | **0.032** K+H |
| | | Mean alpha PD* Disturbed | -0.271 (-0.226) | **0.033** K+M; K+H; SC |
| | First growing season | Mean alpha PD | 0.588 (0.536) | **0.004** K+M; K+H; SC |
| | | Disturbed | 0.153 (0.159) | **0.026** K+M; SC (**0.020**) |
| | | Climate diss. (precip.) | -0.001 (<0.001) | 0.999 K+M; K+H; SC |
| | | Climate diss. (temp.) | -0.361 (-0.332) | **0.018** |
| | | Herbivory | -0.091 | 0.198 K+M; NA; SC |
| | | Mean alpha PD* Disturbed | -0.237 (-0.216) | **0.047** K+M; K+H; SC |
| | Overwinter | Mean alpha PD | 0.244 (0.197) | 0.280 K+M; K+H; SC |
| | | Disturbed | 0.464 | **0.001** K+M; SC |
| | | Climate diss. (precip.) | 0.234 (0.293) | 0.180 K+M; K+H; SC |
| | | Climate diss. (temp.) | -0.214 (-0.135) | 0.490 K+M; K+H; SC |
| | | Herbivory | -0.030 | 0.800 K+M; NA; SC |
| | Second growing season | Mean alpha PD | 0.174 (0.123) | 0.530 K+H; SC |
| | | Disturbed | 0.076 | 0.688 K+M; K+H; SC |
| | | Climate diss. (precip.) | 0.442 (0.362) | 0.162 K+M; K+H; SC |
| | | Climate diss. (temp.) | 0.017 (0.012) | 0.956 K+M, K+H; SC |

**Table S3**: Summary of results, using *maximum alpha phylogenetic diversity* as response variable. Bold indicates significant p-value. In the p-value column, the superscript K+M indicates the significance (either significant or not significant) was the same in the reduced model just using the Kempel and Muller datasets; the superscript K+H indicates the significance was the same in the reduced model using the Kempel and Haeuser datasets using the (modified) difference in phylogenetic diversity; the superscript SC indicates the significance was the same in the models using single climate variables instead of principal components.. Note that the K+H models did not include herbivory (indicated by NA), as only the Müller dataset had a closed herbivory treatment. In the effect size column, the effect size in brackets is the scaled effect size (centred divided by 2 standard deviations). Categorical variables were not scaled.

| Response | Stage | Explanatory variable | Effect size | p-value |
|---|---|---|---|---|
| **Any plants in plot** | Colonisation | Maximum alpha PD | -0.134 (-0.084) | 0.814 [K+M; K+H; SC] |
| | | Disturbed | 1.239 | **<0.001** [K+M; K+H; SC] |
| | | Climate diss. (precip.) | 0.022 (0.034) | 0.940 [K+M; K+H; SC] |
| | | Climate diss. (temp.) | 0.065 (0.067) | 0.853 [K+M; K+H; SC] |
| | First growing season | Maximum alpha PD | 3.487 (2.268) | **0.001** [SC] |
| | | Disturbed | 2.559 (0.839) | **<0.001** [K+M; K+H; SC] (**0.013**) |
| | | Climate diss. (precip.) | -2.094 (-3.159) | **0.001** [K+M; K+H] |
| | | Climate diss. (temp.) | 0.499 (0.476) | 0.388 [K+M; K+H; SC] |
| | | Herbivory | -0.646 | 0.092 [K+M; NA; SC] |
| | | Maximum alpha PD * Disturbed | -2.012 (-1.309) | **0.005** [K+M; SC] |
| | Overwinter | Maximum alpha PD | 2.882 (0.514) | **0.022** [K+H; SC] (0.347) |
| | | Disturbed | 1.912 | **<0.001** [K+M; SC] |
| | | Climate diss. (precip.) | 1.308 (-1.756) | 0.193 [K+M] (**0.004**) |
| | | Climate diss. (temp.) | -1.008 (-0.918) | 0.092 [K+M; K+H] |
| | | Herbivory | -0.973 | **0.006** [K+M; NA; SC] |
| | | Maximum alpha PD*Climate diss. (precip.) | -2.859 (-2.665) | **0.008** [K+M; K+H] |
| | Second growing season | Maximum alpha PD | 0.777 (0.496) | 0.405 [K+M; K+H; SC] |
| | | Disturbed | 1.327 | **0.029** [K+H; SC] |
| | | Climate diss. (precip.) | -2.183 (-2.571) | **0.001** [K+H; SC] |
| | | Climate diss. (temp.) | -0.664 (-0.673) | 0.291 [K+M; K+H; SC] |
| **Number of plants in plot** | Colonisation | Maximum alpha PD | 0.397 (0.247) | 0.141 [K+M; SC] |
| | | Disturbed | 0.708 (0.262) | **<0.001** [K+M; K+H; SC] |
| | | Climate diss. (precip.) | 0.226 (0.336) | 0.072[; SC] |

| | | | | |
|---|---|---|---|---|
| | | Climate diss. (temp.) | -0.385 (-0.343) | **0.016** K+H |
| | | Maximum alpha PD * Disturbed | -0.549 (-0.342) | **<0.001** K+M; K+H; SC |
| | First growing season | Maximum alpha PD | 0.884 (0.587) | **<0.001** K+M; K+H; SC |
| | | Disturbed | 0.766 (0.205) | **<0.001** K+M; SC (**0.003**) |
| | | Climate diss. (precip.) | -0.013 (-0.018) | 0.918 K+M; K+H; SC |
| | | Climate diss. (temp.) | -0.401 (-0.369) | **0.007** K+H |
| | | Herbivory | -0.090 | 0.201 K+M; NA; SC |
| | | Maximum alpha PD* Disturbed | -0.620 (-0.412) | **<0.001** K+M; K+H; SC |
| | Overwinter | Maximum alpha PD | -0.051 (-0.031) | 0.867 K+M; K+H; SC |
| | | Disturbed | 0.451 | **0.001** K+M; SC |
| | | Climate diss. (precip.) | 0.218 (0.273) | 0.213 K+M; K+H; SC |
| | | Climate diss. (temp.) | -0.213 (-0.134) | 0.497 K+M; K+H; SC |
| | | Herbivory | -0.025 | 0.832 K+M; NA; SC |
| | Second growing season | Maximum alpha PD | -0.181 (-0.107) | 0.594 K+M; K+H; SC |
| | | Disturbed | 0.038 | 0.845 K+M; K+H; SC |
| | | Climate diss. (precip.) | 0.416 (0.340) | 0.195 K+M; K+H; SC |
| | | Climate diss. (temp.) | 0.050 (0.035) | 0.871 K+M; K+H; SC |

**Table S4**: Summary of results, using *median alpha phylogenetic diversity* as response variable. Bold indicates significant p-value. In the p-value column, the superscript K+M indicates the significance (either significant or not significant) was the same in the reduced model just using the Kempel and Muller datasets; the superscript K+H indicates the significance was the same in the reduced model using the Kempel and Haeuser datasets using the (modified) difference in phylogenetic diversity; the superscript SC indicates the significance was the same in the models using single climate variables instead of principal components.. Note that the K+H models did not include herbivory (indicated by NA), as only the Müller dataset had a closed herbivory treatment. In the effect size column, the effect size in brackets is the scaled effect size (centred divided by 2 standard deviations). Categorical variables were not scaled.

| **Response** | **Stage** | **Explanatory variable** | **Effect size** | **p-value** |
|---|---|---|---|---|
| **Any plants in plot** | Colonisation | Median alpha PD | 1.105 (0.715) | 0.072 [K+M; K+H; SC] |
| | | Disturbed | 1.317 (-1.151) | **<0.001** [K+M; K+H; SC] |
| | | Climate diss. (precip.) | 0.091 (-0.142) | 0.767 [K+M; K+H; SC] |
| | | Climate diss. (temp.) | 0.201 (0.208) | 0.579 [K+M; K+H; SC] |
| | | Median alpha PD*Disturbed | -0.839 (-0.543) | **0.016** [K+M; SC] |
| | First growing season | Median alpha PD | 2.417 (1.623) | **0.023** [K+M; K+H; SC] |
| | | Disturbed | 1.018 | **0.001** [K+M; K+H; SC] |
| | | Climate diss. (precip.) | -2.832 (-4.272) | **<0.001** [K+M; K+H; SC] |
| | | Climate diss. (temp.) | 0.947 (0.904) | 0.168 [K+M; SC] |
| | | Herbivory | -0.630 | 0.094 [K+M; NA; SC] |
| | Overwinter | Median alpha PD | -5.103 (-2.666) | **<0.001** [K+H; SC] |
| | | Disturbed | 1.997 | **<0.001** [K+M; K+H; SC] |
| | | Climate diss. (precip.) | -0.401 (-0.564) | 0.319 [K+M] |
| | | Climate diss. (temp.) | -2.179 (1.519) | **0.002** [K+M; K+H] |
| | | Herbivory | -0.944 | **0.001** [K+M; NA; SC] |
| | | Median alpha PD * Climate diss. (temp.) | 2.480 (1.513) | **0.028** [K+H] |
| | Second growing season | Median alpha PD | -2.642 (-0.343) | 0.059 [K+M; K+H; SC] (0.588) |
| | | Disturbed | 1.426 | **0.021** [K+H; SC] |
| | | Climate diss. (precip.) | -1.977 (-2.329) | **0.002** [K+H; SC] |
| | | Climate diss. (temp.) | -1.911 (-1.171) | **0.049** (0.108) |
| | | Median alpha PD * Climate diss. (temp.) | 3.342 (1.889) | **0.043** [K+H] |
| **Number of plants in plot** | Colonisation | Median alpha PD | 1.590 (0.848) | **<0.001** [K+M; K+H; SC] |
| | | Disturbed | 0.346 (0.238) | **<0.001** [K+M; K+H; SC] (**0.002**) |

| | | | | |
|---|---|---|---|---|
| | | Climate diss. (precip.) | 0.058 (0.086) | 0.647 [K+M; SC] |
| | | Climate diss. (temp.) | -0.045 (-0.147) | 0.792 [K+M; K+H; SC] (0.309) |
| | | Median alpha PD*Disturbed | -0.482 (-0.306) | **0.002** [K+M; K+H; SC] |
| | | Median alpha PD * Climate diss. (temp.) | -0.537 (-0.303) | **0.033** [K+H] |
| | First growing season | Median alpha PD | 0.960 (0.650) | **<0.001** [K+M, K+H; SC] |
| | | Disturbed | 0.213 (0.123) | **0.002** [K+M; SC] (0.080) |
| | | Climate diss. (precip.) | -0.097 (-0.135) | 0.495 [K+M; K+H; SC] |
| | | Climate diss. (temp.) | -0.253 (-0.233) | 0.100 [K+M; K+H; SC] |
| | | Herbivory | -0.090 | 0.204 [K+M; NA; SC] |
| | | Median alpha PD* Disturbed | -0.425 (-0.287) | **0.012** [K+M; K+H; SC] |
| | Overwinter | Median alpha PD | 0.299 (0.138) | 0.410 [K+M; SC] |
| | | Disturbed | 0.449 | **0.001** [K+M; SC] |
| | | Climate diss. (precip.) | 0.200 (0.250) | 0.253 [K+M; K+H; SC] |
| | | Climate diss. (temp.) | -0.216 (-0.135) | 0.483 [K+M; SC] |
| | | Herbivory | -0.029 | 0.802 [K+M; NA; SC] |
| | Second growing season | Median alpha PD | -1.166 (-0.044) | 0.064 [K+H, K+H; SC] (0.820) |
| | | Disturbed | -0.016 | 0.935 [K+M; K+H; SC] |
| | | Climate diss. (precip.) | -0.158 (0.135) | 0.724 [K+M; K+H; SC] (0.635) |
| | | Climate diss. (temp.) | 0.239 (0.168) | 0.439 [K+M; K+H; SC] |
| | | Median alpha PD * Climate diss. (temp.) | 1.964 (0.795) | **0.042** [K+H; SC] |

**Table S5**: Summary of results, using *gamma phylogenetic diversity* as response variable. Bold indicates significant p-value. In the p-value column, the superscript K+M indicates the significance (either significant or not significant) was the same in the reduced model just using the Kempel and Muller datasets; the superscript K+H indicates the significance was the same in the reduced model using the Kempel and Haeuser datasets using the (modified) difference in phylogenetic diversity; the superscript SC indicates the significance was the same in the models using single climate variables instead of principal components.. Note that the K+H models did not include herbivory (indicated by NA), as only the Müller dataset had a closed herbivory treatment. In the effect size column, the effect size in brackets is the scaled effect size (centred divided by 2 standard deviations). Categorical variables were not scaled.

| Response | Stage | Explanatory variable | Effect size | p-value |
|---|---|---|---|---|
| **Any plants in plot** | Colonisation | Gamma PD | -0.329 (-0.464) | 0.192 [K+M, K+H; SC] |
| | | Disturbed | 1.238 | **<0.001** [K+M; K+H; SC] |
| | | Climate diss. (precip.) | -0.072 (-0.113) | 0.808 [K+M; K+H; SC] |
| | | Climate diss. (temp.) | 0.055 (0.057) | 0.873 [K+H; SC] |
| | First growing season | Gamma PD | 1.325 (0.733) | **0.034** [K+H; SC] (0.266) |
| | | Disturbed | 1.017 | **0.001** [K+M; K+H; SC] |
| | | Climate diss. (precip.) | -2.305 (-3.477) | **<0.001** [K+M; K+H] |
| | | Climate diss. (temp.) | 20.085 (0.105) | **0.038** [K+H] (0.868) |
| | | Herbivory | -0.616 | 0.102 [K+M; NA; SC] |
| | | Gamma PD * Climate diss. (temp.) | -1.558 (-2.169) | **0.042** [K+H] |
| | Overwinter | Gamma PD | 0.371 (0.560) | 0.275 [K+M; K+H SC] |
| | | Disturbed | 1.906 | **<0.001** [K+M; K+H; SC] |
| | | Climate diss. (precip.) | -1.096 (-1.540) | **0.010** [K+H; SC] |
| | | Climate diss. (temp.) | -0.908 (-0.827) | 0.115 [K+M; K+H; SC] |
| | | Herbivory | -0.970 | **0.001** [K+M; NA; SC] |
| | Second growing season | Gamma PD | -0.369 (1.488) | 0.564 [K+M; K+H; SC] (**0.032**) |
| | | Disturbed | 1.488 | **0.021** [K+H; SC] |
| | | Climate diss. (precip.) | -2.235 (-2.633) | **0.001** [K+H; SC] |
| | | Climate diss. (temp.) | -29.613 (-0.401) | **0.034** (0.571) |
| | | Gamma PD * Climate diss. (temp.) | 2.248 (3.411) | **0.035** |
| **Number of plants in plot** | Colonisation | Gamma PD | -0.091 (-0.139) | 0.391 [K+H; SC] |
| | | Disturbed | 0.260 | **0.001** [K+M; SC] |
| | | Climate diss. (precip.) | 0.213 (0.318) | 0.087 [SC] |

| | | | | |
|---|---|---|---|---|
| | | Climate diss. (temp.) | -0.397 (-0.354) | **0.011** [K+H] |
| | First growing season | Gamma PD | 0.144 (0.218) | 0.155 [K+M; K+H; SC] |
| | | Disturbed | 0.175 | **0.011** [K+M; SC] |
| | | Climate diss. (precip.) | 0.085 (0.118) | 0.538 [K+M; K+H; SC] |
| | | Climate diss. (temp.) | -0.425 (-0.392) | **0.005** [K+M; K+H] |
| | | Herbivory | -0.090 | 0.207 [K+M; NA; SC] |
| | Overwinter | Gamma PD | 0.277 (0.406) | 0.098 [K+M; K+H; SC] |
| | | Disturbed | 3.974 (0.475) | **0.027** [K+M; SC] (**<0.001**) |
| | | Climate diss. (precip.) | 0.311 (0.389) | 0.104 [K+M; K+H; SC] |
| | | Climate diss. (temp.) | -0.245 (-0.154) | 0.442 [K+M; K+H; SC] |
| | | Herbivory | -0.028 | 0.811 [K+M; NA; SC] |
| | | Gamma PD * Disturbed | -0.269 (-0.394) | **0.050** [SC] |
| | Second growing season | Gamma PD | 0.095 (0.120) | 0.546 [K+M; K+H; SC] |
| | | Disturbed | -0.087 | 0.652 [K+M; K+H; SC] |
| | | Climate diss. (precip.) | 0.468 (0.383) | 0.146 [K+M; K+H; SC] |
| | | Climate diss. (temp.) | 0.016 (0.011) | 0.958 [K+M; K+H; SC] |

*Supplementary tables for effect of relative indigenous-PD*

**Table S6**: Summary of results, using *difference in mean alpha phylogenetic diversity* (planted species PD minus abundance-weighted community PD) as response variable. Bold indicates significant p-value. In the p-value column, the superscript Hnd indicates the significance (either significant or not significant) was the same in a model using raw phylogenetic diversity (rather than the difference); the superscript SC indicates the significance was the same in the models using single climate variables instead of principal components. In the effect size column, the effect size in brackets is the scaled effect size (centred divided by 2 standard deviations). Categorical variables were not scaled.

| **Response** | **Stage** | **Explanatory variable** | **Effect size** | **p-value** |
|---|---|---|---|---|
| **Any plants in plot** | Colonisation | Mean alpha PD diff. | 1.881 (1.827) | **0.009** [Hnd; SC] |
| | | Disturbed | 3.644 | **<0.001** [Hnd; SC] |
| | | Climate diss. (precip.) | -0.909 (-1.273) | 0.126 [Hnd; SC] |
| | | Climate diss. (temp.) | -0.183 (0.273) | 0.743 [Hnd; SC] |
| | | Heating | -0.855 | **0.004** [Hnd; SC] |
| | | awMPD | -0.325 (-0.650) | **0.045** [SC] |
| | First growing season | Mean alpha PD diff. | 1.743 (1.471) | **0.005** [Hnd; SC] |
| | | Disturbed | 0.409 | 0.467 [Hnd; SC] |
| | | Climate diss. (precip.) | -0.306 (-0.357) | 0.522 [Hnd; SC] |
| | | Climate diss. (temp.) | 0.542 (0.641) | 0.279 [Hnd; SC] |
| | | Heating | -1.029 | **0.006** [Hnd; SC] |
| | | awMPD | -0.045 (-0.091) | 0.847 [Hnd; SC] |
| | Overwinter | Mean alpha PD diff. | -2.460 (-0.825) | **0.050** (0.256) |
| | | Disturbed | 1.237 | **0.047** [Hnd] |
| | | Climate diss. (precip.) | -2.148 (2.403) | **<0.001** [Hnd; SC] |
| | | Climate diss. (temp.) | -0.692 (-1.275) | 0.252 [Hnd;SC] (0.095) |
| | | Heating | 0.798 | 0.065 [Hnd; SC] |
| | | awMPD | -0.388 (-0.769) | 0.167 [Hnd; SC] |
| | | Mean alpha PD diff. * Climate diss. (temp.) | 2.896 (2.831) | **0.053** [Hnd] |
| | Second growing season | Mean alpha PD diff. | 13.779 (10.292) | 0.054 [Hnd; SC] |
| | | Disturbed | 2.265 (4.624) | 0.080 [Hnd] (0.052) |
| | | Climate diss. (precip.) | -0.904 (-0.724) | 0.198 [Hnd; SC] |
| | | Climate diss. (temp.) | 0.012 (0.012) | 0.983 [Hnd; SC] |
| | | Heating | -0.936 | **0.033** [SC] |
| | | awMPD | -0.100 (-0.199) | 0.675 [Hnd; SC] |
| | | Mean alpha PD diff. * Disturbed | -13.859 (-10.352) | **0.052** [SC] |

| | | | | |
|---|---|---|---|---|
| | Flowering* | Mean alpha PD diff. | 0.986 (0.963) | 0.305 [Hnd; SC] |
| | | Climate diss. (precip.) | -0.689 (-0.965) | 0.412 [Hnd; SC] |
| | | Climate diss. (temp.) | -0.454 (-0.716) | 0.614 [Hnd; SC] |
| | | Heating | -0.649 | 0.072 [Hnd; SC] |
| | | awMPD | 0.011 (0.022) | 0.967 [Hnd; SC] |
| **Number of plants in plot** | Colonisation | Mean alpha PD diff. | 0.839 (0.642) | **0.006** [Hnd; SC] |
| | | Disturbed | 1.568 | **<0.001** [Hnd; SC] |
| | | Climate diss. (precip.) | 0.113 (0.141) | 0.579 [Hnd; SC] |
| | | Climate diss. (temp.) | -0.287 (-0.327) | 0.223 [Hnd; SC] |
| | | Heating | -0.310 | **0.013** [Hnd; SC] |
| | | awMPD | 0.057 (0.114) | 0.321 [Hnd; SC] |
| | First growing season | Mean alpha PD diff. | 0.597 (0.455) | **0.003** [Hnd; SC] |
| | | Disturbed | -0.018 | 0.915 [Hnd; SC] |
| | | Climate diss. (precip.) | 0.122 (0.141) | 0.397 [Hnd; SC] |
| | | Climate diss. (temp.) | -0.219 (-0.274) | 0.103 [Hnd; SC] |
| | | Heating | -0.237 | **0.037** [Hnd; SC] |
| | | awMPD | -0.086 (-0.171) | 0.209 [Hnd; SC] |
| | Overwinter | Mean alpha PD diff. | 0.336 (0.243) | 0.165 [Hnd; SC] |
| | | Disturbed | 0.151 | 0.533 [Hnd; SC] |
| | | Climate diss. (precip.) | 0.404 (0.334) | **0.044** [Hnd] |
| | | Climate diss. (temp.) | -0.145 (-0.127) | 0.471 [Hnd; SC] |
| | | Heating | -0.139 | 0.262 [Hnd; SC] |
| | | awMPD | 0.111 (0.221) | 0.157 [Hnd; SC] |
| | Second growing season | Mean alpha PD diff. | 0.367 (0.272) | 0.233 [Hnd; SC] |
| | | Disturbed | 0.084 | 0.773 [Hnd; SC] |
| | | Climate diss. (precip.) | 0.488 (0.381) | 0.084 [Hnd; SC] |
| | | Climate diss. (temp.) | -0.163 (-0.152) | 0.512 [Hnd; SC] |
| | | Heating | -0.051 | 0.735 [Hnd; SC] |
| | | awMPD | 0.018 (0.035) | 0.861 [Hnd; SC] |
| | Flowering* | Mean alpha PD diff. | -0.145 (-0.139) | 0.481 [Hnd; SC] |
| | | Climate diss. (precip.) | 0.357 (0.432) | **0.028** [Hnd; SC] |
| | | Climate diss. (temp.) | -0.306 (-0.355) | 0.100 [Hnd; SC] |
| | | Heating | 0.081 | 0.518 [Hnd; SC] |
| | | awMPD | -0.096 (-0.191) | 0.237 [Hnd; SC] |

*Plants only flowered in disturbed plots, so these models exclude disturbance

**Table S7**: Summary of results, using *difference in maximum alpha phylogenetic diversity* (planted species PD minus abundance-weighted community PD) as response variable. Bold indicates significant p-value. In the p-value column, the superscript Hnd indicates the significance (either significant or not significant) was the same in a model using raw phylogenetic diversity (rather than the difference); the superscript SC indicates the significance was the same in the models using single climate variables instead of principal components. In the effect size column, the effect size in brackets is the scaled effect size (centred divided by 2 standard deviations). Categorical variables were not scaled.

| Response | Stage | Explanatory variable | Effect size | p-value |
|---|---|---|---|---|
| **Any plants in plot** | Colonisation | Maximum alpha PD diff. | 2.298 (1.742) | **0.046** Hnd; SC |
| | | Disturbed | 3.649 (3.848) | **<0.001** Hnd; SC |
| | | Climate diss. (precip.) | -0.817 (-1.144) | 0.199 Hnd; SC |
| | | Climate diss. (temp.) | 0.320 (0.476) | 0.588 Hnd; SC |
| | | Heating | -0.874 | **0.009** Hnd; SC |
| | | awMPD | -0.012 (-0.522) | 0.112 Hnd; SC |
| | | Maximum alpha PD diff. * Disturbed | -1.776 (-1.347) | **0.034** Hnd; SC |
| | First growing season | Maximum alpha PD diff. | 2.488 (1.748) | 0.165 Hnd; SC |
| | | Disturbed | 0.296 (0.445) | 0.623 SC (0.460) |
| | | Climate diss. (precip.) | -0.406 (-0.474) | 0.465 Hnd; SC |
| | | Climate diss. (temp.) | 0.632 (0.748) | 0.261 Hnd; SC |
| | | Heating | -1.115 | **0.004** Hnd; SC |
| | | awMPD | -0.001 (-0.003) | 0.995 Hnd; SC |
| | | Maximum alpha PD diff.* Disturbed | -3.542 (-2.488) | **0.041** Hnd; SC |
| | Overwinter | Maximum alpha PD diff. | -0.786 (-0.549) | 0.419 Hnd; SC |
| | | Disturbed | 1.192 | 0.058 Hnd; SC |
| | | Climate diss. (precip.) | -2.485 (-2.780) | **0.001** Hnd; SC |
| | | Climate diss. (temp.) | -0.825 (0.969) | 0.190 Hnd; SC |
| | | Heating | 0.845 | 0.051 Hnd; SC |
| | | awMPD | -0.379 (-0.758) | 0.181 Hnd; SC |
| | Second growing season | Maximum alpha PD diff. | 0.188 (0.117) | 0.824 Hnd; SC |
| | | Disturbed | 0.727 | 0.259 Hnd; SC |
| | | Climate diss. (precip.) | -0.672 (-0.538) | 0.325 Hnd; SC |
| | | Climate diss. (temp.) | 0.135 (0.129) | 0.789 Hnd; SC |
| | | Heating | -0.756 | 0.068 Hnd; SC |
| | | awMPD | -0.059 (-0.118) | 0.799 Hnd; SC |
| | Flowering* | Maximum alpha PD diff. | 0.168 (0.127) | 0.889 Hnd; SC |

| | | | | |
|---|---|---|---|---|
| | | Climate diss. (precip.) | -0.666 (-0.933) | 0.443 [Hnd; SC] |
| | | Climate diss. (temp.) | -0.468 (-0.696) | 0.569 [Hnd; SC] |
| | | Heating | -0.701 | 0.053 [Hnd; SC] |
| | | awMPD | 0.021 (0.042) | 0.938 [Hnd; SC] |
| **Number of plants in plot** | Colonisation | Maximum alpha PD diff. | -0.865 (-0.613) | 0.102 [Hnd; SC] |
| | | Disturbed | 1.545 | **<0.001** [Hnd; SC] |
| | | Climate diss. (precip.) | 0.375 (0.246) | 0.113 [Hnd; SC] (0.276) |
| | | Climate diss. (temp.) | -0.339 (-0.441) | 0.883 [Hnd; SC] |
| | | Heating | -0.356 | **0.002** [Hnd; SC] |
| | | awMPD | 0.050 (0.100) | 0.384 [Hnd; SC] |
| | | Maximum alpha PD diff.*Climate diss. (precip.) | 1.002 (0.710) | **0.022** [Hnd; SC] |
| | First growing season | Maximum alpha PD diff. | -0.565 (-0.139) | 0.092 [Hnd; SC] (0.414) |
| | | Disturbed | -0.074 | 0.672 [Hnd; SC] |
| | | Climate diss. (precip.) | 0.297 (0.308) | 0.055 [Hnd; SC] (0.079) |
| | | Climate diss. (temp.) | -0.235 (-0.295) | 0.084 [Hnd; SC] |
| | | Heating | -0.248 | **0.029** [Hnd; SC] |
| | | awMPD | -0.084 (-0.167) | 0.236 [Hnd; SC] |
| | | Maximum alpha PD diff.*Climate diss. (precip.) | 0.702 (0.574) | **0.025** [Hnd; SC] |
| | Overwinter | Maximum alpha PD diff. | -0.191 (-0.117) | 0.523 [Hnd; SC] |
| | | Disturbed | 0.099 | 0.690 [Hnd; SC] |
| | | Climate diss. (precip.) | 0.286 (0.237) | 0.176 [Hnd; SC] |
| | | Climate diss. (temp.) | -0.045 (-0.039) | 0.825 [Hnd; SC] |
| | | Heating | -0.152 | 0.222 [Hnd; SC] |
| | | awMPD | 0.105 (0.211) | 0.196 [Hnd; SC] |
| | Second growing season | Maximum alpha PD diff. | 0.021 (0.013) | 0.958 [Hnd; SC] |
| | | Disturbed | 0.073 | 0.808 [Hnd; SC] |
| | | Climate diss. (precip.) | 0.458 (0.358) | 0.155 [Hnd; SC] |
| | | Climate diss. (temp.) | -0.063 (-0.059) | 0.806 [Hnd; SC] |
| | | Heating | -0.052 | 0.726 [Hnd; SC] |
| | | awMPD | 0.028 (0.056) | 0.799 [Hnd; SC] |
| | Flowering* | Maximum alpha PD diff. | -0.123 (-0.097) | 0.658 [Hnd; SC] |
| | | Climate diss. (precip.) | 0.324 (0.392) | **0.047** [Hnd; SC] |
| | | Climate diss. (temp.) | -0.284 (-0.355) | 0.118 [Hnd; SC] |
| | | Heating | 0.083 | 0.507 [Hnd; SC] |

| | | | | |
|---|---|---|---|---|
| | | awMPD | -0.114 (-0.229) | 0.196 Hnd; SC |

*Plants only flowered in disturbed plots, so these models exclude disturbance

**Table S8**: Summary of results, using *difference in median alpha phylogenetic diversity* (planted species PD minus abundance-weighted community PD) as response variable. Bold indicates significant p-value. In the p-value column, the superscript Hnd indicates the significance (either significant or not significant) was the same in a model using raw phylogenetic diversity (rather than the difference); the superscript SC indicates the significance was the same in the models using single climate variables instead of principal components. In the effect size column, the effect size in brackets is the scaled effect size (centred divided by 2 standard deviations). Categorical variables were not scaled.

| Response | Stage | Explanatory variable | Effect size | p-value |
|---|---|---|---|---|
| **Any plants in plot** | Colonisation | Median alpha PD diff. | 0.170 (0.116) | 0.902 [SC] |
| | | Disturbed | 3.455 (3.668) | **<0.001** [Hnd; SC] |
| | | Climate diss. (precip.) | -1.162 (-1.628) | 0.065 [Hnd; SC] |
| | | Climate diss. (temp.) | 0.230 (0.343) | 0.690 [Hnd; SC] |
| | | Heating | -0.861 | **0.007** [Hnd; SC] |
| | | awMPD | -0.012 (-0.544) | 0.103 [Hnd; SC] |
| | | Median alpha PD diff. * Disturbed | 2.041 (1.385) | **0.039** [Hnd; SC] |
| | First growing season | Median alpha PD diff. | -2.099 (-1.318) | 0.317 [Hnd; SC] |
| | | Disturbed | -0.164 (0.581) | 0.791 [Hnd; SC] (0.330) |
| | | Climate diss. (precip.) | -0.763 (-0.890) | 0.147 [Hnd; SC] |
| | | Climate diss. (temp.) | 0.631 (0.747) | 0.235 [Hnd; SC] |
| | | Heating | -1.003 | **0.008** [Hnd; SC] |
| | | awMPD | 0.005 (0.265) | 0.589 [Hnd; SC] |
| | | Median alpha PD diff. * Disturbed | 5.332 (3.350) | **0.011** [Hnd; SC] |
| | Overwinter | Median alpha PD diff. | -3.712 (0.982) | **0.036** (0.205) |
| | | Disturbed | 1.362 | **0.035** [Hnd] |
| | | Climate diss. (precip.) | -1.728 (-1.933) | **0.020** [Hnd] |
| | | Climate diss. (temp.) | -1.857 (-1.570) | **0.037** |
| | | Heating | 0.805 | 0.064 [Hnd; SC] |
| | | awMPD | -0.333 (-0.666) | 0.240 [Hnd; SC] |
| | | Median alpha PD diff. * Climate diss. (temp.) | 3.901 (3.035) | **0.050** |
| | Second growing season | Median alpha PD diff. | 5.725 (3.272) | **0.034** [Hnd; SC] |
| | | Disturbed | 0.594 (0.066) | 0.417 [Hnd; SC] (0.932) |
| | | Climate diss. (precip.) | -0.680 (-0.544) | 0.360 [Hnd] |
| | | Climate diss. (temp.) | 0.089 (0.084) | 0.864 [Hnd; SC] |

| | | | | |
|---|---|---|---|---|
| | | Heating | -0.867 | **0.046** Hnd; SC |
| | | awMPD | -0.197 (-0.395) | 0.428 Hnd; SC |
| | | Median alpha PD diff. * Disturbed | -6.636 (-3.792) | **0.018** Hnd; SC |
| | Flowering* | Median alpha PD diff. | 0.881 (0.601) | 0.553 Hnd; SC |
| | | Climate diss. (precip.) | -0.811 (-1.137) | 0.351 Hnd; SC |
| | | Climate diss. (temp.) | -0.483 (-0.719) | 0.553 Hnd; SC |
| | | Heating | -0.677 | 0.064 Hnd; SC |
| | | awMPD | 0.019 (0.038) | 0.944 Hnd; SC |
| **Number of plants in plot** | Colonisation | Median alpha PD diff. | 1.377 (0.835) | **0.004** Hnd; SC |
| | | Disturbed | 1.557 | **<0.001** Hnd; SC |
| | | Climate diss. (precip.) | -0.074 (-0.092) | 0.737 Hnd |
| | | Climate diss. (temp.) | -0.364 (-0.413) | 0.121 Hnd; SC |
| | | Heating | -0.318 | **0.004** Hnd; SC |
| | | awMPD | 0.070 (0.141) | 0.218 Hnd; SC |
| | First growing season | Median alpha PD diff. | 0.498 (0.314) | 0.090 Hnd; SC |
| | | Disturbed | -0.050 | 0.772 Hnd; SC |
| | | Climate diss. (precip.) | 0.056 (0.065) | 0.746 Hnd; SC |
| | | Climate diss. (temp.) | -0.255 (-0.320) | 0.079 Hnd; SC |
| | | Heating | -0.241 | **0.029** Hnd; SC |
| | | awMPD | -0.090 (-0.179) | 0.204 Hnd; SC |
| | Overwinter | Median alpha PD diff. | -0.022 (-0.013) | 0.949 Hnd; SC |
| | | Disturbed | 0.123 | 0.617 Hnd; SC |
| | | Climate diss. (precip.) | 0.347 (0.287) | 0.111 Hnd; SC |
| | | Climate diss. (temp.) | -0.069 (-0.060) | 0.732 Hnd; SC |
| | | Heating | -0.149 | 0.238 Hnd; SC |
| | | awMPD | 0.119 (0.238) | 0.147 Hnd; SC |
| | Second growing season | Median alpha PD diff. | -1.177 (0.085) | **0.009** SC (0.730) |
| | | Disturbed | -0.072 | 0.812 Hnd; SC |
| | | Climate diss. (precip.) | -1.028 (-0.585) | 0.052 Hnd; SC |
| | | Climate diss. (temp.) | 0.196 (0.182) | 0.318 Hnd; SC |
| | | Heating | 0.055 | 0.723 Hnd; SC |
| | | awMPD | -0.050 (-0.101) | 0.567 Hnd; SC |
| | | Median alpha PD diff.*Climate diss. (precip.) | 4.047 (1.799) | **<0.001** SC |

| | | | | |
|---|---|---|---|---|
| | Flowering* | Median alpha PD diff. | 0.093 (0.054) | 0.816 Hnd; SC |
| | | Climate diss. (precip.) | 0.309 (0.374) | 0.115 Hnd; SC |
| | | Climate diss. (temp.) | -0.255 (-0.300) | 0.177 Hnd; SC |
| | | Heating | 0.081 | 0.521 Hnd; SC |
| | | awMPD | -0.095 (-0.190) | 0.268 Hnd; SC |

*Plants only flowered in disturbed plots, so these models exclude disturbance

**Table S9**: Summary of results, using *difference in gamma phylogenetic diversity* (planted species PD minus abundance-weighted community PD) as response variable. Bold indicates significant p-value. In the p-value column, the superscript Hnd indicates the significance (either significant or not significant) was the same in a model using raw phylogenetic diversity (rather than the difference); the superscript SC indicates the significance was the same in the models using single climate variables instead of principal components. In the effect size column, the effect size in brackets is the scaled effect size (centred divided by 2 standard deviations). Categorical variables were not scaled. Note that gamma PD was already scaled in the original analysis.

| Response | Stage | Explanatory variable | Effect size | p-value |
|---|---|---|---|---|
| **Any plants in plot** | Colonisation | Gamma PD diff. | 1.608 | **<0.001** $^{Hnd; SC}$ |
| | | Disturbed | 4.501 | **<0.001** $^{Hnd; SC}$ |
| | | Climate diss. (precip.) | -0.608 (-0.851) | 0.335 $^{Hnd; SC}$ |
| | | Climate diss. (temp.) | 0.392 (0.583) | 0.499 $^{Hnd; SC}$ |
| | | Heating | -0.838 | **0.007** $^{Hnd; SC}$ |
| | | awMPD | -0.010 (-0.449) | 0.170 $^{Hnd; SC}$ |
| | | Gamma PD diff. * Disturbed | -1.032 | **<0.001** $^{Hnd; SC}$ |
| | First growing season | Gamma PD diff. | 0.515 | 0.105 $^{Hnd; SC}$ |
| | | Disturbed | 0.578 | 0.322 $^{Hnd; SC}$ |
| | | Climate diss. (precip.) | -0.157 (-0.183) | 0.762 $^{Hnd; SC}$ |
| | | Climate diss. (temp.) | 0.699 (0.827) | 0.190 $^{Hnd; SC}$ |
| | | Heating | -1.015 | **0.007** $^{Hnd; SC}$ |
| | | awMPD | -0.040 (0.081) | 0.867 $^{Hnd; SC}$ |
| | Overwinter | Gamma PD diff. | 0.492 (-0.299) | 0.310 (0.459) |
| | | Disturbed | 1.378 | **0.039** |
| | | Climate diss. (precip.) | -3.286 (-3.673) | **0.001** $^{Hnd; SC}$ |
| | | Climate diss. (temp.) | -0.792 (-0.962) | 0.192 $^{Hnd; SC}$ |
| | | Heating | 0.807 | 0.063 $^{SC}$ |
| | | awMPD | -0.397 (-0.794) | 0.162 $^{Hnd; SC}$ |
| | | Gamma PD diff. * Climate diss. (precip.) | -1.617 (-1.808) | **0.025** $^{Hnd}$ |
| | Second growing season | Gamma PD diff. | 0.131 | 0.643 $^{Hnd; SC}$ |
| | | Disturbed | 0.817 | 0.232 $^{Hnd; SC}$ |
| | | Climate diss. (precip.) | -0.596 (-0.478) | 0.375 $^{Hnd, SC}$ |
| | | Climate diss. (temp.) | 0.139 (0.133) | 0.769 $^{Hnd; SC}$ |
| | | Heating | -0.753 | 0.069 $^{Hnd; SC}$ |
| | | awMPD | -0.061 (-0.122) | 0.782 $^{Hnd; SC}$ |
| | Flowering* | Gamma PD diff. | 0.404 | 0.262 $^{Hnd; SC}$ |

| | | | | |
|---|---|---|---|---|
| | | Climate diss. (precip.) | -0.551 (-0.772) | 0.519 $^{\text{Hnd; SC}}$ |
| | | Climate diss. (temp.) | -0.421 (-0.626) | 0.704 $^{\text{Hnd; SC}}$ |
| | | Heating | -0.615 | 0.079 $^{\text{Hnd; SC}}$ |
| | | awMPD | 0.056 (0.113) | 0.835 $^{\text{Hnd; SC}}$ |
| **Number of plants in plot** | Colonisation | Gamma PD diff. | 0.024 | 0.840 $^{\text{Hnd; SC}}$ |
| | | Disturbed | 1.562 | **<0.001** $^{\text{Hnd; SC}}$ |
| | | Climate diss. (precip.) | 0.115 (0.144) | 0.616 $^{\text{Hnd; SC}}$ |
| | | Climate diss. (temp.) | -0.422 (-0.479) | 0.111 $^{\text{Hnd; SC}}$ |
| | | Heating | -0.348 | **0.003** $^{\text{Hnd; SC}}$ |
| | | awMPD | 0.050 (0.099) | 0.392 $^{\text{Hnd; SC}}$ |
| | First growing season | Gamma PD diff. | 0.146 | 0.126 $^{\text{Hnd; SC}}$ |
| | | Disturbed | 0.023 | 0.897 $^{\text{Hnd; SC}}$ |
| | | Climate diss. (precip.) | 0.210 (0.243) | 0.176 $^{\text{Hnd; SC}}$ |
| | | Climate diss. (temp.) | -0.277 (-0.285) | 0.118 $^{\text{Hnd; SC}}$ |
| | | Heating | -0.240 | **0.036** $^{\text{Hnd; SC}}$ |
| | | awMPD | -0.084 (-0.167) | 0.240 $^{\text{Hnd; SC}}$ |
| | Overwinter | Gamma PD diff. | 0.032 | 0.747 $^{\text{Hnd; SC}}$ |
| | | Disturbed | 0.142 | 0.573 $^{\text{Hnd; SC}}$ |
| | | Climate diss. (precip.) | 0.369 (0.305) | 0.088 $^{\text{Hnd; SC}}$ |
| | | Climate diss. (temp.) | -0.074 (-0.065) | 0.710 $^{\text{Hnd; SC}}$ |
| | | Heating | -0.144 | 0.250 $^{\text{Hnd; SC}}$ |
| | | awMPD | 0.122 (0.245) | 0.123 $^{\text{Hnd; SC}}$ |
| | Second growing season | Gamma PD diff. | 0.175 | 0.141 $^{\text{Hnd; SC}}$ |
| | | Disturbed | 0.159 | 0.588 $^{\text{Hnd; SC}}$ |
| | | Climate diss. (precip.) | 0.612 (0.478) | **0.037** $^{\text{Hnd; SC}}$ |
| | | Climate diss. (temp.) | -0.096 (-0.089) | 0.678 $^{\text{SC}}$ |
| | | Heating | -0.049 | 0.742 $^{\text{Hnd; SC}}$ |
| | | awMPD | 0.050 (0.100) | 0.621 $^{\text{Hnd; SC}}$ |
| | Flowering* | Gamma PD diff. | -0.005 | 0.960 $^{\text{Hnd; SC}}$ |
| | | Climate diss. (precip.) | 0.332 (0.401) | **0.048** $^{\text{Hnd; SC}}$ |
| | | Climate diss. (temp.) | -0.269 (-0.317) | 0.155 $^{\text{Hnd; SC}}$ |
| | | Heating | 0.078 | 0.533 $^{\text{Hnd; SC}}$ |
| | | awMPD | -0.100 (-0.200) | 0.233 $^{\text{Hnd; SC}}$ |

*Plants only flowered in disturbed plots, so these models exclude disturbance

*Supplementary tables for species origin and traits*

**Table S10**: Difference in model performance when adding species status (native/non-native) and biogeographic origin (native continent syndrome of Fristoe et al. 2023) into the main models testing the effect of PD. Positive values of ΔAIC indicate the model is *worse* when including species status or origin.

| **Research question** | **Model** | **ΔAIC when adding native/exotic** | **ΔAIC when adding origin** | **Affect significance or direction of PD?** |
|---|---|---|---|---|
| **Question 1 (absolute PD)** | Colonisation (any plants in plot) | -0.4 | 2.0 | No |
| | First growing season (any plants in plot) | 2.0 | 1.9 | No |
| | Overwinter (any plants in plot) | -3.2 | 2.0 | No |
| | Second growing season (any plants in plot) | -2.3 | 1.2 | No |
| | Colonisation (number of plants) | -2.2 | 1.9 | No |
| | First growing season (number of plants) | 1.7 | -0.9 | Yes* |
| | Overwinter (number of plants) | 1.8 | 2.0 | No |
| | Second growing season (number of plants) | 0.3 | -1.5 | No |
| **Question 2 (relative PD)** | Colonisation (any plants in plot) | 1.2 | 2.0 | No |
| | First growing season (any plants in plot) | 1.3 | 2.0 | No |
| | Overwinter (any plants in plot) | 0.7 | -0.4 | No |
| | Second growing season (any plants in plot) | 0.7 | 2.0 | No |
| | Colonisation (number of plants) | -5.1 | 2.0 | No |
| | First growing season (number of plants) | 1.5 | -0.3 | No |
| | Overwinter (number of plants) | 1.7 | 2.0 | No |
| | Second growing season (number of plants) | 1.4 | 0.1 | No |

*Including biogeographic origin removed the PD*disturbance interaction, but the main effect of PD was still positive and significant (p = 0.043).

**Table S11**: Difference in structural equation model performance when adding the traits [SLA + height] or [SLA + height + seed mass]. AIC values were compared between models including only the indirect effects of indigenous-PD (i.e. the effect of indigenous-PD is mediated entirely through its effect on the included traits; Fig S8b), versus models that include both indirect effects and direct effects of indigenous-PD (Fig. S8a). Positive values of ΔAIC indicate the model is *worse* when including indirect effects of indigenous-PD only; in other words, positive values of ΔAIC indicate Fig. S8a is preferred. Only models where indigenous-PD was found to be significant in the main models were tested (see Fig. 3b-d and Fig. 5a-d in main text); note that models from the second year where indigenous-PD was found to be significant are not included due to too few data to appropriately parameterise SEMs.

| **Research question** | **Model** | **Traits included** | **ΔAIC when only including indirect effects (effect of PD entirely through traits)** | **PD p-value when including traits** |
|---|---|---|---|---|
| **Question 1 (absolute PD)** | First growing season (any plants in plot) | SLA + Height | 6.2 | **0.028** |
| | | SLA + Height + Seed mass | 13.0 | **0.001** |
| | Colonisation (number of plants) | SLA + Height | -1.5 | **<0.001** |
| | | SLA + Height + Seed mass | 1.3 | **<0.001** |
| | First growing season (number of plants) | SLA + Height | 6.2 | **<0.001** |
| | | SLA + Height + Seed mass | 7.4 | **<0.001** |
| **Question 2 (relative PD)** | Colonisation (any plants in plot) | SLA + Height | 2.5 | 0.103* |
| | | SLA + Height + Seed mass | 2.4 | 0.111* |
| | First growing season (any plants in plot) | SLA + Height | 5.4 | **0.025** |
| | | SLA + Height + Seed mass | 5.1 | **0.029** |
| | Colonisation (number of plants) | SLA + Height | 2.9 | 0.087* |
| | | SLA + Height + Seed mass | 2.2 | 0.124* |
| | First growing season (number of plants) | SLA + Height | 1.3 | 0.189* |
| | | SLA + Height + Seed mass | 1.5 | 0.172* |

*While PD was no longer significant in these models, this was a product of the lower number of datapoints necessitated by only including species with relevant trait data – these values were also non-significant when model structure was the same as the main models i.e. not including traits at all.

**Table S12**: Relationship between traits and indigenous-PD for all four PD metrics. Competition index and resistance to herbivory was only available for 62 and 63 of the 93 species included in the Kempel study. Superscript numbers refer to data sources which follow at the end of the table. Three tests per PD metric were performed: using all available data ("all data"); limiting data to species that were native to Europe or North America ("EU/NA"); and including biogeographic syndrome as a random variable ("BG RV").

| Trait | Number of species | Metric | p-value (all data) | p-value (EU/NA) | p-value (BG RV) |
|---|---|---|---|---|---|
| **Seed mass (mg)** | 153 [1, 2, 3] | Mean alpha PD | **0.036** | 0.053 | **0.028** |
| | | Maximum alpha PD | **0.042** | **0.049** | **0.039** |
| | | Median alpha PD | **0.024** | **0.009** | **0.022** |
| | | Gamma PD | 0.694 | 0.998 | 0.754 |
| **Optimal germination rate** | 151 [1, 2] | Mean alpha PD | 0.180 | 0.192 | 0.180 |
| | | Maximum alpha PD | 0.749 | 0.619 | 0.749 |
| | | Median alpha PD | 0.955 | 0.932 | 0.955 |
| | | Gamma PD | 0.172 | 0.097 | 0.172 |
| **Competition index** | 62 [2] | Mean alpha PD | 0.516 | 0.841 | 0.507 |
| | | Maximum alpha PD | 0.491 | 0.681 | 0.478 |
| | | Median alpha PD | 0.351 | 0.694 | 0.337 |
| | | Gamma PD | 0.435 | 0.524 | 0.424 |
| **Resistance to herbivory** | 63 [2] | Mean alpha PD | 0.107 | 0.111 | 0.111 |
| | | Maximum alpha PD | 0.363 | 0.499 | 0.416 |
| | | Median alpha PD | 0.780 | 0.567 | 0.760 |
| | | Gamma PD | 0.571 | 0.685 | 0.479 |
| **Hardiness Index** | 63 [1] | Mean alpha PD | 0.396 | 0.271 | 0.674 |
| | | Maximum alpha PD | 0.186 | **0.037** | 0.129 |
| | | Median alpha PD | **0.014** | 0.109 | **0.029** |
| | | Gamma PD | 0.190 | 0.158 | **0.042** |
| **Life history (annual/perennial)** | 63 [1] | Mean alpha PD | 0.803 | 0.780 | 0.637 |
| | | Maximum alpha PD | 0.267 | 0.953 | 0.246 |
| | | Median alpha PD | 0.710 | 0.353 | 0.848 |
| | | Gamma PD | 0.212 | 0.252 | 0.216 |
| **Vegetative height (m)** | 149 [3, 4] | Mean alpha PD | 0.185 | 0.279 | 0.166 |
| | | Maximum alpha PD | **0.001** | **<0.001** | **0.001** |
| | | Median alpha PD | 0.259 | 0.819 | 0.126 |
| | | Gamma PD | 0.137 | 0.073 | 0.184 |

| | | | | | |
|---|---|---|---|---|---|
| **Generative height (m)** | 103 [3] | Mean alpha PD | 0.533 | 0.594 | 0.514 |
| | | Maximum alpha PD | **0.001** | **<0.001** | **0.005** |
| | | Median alpha PD | 0.421 | 0.251 | 0.922 |
| | | Gamma PD | 0.280 | 0.144 | 0.619 |
| **Leaf nitrogen (mg/g)** | 70 [3] | Mean alpha PD | **0.038** | **0.010** | **0.032** |
| | | Maximum alpha PD | 0.788 | 0.596 | 0.751 |
| | | Median alpha PD | 0.163 | 0.270 | 0.353 |
| | | Gamma PD | **0.030** | **0.014** | 0.111 |
| **SLA ($mm^2$/mg)** | 133 [3, 5, 6, 7, 8] | Mean alpha PD | 0.134 | 0.257 | 0.129 |
| | | Maximum alpha PD | 0.190 | 0.243 | 0.184 |
| | | Median alpha PD | 0.835 | 0.750 | 0.833 |
| | | Gamma PD | **0.006** | **0.025** | **0.005** |
| **LDMC (g/g)** | 100 [3] | Mean alpha PD | **0.012** | **0.013** | **0.011** |
| | | Maximum alpha PD | 0.686 | 0.754 | 0.899 |
| | | Median alpha PD | **0.023** | 0.062 | **0.019** |
| | | Gamma PD | 0.504 | 0.593 | 0.530 |

[1] Haeuser et al. 2017
[2] Kempel et al. 2013
[3] Kattge et al. 2020
[4] Falster et al. 2021
[5] Wang et al. 2017
[6] Anten & Hirose 1999
[7] Elkins & van Iersel 2020
[8] Wang et al. 2024

Supplementary References


Anten, N. P., & Hirose, T. (1999). Interspecific differences in above-ground growth patterns result in spatial and temporal partitioning of light among species in a tall-grass meadow. *Journal of Ecology*, *87*(4), 583-597.

Belsky, A. J. (1994). Influences of trees on savanna productivity: tests of shade, nutrients, and tree-grass competition. *Ecology*, *75*(4), 922-932.

BGCI (2026). GlobalTreeSearch. Botanic Gardens Conservation International. Richmond, U.K. Available at https://tools.bgci.org/global_tree_search.php. Accessed on 16/02/2016.

Bond, W. J., & Midgley, G. F. (2012). Carbon dioxide and the uneasy interactions of trees and savannah grasses. *Philosophical Transactions of the Royal Society B: Biological Sciences*, *367*(1588), 601-612.

Carmona, C. P., Bueno, C. G., Toussaint, A., Träger, S., Díaz, S., Moora, M., ... & Tamme, R. (2021). Fine-root traits in the global spectrum of plant form and function. *Nature*, *597*(7878), 683-687.

Czyżewski, S., Søndergaard, S. A., Molnár, Á. P., Kerr, M. R., Kristensen, J. A., Atkinson, J., ... & Svenning, J. C. (2026). Revisiting Europe's temperate forests: Palaeoecological evidence for an herbivory-driven woodland-grassland mosaic biome. *Biological Conservation*, *316*, 111749.

Díaz, S., Kattge, J., Cornelissen, J. H., Wright, I. J., Lavorel, S., Dray, S., ... & Gorné, L. D. (2016). The global spectrum of plant form and function. *Nature*, *529*(7585), 167-171.

Dohn, J., Dembélé, F., Karembé, M., Moustakas, A., Amévor, K. A., & Hanan, N. P. (2013). Tree effects on grass growth in savannas: competition, facilitation and the stress-gradient hypothesis. *Journal of Ecology*, *101*(1), 202-209.

Dullinger, I., Wessely, J., Bossdorf, O., Dawson, W., Essl, F., Gattringer, A., ... & Dullinger, S. (2017). Climate change will increase the naturalization risk from garden plants in Europe. *Global Ecology and Biogeography*, *26*(1), 43-53.

Elkins, C., & van Iersel, M. W. (2020). Longer photoperiods with the same daily light integral improve growth of rudbeckia seedlings in a greenhouse. *HortScience*, *55*(10), 1676-1682.

Falster, D., Gallagher, R., Wenk, E. H., Wright, I. J., Indiarto, D., Andrew, S. C., ... & O'sullivan, O. S. (2021). AusTraits, a curated plant trait database for the Australian flora. *Scientific Data*, *8*(1), 254.

Fristoe, T. S., Bleilevens, J., Kinlock, N. L., Yang, Q., Zhang, Z., Dawson, W., ... & van Kleunen, M. (2023). Evolutionary imbalance, climate and human history jointly shape the global biogeography of alien plants. *Nature Ecology & Evolution*, *7*(10), 1633-1644.

Gelman, A. (2008). Scaling regression inputs by dividing by two standard deviations. *Statistics in Medicine*, *27*(15), 2865-2873.

Gorné, L. D., Aguirre-Gutiérrez, J., Souza, F. C., Swenson, N. G., Kraft, N. J. B., Schwantes Marimon, B., ... & Díaz, S. (2025). Use and misuse of trait imputation in ecology: the problem of using out-of-context imputed values. *Ecography*, *2025*(6), e07520.

Grenié, M., Bruelheide, H., Dawson, W., Essl, F., van Kleunen, M., Kühn, I., ... & Winter, M. (2025). Gaps in global alien plant trait data and how to fill them. *Global Ecology and Biogeography*, *34*(10), e70131.

Haeuser, E., Dawson, W., & van Kleunen, M. (2017). The effects of climate warming and disturbance on the colonization potential of ornamental alien plant species. *Journal of Ecology*, *105*(6), 1698-1708.

Kattge, J., Bönisch, G., Díaz, S., Lavorel, S., Prentice, I. C., Leadley, P., ... & Cuntz, M. (2020). TRY plant trait database–enhanced coverage and open access. *Global Change Biology, 26*(1), 119-188.

Kempel, A., Chrobock, T., Fischer, M., Rohr, R. P., & van Kleunen, M. (2013). Determinants of plant establishment success in a multispecies introduction experiment with native and alien species. *Proceedings of the National Academy of Sciences*, 110(31), 12727-12732.

Lefcheck, J. S. (2016). piecewiseSEM: Piecewise structural equation modelling in r for ecology, evolution, and systematics. *Methods in Ecology and Evolution*, *7*(5), 573-579.

Ludwig, F., Dawson, T. E., Prins, H. H. T., Berendse, F., & De Kroon, H. (2004). Below-ground competition between trees and grasses may overwhelm the facilitative effects of hydraulic lift. *Ecology letters*, *7*(8), 623-631.

Moles, A. T., Ackerly, D. D., Tweddle, J. C., Dickie, J. B., Smith, R., Leishman, M. R., ... & Westoby, M. (2007). Global patterns in seed size. *Global Ecology and Biogeography*, *16*(1), 109-116.

Revell, L. J. (2024). phytools 2.0: an updated R ecosystem for phylogenetic comparative methods (and other things). *PeerJ*, *12*, e16505.

Riginos, C. (2009). Grass competition suppresses savanna tree growth across multiple demographic stages. *Ecology*, *90*(2), 335-340.

Scholes, R. J., & Archer, S. R. (1997). Tree-grass interactions in savannas. *Annual Review of Ecology and Systematics*, *28*(1), 517-544.

Silander Jr, J. A., Bond, W. J., & Ratsirarson, J. (2024). The grassy ecosystems of Madagascar in context: Ecology, evolution, and conservation. *Plants, People, Planet*, *6*(1), 94-115.

Smith, S. A., & Brown, J. W. (2018). Constructing a Broadly Inclusive Seed Plant Phylogeny. *American Journal of Botany*, *105*, 302–314.

Stachowicz, J. J., & Tilman, D. (2005). Species invasions and the relationships between species diversity, community saturation, and ecosystem functioning. *Species invasions: insights into ecology, evolution, and biogeography*, 41-64.

Strömberg, C. A. (2011). Evolution of grasses and grassland ecosystems. *Annual Review of Earth and Planetary Sciences*, *39*(1), 517-544.

van Kleunen, M., Xu, X., Yang, Q., Maurel, N., Zhang, Z., Dawson, W., ... & Fristoe, T. S. (2020). Economic use of plants is key to their naturalization success. *Nature Communications*, *11*(1), 3201.

Wang, C., Zhou, J., Liu, J., & Jiang, K. (2017). Differences in functional traits between invasive and native *Amaranthus* species under different forms of N deposition. *The Science of Nature*, *104*(7), 59.

Wang, F., Zhang, Q., Huang, P., Li, C., & Li, Y. (2024). CSR strategy composition and leaf traits for herbaceous plants in garden design. *Ecological Indicators*, *158*, 111173.

Yang, Q., Weigelt, P., Fristoe, T. S., Zhang, Z., Kreft, H., Stein, A., ... & van Kleunen, M. (2021). The global loss of floristic uniqueness. *Nature Communications*, *12*(1), 7290.
